# Interleukin 27, like interferons, activates JAK-STAT signaling and promotes pro-inflammatory and antiviral states that interfere with dengue and chikungunya viruses replication in human macrophages

**DOI:** 10.3389/fimmu.2024.1385473

**Published:** 2024-04-24

**Authors:** Juan Felipe Valdés-López, Lady Johana Hernández-Sarmiento, Y. S. Tamayo-Molina, Paula A. Velilla-Hernández, Izabela A. Rodenhuis-Zybert, Silvio Urcuqui-Inchima

**Affiliations:** ^1^ Grupo Inmunovirología, Facultad de Medicina, Universidad de Antioquia UdeA, Medellín, Colombia; ^2^ Department of Medical Microbiology and Infection Prevention, University of Groningen and University Medical Center Groningen, Groningen, Netherlands

**Keywords:** interleukin 27, interferons, JAK-STAT signaling, interferon-stimulated genes, antiviral response, pro-inflammatory response, viruses

## Abstract

Interferons (IFNs) are a family of cytokines that activate the JAK-STAT signaling pathway to induce an antiviral state in cells. Interleukin 27 (IL-27) is a member of the IL-6 and/or IL-12 family that elicits both pro- and anti-inflammatory responses. Recent studies have reported that IL-27 also induces a robust antiviral response against diverse viruses, both *in vitro* and *in vivo*, suggesting that IFNs and IL-27 share many similarities at the functional level. However, it is still unknown how similar or different IFN- and IL-27-dependent signaling pathways are. To address this question, we conducted a comparative analysis of the transcriptomic profiles of human monocyte-derived macrophages (MDMs) exposed to IL-27 and those exposed to recombinant human IFN-α, IFN-γ, and IFN-λ. We utilized bioinformatics approaches to identify common differentially expressed genes between the different transcriptomes. To verify the accuracy of this approach, we used RT-qPCR, ELISA, flow cytometry, and microarrays data. We found that IFNs and IL-27 induce transcriptional changes in several genes, including those involved in JAK-STAT signaling, and induce shared pro-inflammatory and antiviral pathways in MDMs, leading to the common and unique expression of inflammatory factors and IFN-stimulated genes (ISGs)Importantly, the ability of IL-27 to induce those responses is independent of IFN induction and cellular lineage. Additionally, functional analysis demonstrated that like IFNs, IL-27-mediated response reduced chikungunya and dengue viruses replication in MDMs. In summary, IL-27 exhibits properties similar to those of all three types of human IFN, including the ability to stimulate a protective antiviral response. Given this similarity, we propose that IL-27 could be classified as a distinct type of IFN, possibly categorized as IFN-pi (IFN-π), the type V IFN (IFN-V).

## 1 Introduction

In 1957, using a system of influenza A virus (IAV) grown in chicken embryo chorioallantoic membranes, Isaacs and Lindenmann (1) revealed that a protein secreted by cells stimulated with heat-inactivated IAV inhibited the replication of infectious IAV in cells. The authors named the antiviral factor interferon (IFN) because of its ability to “interfere” with IAV replication into the cells. IFNs are a family of soluble cytokines. They play a crucial role in the establishment of an antiviral state and activating the immune system, which leads to the upregulation of effector antiviral proteins (AVPs) encoded by IFN-stimulated genes (ISGs). Numerous studies have explored the roles of various protein families in controlling different steps of viral replication cycle. These include Apolipoprotein B Editing Complex 3 (APOBEC3)-, guanylate-binding protein (GBP)-, IFN-inducible (IFI)-, IFN-induced proteins with tetratricopeptide repeats (IFIT)-, IFN-induced transmembrane (IFITM)-, dynamin-like GTPase (MX)-, and oligoadenylate synthase (OAS)-family proteins, together with indoleamine-pyrrole 2,3-dioxygenase 1 (IDO1), ISG15, ISG20, double-stranded RNA-activated protein kinase R (PKR), tetherin, viperin, and others. These AVPs function at various stages of viral replication, including viral entry, replication, assembly, budding, and their spread into the tissues (2–6). Although the field has mostly focused on ISGs/AVPs, current knowledge indicates that IFNs exhibit a broad spectrum of functions. These functions include regulating the activity of the immune system by inducting pro-inflammatory factors, such as cytokines and chemokines. These factors, in turn, mediate the recruitment and activation of various immune cell populations at the site of infection (7–10). Furthermore, IFNγ modulates the expression of costimulatory molecules and major histocompatibility complex class I (MHC-I) and class II (MHC-II) in antigen-presenting cells (APCs), promoting the antigen presentation to T cells and links the innate immune system to the adaptive immune response (11–13). Based on the degree of sequence homology, structural features, receptor usage, and biological effects, the IFN family has been divided into four types. In humans, type I IFN (IFN-I) subfamily is comprise by 13 subtypes of IFN-alpha (IFN-α), and a single IFN-beta (IFN-β), IFN-epsilon (IFN-ε), IFN-kappa (IFN-κ), and IFN-omega (IFN-ω). All IFN-I share a heterodimeric receptor (IFNAR) consisting of the IFNAR1 and IFNAR2 (3, 13, 14). Type II IFN (IFN-II) is only represented by IFN-gamma (IFN-γ) that signals through IFN-γ-receptor complex (IFNGR), consisting of the IFNGR1 and IFNGR2 (3, 15). Type III IFN (IFN-III) is classified into IFN-lambda 1 (IFN-λ1)/IL-29, IFN-λ2/IL-28A, IFN-λ3/IL-28B, and IFN-λ4 (a pseudogene) that bind to heterodimeric IFN-λ/IL-28 receptor (IFNLR/IL-28R), consistent of the IL-10RB and IFNLR chain (IL28RA) (14, 16). Type IV IFN (IFN-IV) consisting of the IFN-upsilon (IFN-υ) was recently identified from the genomic sequences of vertebrates (17). IFN-υ binds to IFN-υ-receptor complex (IFNUR) consisting of the IFNUR1 and IL-10RB, and is associated with antiviral activity in zebrafish (*Danio rerio*) and African clawed frog (*Xenopus laevis*). However, IFN-υ and IFNUR1 genes were lost during the evolution of superior mammals, including humans (17).

The interaction between type I and III IFNs and their receptors triggers the activation of Janus kinase 1 (JAK-1) and tyrosine kinase 2 (TYK-2). Subsequently, JAK-1 and TYK-2 phosphorylate and activate signal transducer and activator of transcription (STAT)-1 and STAT-2. These activated STAT proteins, along with IFN regulatory factor 9 (IRF-9), form a transcription factor complex known as IFN-stimulated gene factor 3 (ISGF-3) (2, 14, 15). After activation, this complex is translocated into the nucleus, where it binds to IFN-stimulated response elements (ISRE) to coordinate the expression of a large number of IFN-stimulated genes (ISGs), which play a crucial role in orchestrating the antiviral state in cells (2, 3, 15, 16). In contrast, the IFN-II/IFNGR interaction leads to the activation of JAK-1 and JAK-2, which phosphorylate and activate STAT-1, and to a lesser extent, STAT-3 (17). Activated STAT-1 forms a homodimer called IFNG-activated factor (GAF), which binds to IFNG-activated sequence (GAS) elements in the promoter of IFNG-responsive genes (18). This process orchestrates a pro-inflammatory response and antiviral state in cells.

Numerous studies have focused on determining the role of IFNs in inducing the expression of AVPs/ISGs. However, an additional question arises: “Are IFNs the sole factors responsible for AVPs/ISGs expression?” Recent research by ourselves and others has uncovered interleukin 27 (IL-27) as a novel antiviral cytokine. It also induces AVPs/ISGs expression, leading to a robust antiviral response against various viruses, including human immunodeficiency virus type 1 (HIV-1) (19), human herpes simplex virus 1 (HSV1) (20), hepatitis B virus (HBV) (21), HCV (22), IAV (23), zika virus (ZIKV) (24, 25), chikungunya virus (CHIKV) (26), and severe acute respiratory syndrome coronavirus 2 (SARS-CoV-2) (27). These results suggest that IL-27 plays an important role in the control of acute and chronic viral infections. However, our understanding of the molecular basis of the IL-27-dependent antiviral response is still in its early stages. Therefore, elucidating the mechanisms by which ISG expression is regulated remains an important area of study.

IL-27 is a heterodimeric cytokine belonging to the IL-6 and/or IL-12 family of cytokines (24, 25). It consists of Epstein-Barr virus-induced 3 (EBI-3) and IL27-p28 subunits (26, 27). EBI-3, a secreted glycoprotein and member of the hematopoietic receptor family, is closely related to IL12-p40 (28). Its expression is regulated by the activation of MyD88-dependent Toll-like receptors [TLR-2-complex (TLR-1, -2, and -6), TLR-4, -5, -7, -8, and -9] and nuclear factor kappa B (NF-kB) signaling pathways in macrophages and dendritic cells (29, 30). IL27-p28, also known as IL-30, can be secreted as an independent monomer with its own functions, some of which are similar to IL-27 but independent of EBI-3 (31). Both IL-27 subunits are subject to distinct transcriptional regulation mechanisms. Unlike EBI-3, IL27-p28 expression is regulated by the activation of IRF-1, IRF-3 and NF-kB (acting as a possible enhancer). This regulation occurs in response to TIR-domain-containing adapter-inducing IFN-β (TRIF)-dependent TLRs (TLR-3, and TLR-4) as well as IFN-I/ISGF-3/IRF-1-dependent signaling in macrophages and dendritic cells (28, 29, 32). IL-27 signaling occurs through a quaternary complex involving the IL-27 ligand (IL-27-p28/EBI-3) and the IL-27 receptor, composed of IL-27Rα (WSX-1), and Interleukin 6 cytokine family signal transducer (IL-6ST/gp130). Like to IFN-II, IL-27 triggers the activation of JAK-1 and JAK-2, leading to the phosphorylation and activation the STAT-1 and STAT-3 transcription factors (22, 33–35). IL-27 plays various roles, such as eliciting anti-inflammatory responses by promoting the functionality of regulatory T (Treg) cells and inducing the production of IL-10 by T helper 1 (Th1) and Th17 cells, in order to prevent exacerbated inflammation, tissues injuries, organs dysfunction, and/or autoimmunity (29–33). However, IL-27 also promotes pro-inflammatory responses by enhancing IFN-γ production through the activation of CD4+ T cells and natural killer (NK) cells (34, 35). Further, IL-27 induces Th1 and downregulates Th17 response in CD4+ T cells (36, 37). Another function of IL-27 is to act on naïve CD8+ T cells, enhancing the generation of cytotoxic T lymphocytes (38), and supporting the function of the germinal center and B cells by enhancing the activity of T follicular helper (Tfh) cells and production of IL-21 (19). Furthermore, IL-27 signaling promotes effector pro-inflammatory and antiviral responses in both monocytes and macrophages (39–41). In addition, it induces a synergistic effect with IL-15 on NK cells, enhancing the clearing and killing of virus-infected cells (35, 42). Taken together, these reports demonstrate the role of IL-27 as a regulator of different immune system functions. However, in recent years a little-known biological function of IL-27 has been described; its ability to induce an IFN-like signature in different human cell populations, which in turn inhibit viruses replication (25, 26, 43, 44), suggesting that at a functional level IL-27 shares many similarities with human IFNs. However, the precise mechanisms through which these cytokines signal, and the degree to which they operate dependently or independently, are still not fully understood. Therefore, the aim of this study was to conduct a comparative analysis of the transcriptional profiles of IFN-I (IFN-α and -β), IFN-II (IFN-γ), IFN-III (IFN-λ), and IL-27 in human macrophages. Furthermore, we assessed their antiviral activities against dengue virus 2 (DENV-2) and chikungunya virus (CHIKV).

## 2 Materials and methods

### 2.1 Ethics statement

The study was approved by the Ethics Committee of the “Sede de Investigación Universitaria-Universidad de Antioquia.” Written informed consent was obtained from all individuals who voluntarily participated in this study according to the principles of the Declaration of Helsinki. In this study, four healthy donors were included.

### 2.2 Culture of human monocytes and differentiation into monocytes-derived macrophages

Human peripheral blood mononuclear cells (PBMCs) from blood samples of healthy donors mixed with 2% EDTA were isolated using a density gradient with Lymphoprep (STEMCELL Technologies Inc., Vancouver, Canada) by centrifugation (850 × g for 21 min), as previously described (45). PBMCs from each healthy volunteer were prepared independently. Platelet depletion was performed by washing with PBS 1X (Sigma-Aldrich, MisuriMissouri, USA) three times at 250 × g for 10 min, and the percentage of CD14 positive cells was determined by flow cytometry. To obtain monocytes, 24-well plastic plates were scratched with a 1000 μL pipette tip, seeded with 5x10^5^ CD14 positive cells per well, and allowed to adhere for 2 h in RPMI-1640 medium supplemented with 0.5 (v/v) autologous serum or plasma (to favor adherence of monocytes to the well), 4 mM L-glutamine (Sigma-Aldrich), 0.3% (v/v) sodium carbonate (NaCO_3_; Sigma-Aldrich), and cultured at 37°C and 5% CO_2_. Non-adherent cells were removed by washing twice with PBS 1X, and monocytes were differentiated to macrophages by adherence to the plastic without macrophage colony-stimulating factor (M-CSF), in RPMI-1640 medium supplemented at 10% (v/v) heat-inactivated fetal bovine serum (FBS; Gibco, Thermo Fisher Scientific, Massachusetts, USA), 4 mM L-glutamine, 0.3% (v/v) NaCO_3_, and 1% (v/v) Corning™ antibiotic-antimycotic solution (Corning-Cellgro, New York, USA) (complete medium), and incubated at 37°C and 5% CO_2_. Medium change was performed every 2 days, and until seventh day of cell differentiation to obtain FBS-MDMs, as previously described (26, 45, 46).

### 2.3 Microscopy

FBS-MDMs cultures were visualized and photographed using an Axio Vert.A1 microscope (ZEISS, Oberkochen, Germany) equipped with a Ds-fi1 camera (Nikon, Tokyo, Japan) and adapted for transmitted light in both phase-contrast and epi-fluorescence microscope. To analyze the nuclear-cytoplasmic ratio, FBS-MDMs cultures were stained with SYBR Green 10000X (diluted 1:1500; Invitrogen) for 30 min at 37°C, as we previously reported (46). The images were processed and analyzed using the software IrfanView (version 4.69), ImageJ (version 1.8.0), and GIMP (version 2.10.30).

### 2.4 Flow cytometry analysis

A BD-LSRFortessa flow cytometer (BD Biosciences, New Jersey, USA) was utilized to determine the cell size (FSC-A) and cytoplasmic complexity/granularity (SSC-A) of FBS-MDMs. To quantify the expression of cell surface markers in macrophages, FBS-MDM cultures (n= 4) were stimulated or not with 25 ng/ml of IFN-β, IFN-γ, IFN-λ1, or IL-27 (BioLegend) for 18 h. The cells were then stained with the fixable viability dye eFluor-506 (diluted 1:1000; eBiosciences) for 20 min at 4°C. Next, the cells were stained with anti-human CD38 (PE, clone: HIT2), anti-human CD40 (AF700, clone: 5C3), anti-human CD68 (PE, clone: eBIOY1/82A), anti-human CD80 (FITC, clone: 2D10.4), and/or anti-human CD86 (PE, clone: FUN-1) antibodies (eBioscience, Massachusetts, USA, and BD Bioscience), or the relevant isotypes in PBS 1X at 4°C in the dark. We did not block FC-receptors during antibody staining. Then, the cells were washed by centrifugation and resuspended in PBS 1X. Positively labeled cells were defined based on isotype controls and unstained cells, and a compensation matrix was created to compensate for spectral overlap using single staining. At least 20,000 events were acquired per sample, and data were analyzed using FlowJo software (version 7.6.2). Debris and dead cells were gated out using forward and side light scatter, as well as the fixable viability dye eFluor-506 staining. Cell viability was confirmed to be higher than 90% for all experiments.

### 2.5 RNA extraction, RNA-Seq data, and bioinformatics analysis

FBS-MDM cultures (n= 3) were stimulated or not with 25 ng/mL recombinant human IL-27 (BioLegend, California, USA). Total RNA from unstimulated FBS-MDMs (control) and IL-27-stimulated FBS-MDM was obtained using Direct-zol™ RNA Miniprep Plus (Zymo Research, California, USA) following the manufacturer’s protocol. RNA samples were treated with a DNase I column (Zymo Research) to remove contaminating genomic DNA. RNA was quantified by spectrophotometry using a nanodrop (Thermo Scientific, Massachusetts, USA), and 1μg of RNA was used for bulk RNA-Seq, which was carried out on an Illumina HiSeq 2000 platform (Macrogen, Seoul, South Korea). After sequencing, the image data were transformed into raw reads and stored in FASTQ datasets for each sample, using FastQC (google/vFqiZ), as previously described (26). Clean reads were obtained by removing low-quality adapter, poly N-containing, and shorter-than-70 bp reads. The location of the reads on the reference genome was determined quickly and precisely by comparing reads with the reference genome (GRCh38) using the HISAT2 software (47). The new transcripts were then assembled using String Tie software (48), the feature count tool in Subread software (49), and the raw count number in each sample was obtained. Next, to perform a comparative analysis of the transcriptional profiles induced by IFNs and IL-27 in human MDMs, we merged our RNA-Seq data from unstimulated and IL-27-stimulated FBS-MDMs with the publicly available RNA-seq dataset GSE158434 (GEO) (50). In this dataset, human monocytes from healthy donors (n= 3) were cultured in RPMI-1640 medium supplemented with 10% human AB serum (HS) and differentiated into MDMs by adherence to plastic for 7 days (HS-MDMs). HS-MDMs were then stimulated with 25 ng/mL recombinant human IFN-α, IFN-γ, or IFN-λ for 18 h, and mRNA sequencing was performed by bulk RNA-seq.

To determine the differentially expressed genes (DEG), we selected genes with a p-val< 0.05, and |Log2 Fold Change (IFN or IL-27-stimulated MDMs/Unstimulated MDMs) |> 0.6 (|log2FC|> 0.6), using the DESeq2 library (51). Common DEG between transcriptomes were identified using InteractiVenn (52), drawing a Venn diagram. Gene enrichment analysis were carried out with the Cluster Profiler (Version 4.0) (53). Then, the raw counts of GSE158434 and our RNA-seq were merged and normalized to transcript per million (TPM) using R statistical software (version 4.2.0). We selected the genes linked to JAK-STAT signaling, pattern recognition receptors (PRRs) signaling, both antiviral and inflammatory responses, and antigen presentation with the entrezID using the Kyoto Encyclopedia of Genes and Genomes (KEGG), and plotted the data using bar plots and heatmaps with R statistical software (version 4.2.0) and GraphPad Prism for Windows (GraphPad Software version 8.0.1, California, USA; www.graphpad.com). The Raw RNA-seq data of Mock and IL-27-stimulated FBS-MDMs was stored in GEO, with ascension number GSE262963.

### 2.6 Real-time quantitative PCR

Total RNA was extracted from FBS-MDMs stimulated or not with 25 ng/ml IFN-β, IFN-γ, IFN-λ1, or IL-27 (BioLegend) for 18 h, as reported in section 2.2. Then, 300 ng of RNA was used to construct cDNA libraries for each experimental group using the commercial RevertAid Minus First Strand cDNA Synthesis Kit (Thermo Scientific), following the manufacturer´s protocol. RT-qPCR was used to validate the expression of 14 key DEGs obtained by RNA-Seq using a set of specific primers (**Supplementary Table 1**). PCR amplifications were performed using the Maxima SyBR Green system (Thermo Fisher Scientific). The Bio-Rad CFX manager was used to obtain the cycle thresholds (Ct) determined for each sample using a regression fit in the linear phase of the PCR amplification curve. The relative expression of each target gene was normalized to that of the unstimulated control and housekeeping gene GAPDH (ΔΔCt) and was reported as the Log2FC (**Supplementary Table 2**).

### 2.7 Cytokine quantification

Levels of Tumor necrosis factor-alpha (TNF-α), IL-6, and CCL-2 were quantified in culture supernatants of FBS-MDMs stimulated or not stimulated with 25 ng/ml IFN-β1, IFN-γ, IFN-λ1, or IL-27 (BioLegend) for 18 h, using commercially available ELISA kits (BioLegend) following the manufacturer´s instructions. The detection limit was 2-10 pg/mL.

### 2.8 Transcriptomic analysis of human immune and non-immune cells stimulated with IL-27

To determine whether the antiviral and inflammatory properties of IL-27 signaling are restricted solely to macrophages, we performed a comparative analysis of the transcriptional responses of various human immune and non-immune cell populations exposed to IL-27. For this purpose, we reanalyzed four publicly available microarray datasets from GEO for various cell types stimulated or not stimulated with 100 ng/mL recombinant human IL-27. The first dataset, GSE44732 (44), was generated on immature monocyte-derived dendritic cells (MDDC) (healthy donors; n=3) and stimulated for 48 h. The second dataset, GSE145334 (54), was performed from purified naïve (CD45RA+, CD45RO-, CCR7+) CD4+ and CD8+ T cells (healthy donors; n=3) that were activated *in vitro* (plate bound anti-CD3 and soluble anti-CD28) for 72 hours. The third dataset, GSE143228 (24), was generated on human normal epidermal keratinocytes (HNEK; n=3) stimulated for 18 h. Finally, GSE201555 (55) was performed on primary human astrocytes (fetal brain donors; n=4) stimulated for 18 h. Differential expression of mRNAs in each experimental group was identified using GEO2R. To define the top DEG, we selected genes with an p-val< 0.05 and |Log2 Fold Change (IL-27-treated cells/unstimulated cells) |> 0.6.

### 2.9 CHIKV stock and viral titration

A clinical isolate of CHIKV was obtained following the protocol described in (56). The virus was derived from a chikunguya fiver patient in the acute phase of viral infection (kindly gifted by Professor Francisco Javier Díaz, University of Antioquia). CHIKV was amplified in Vero cells (ATTC CCL-81), as previously reported (26, 45). Briefly, Vero cells were grown in Dulbecco’s Modified Eagle Medium (DMEM; Sigma-Aldrich, St. Louis, USA) supplemented with 5% FBS, 4 mM L-glutamine, 0.3% (v/v) NaCO_3_, and 1% (v/v) Corning™ antibiotic-antimycotic solution. The cells were then incubated at 37°C with 5% CO_2_ until reaching a density of 1x10^5^-1x10^6^ cells/mL. For virus propagation, Vero cells were inoculated with CHIKV at MOI of 0.1 and incubated at 37°C with 5% CO_2_ for 2 days or until an advanced cytopathic effect was observed. Next, supernatants were collected, precleared by centrifugation (1650 x g for 10 min), and stored at -80°C. CHIKV stock and culture supernatants from CHIKV-infected cells were titrated by plaque assay on Vero cells, as previously described (45).

### 2.10 DENV-2 stock and viral titration

DENV-2 New Guinea C was provided by the Centers for Disease Control and Prevention (CDC, USA) and was amplified in *Ae. albopictus* derived C6/36-HT cells (ATCC), as was previously reported (57). Briefly, C6/36-HT cells were cultured in Leibovitz’s L-15 medium (L-15; Sigma-Aldrich) supplemented with 5% FBS and 1% Corning™ antibiotic-antimycotic solution. The cells were incubated at 34°C in cell culture flasks at a density of 1x10^5^-1x10^6^ cells/mL. For DENV-2 propagation, C6/36-HT cells were inoculated with DENV-2 at MOI of 0.1 and incubated at 34°C for 4 days or until an advanced cytopathic effect was observed. Subsequently, supernatants were collected, precleared by centrifugation (1650 x g for 10 min), and stored at -80°C. DENV-2 stock and culture supernatants from DENV-2-infected cells were titrated by plaque assay on BHK-21 cells (ATCC), following the protocol previously described (25, 57). BHK-21 cells were cultured similarly to Vero cells.

### 2.11 *In vitro* antiviral assay

Human FBS-MDMs were pre-treated or not with 25 ng/ml IFN-β, IFN-γ, IFN-λ1, or IL-27 for 18 h at 37°C and 5% CO_2_. Subsequently, the cells were either left uninfected (Mock) or infected with CHIKV or DENV-2 at MOI 5 in serum-free RPMI-1640 medium, as previously reported (26, 45, 47). Culture supernatants were harvested at 24 hpi and stored at -80°C. Viral replication of CHIKV and DENV-2 in FBS-MDMs was evaluated by plaque assay on Vero or BHK-21 cells, respectively. The % inhibition of viral replication was calculated using the equation:


$$\%\;Inhibition=\;\left[1-\frac{\text{PFU}/\text{mL in IFN or IL}-27-\text{stimulated MDMs}}{\text{PFU}/\text{mL in unstimulated}-\text{MDMs}}\right]*100$$

### 2.12 Statistical analysis

Statistical analyses were performed using the GraphPad Prism software for Windows and R statistical software. We evaluated data normality using Shapiro-Wilk test, and One-way ANOVA with Fisher’s LSD post test was performed for data analysis. Data are presented as mean ± SD, Log2 Fold Change (Log2 FC), or TPM. Statistical significance was defined as p< 0.05 (*) (**Supplementary Table 3**).

### 2.13 Illustration of flowcharts and schematic models

The flowcharts and schematic models were created using BioRender (https://www.biorender.com/).

## 3 Results

### 3.1 Morphological and phenotypic characterization of primary human monocyte-derived macrophages

Macrophages play a crucial role in the innate immune response as they are key regulators of the IFN and IL-27 system, orchestrating and enhancing IFN- and IL-27-dependent antiviral responses in both infectious and autoinflammatory diseases (25, 58–60).

**Figure 1:**
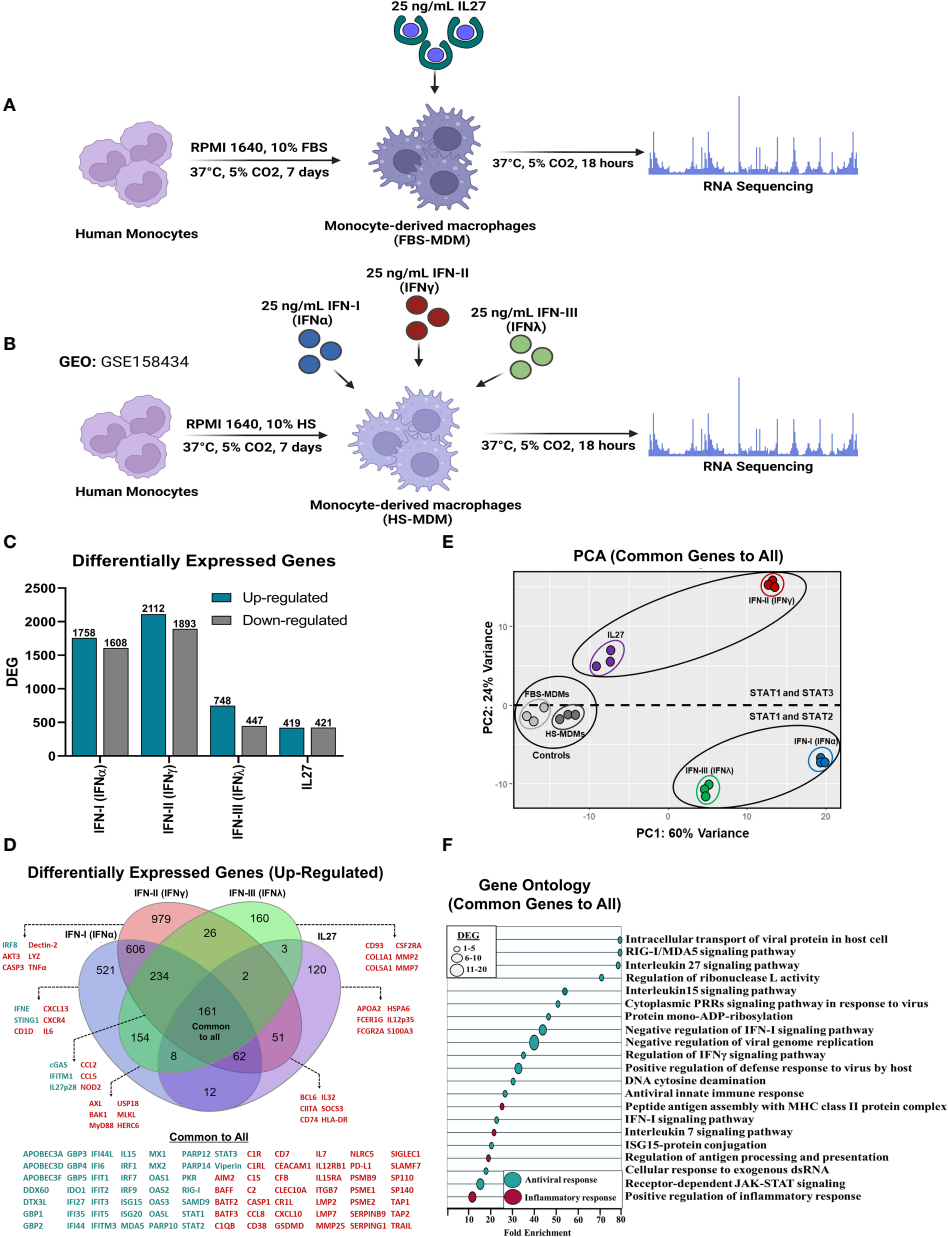
Similar to IFNs, IL27 treatment induces a robust antiviral program in human macrophages. Human primary FBS-MDMs cultures (n= 3) were either stimulated or not with 25 ng/mL recombinant human IL27 for 18 h, and mRNA sequencing was performed using bulk RNA-seq **(A)**. For the publicly available RNA-seq dataset (GSE158434), human HS-MDMs (n= 3) were stimulated with 25 ng/mL of recombinant human IFNα, IFNγ, or IFNλ for 18 h **(B)**. The flowcharts shown in A and B were created using BioRender. Both up- and down-regulated DEG upon IL27 and IFNs treatment **(C)**. Venn diagram indicating upregulated DEG in MDMs after treatment **(D)**; green: antiviral response genes and red, pro-inflammatory, and immune response genes. PCA of common DEG after IL27 and IFNs treatment **(E)**. A GO enrichment analysis based on the common DEG identified by comparing MDMs treated with IL27 and MDMs treated with IFNs is shown **(F)**. DEG were selected from genes with an p-val< 0.05, and |Log2 Fold Change (IL27 or IFN-treated MDMs/unstimulated MDMs) |> 0.6.

In this study, we performed a comparative analysis between two independent RNA-seq datasets. The first dataset comprised MDMs treated with IFNs and was downloaded from the Gene Expression Omnibus (GEO) database under accession number GSE158434 (50). The second RNA-seq dataset was generated from our research, involving FBS-MDM treated with IL-27. Both FBS-MDMs and HS-MDM were acquired following the experimental design outlined in **Figures 1A, B** respectively, as previously reported (26, 45, 50, 57, 61, 62). To validate our FBS-MDMs model, we performed a morphologic and phenotypic characterization. FBS-MDMs have a classical macrophage-like oval or ameboid cell morphology (**Supplementary Figure 1A**), low nuclear-cytoplasmic ratio (**Supplementary Figure 1B**), and high cytoplasmic complexity (**Supplementary Figure 1C**). Moreover, FBS-MDMs have a high cell-surface expression of classic macrophage markers, including CD68/macrosialin (**Supplementary Figure 1D**), and costimulatory molecules such as CD40 and CD86 (**Supplementary Figures 1E, F**, respectively).

Next, we performed a multi-transcriptomic analysis to assess the homogeneity of MDMs following treatment with IFNs or IL-27, aiming to identify the DEGs. First, we normalized gene expression by calculating the transcript per million (TPM) of well-characterized housekeeping genes, since these genes are stable, constitutively expressed reference genes, and are used to normalize the expression of genes of interest in diverse molecular techniques, including RNA-Seq and RT-qPCR (63). In general, the expression levels of housekeeping genes were similar between HS-MDMs and FBS-MDMs unstimulated controls (**Supplementary Figure 2A**), indicating that the samples were comparable. However, it is important to note that the transcriptional expression of certain housekeeping genes, including structural genes such as β-Actin (*ACTβ*) and β-Tubulin (*TUBβ*); and metabolic genes, including glyceraldehyde-3-phosphate dehydrogenase (*GAPDH*) and phosphofructokinase 1 (*PFK1*), was downregulated in IFN-α- and IFN-γ-stimulated HS-MDMs compared to that in unstimulated HS-MDMs (**Supplementary Figure 2A**). This suggests that treatment with IFN-I or IFN-II results in significant metabolic and structural changes in the macrophages.

We also evaluated the RNA-Seq transcriptional profile of macrophage markers to validate our MDMs models and to identify genes that were differentially expressed in response to IFNs and IL-27 treatment. Both FBS-MDMs and HS-MDMs exhibited detectable mRNA levels of *CD14*, *CD16A*, and *CD163*, thus confirming the expression of classic macrophage markers (**Supplementary Figure 2B**). The expression of these macrophage markers was either up- or downregulated, depending on the treatment, compared to untreated samples. Furthermore, the mRNA level of *M-CSF* was significantly decreased in HS-MDMs after treatment with IFN-I and II compared to untreated cells, whereas in FBS-MDMs, the expression level was not altered after IL-27 treatment (**Supplementary Figure 2B**).

To assess the capability of MDMs to respond to treatment with different types of IFNs or IL-27, transcriptomic analysis focused on the expression of IFN- and IL-27-receptors (**Supplementary Figure 2C**). We found that FBS-MDMs and HS-MDMs expressed all IFNR and IL-27R, albeit with varying expression levels depending on the treatment. Importantly, IFNs or IL-27 treatment modulated the expression levels of these receptors (**Supplementary Figure 2C**). While *IFNLR1* and *IL6ST* were significantly upregulated after IFN-I-stimulation, both *IFNAR2* and *IL6ST* were significantly upregulated whereas *IL27Rα* was downregulated following IFN-II treatment of HS-MDMs. All IFN-stimulated HS-MDMs and IL-27-stimulated FBS-MDMs showed *IFNGR1* downregulation. Overall, these results indicate that the RNA-Seq datasets are comparable, as evidenced by similar mRNA expression levels of housekeeping genes, macrophage markers, and IFN/IL-27 receptors in both treated HS-MDMs and FBS-MDMs compared with unstimulated controls.

### 3.2 Interferons and interleukin 27 treatment induces a robust pro-inflammatory and antiviral program in human macrophages

To define the top DEGs, we selected genes with an p-val< 0.05 and |Log2 FC (IFN or IL-27-stimulated MDMs/Unstimulated MDMs) | > 0.6 (**Figure 1C**). A total of 1758 and 1608 genes were upregulated and downregulated, respectively, by IFN-α. IFN-γ upregulated 2112 genes and downregulated 1893 genes, while IFN-λ upregulated 748 genes and downregulated 447 genes. Finally, 840 DEGs were identified in IL-27-treated MDMs, with 419 being promoted and 421 being suppressed. These findings indicate a higher increase in DEG by type I and II IFNs compared to IFN-III and IL-27, suggesting a more complex response.

Although the number of DEG varied according to the treatment, we performed a comparative analysis focusing only on the DEG upregulated by IFNs or IL-27 (**Figure 1D**). We found that IFNs and IL-27 induced the expression of 161 common DEG, including a high diversity of AVPs/ISGs, JAK-STAT signaling components, and pro-inflammatory factors, such as complement system molecules, cytokines, and CC- and CXC-motif chemokines. However, it is also important to highlight that each treatment group had a certain proportion of unique DEG, suggesting that different treatments may trigger distinct immune responses (**Figure 1D**). To determine the differences in the transcriptional expression levels of common DEG induced by IFNs and IL-27 in human MDMs, we performed principal component analysis (PCA). PCA showed high heterogeneity in the mRNA profiles, with approximately 84% of the variance (PC1 = 60%; PC2 = 24%) (**Figure 1E**). It was able to discriminate unstimulated MDMs from IFNs- and IL-27-stimulated MDMs, but not between untreated FBS-MDMs and HS-MDMs. Moreover, PC1 showed the intensity of the transcriptional profile induced by IFNs and IL-27, suggesting that all types of IFNs and IL-27 induce different transcript levels of common DEGs in human MDMs. Additionally, PC2 could show the differences between the signaling pathway triggered by IFNs and IL-27, showing a group conformed by IFN-α and IFN-λ (STAT-1/STAT-2/IRF-9 dependent), and other group conformed by IFN-γ and IL-27 (STAT-1 and STAT-3 dependent), suggesting that IFN-I/IFN-III or IFN-II/IL-27 induced similar transcriptional profile in MDMs.

Furthermore, Gene Ontology (GO) analysis of common DEG revealed their association with the induction of a robust antiviral program (**Figure 1F**). The GO terms were significantly enriched in antiviral pathways in MDMs treated with IFNs or IL-27, including intracellular transport of viral proteins in host cells, RIG-I/MDA5 signaling pathway, regulation of ribonuclease L activity, cytoplasmic PRRs signaling pathway in response to virus, 15 signaling pathways, negative regulation of viral genome replication, positive regulation of defense response to virus by host, DNA cytosine deamination, antiviral innate immune response, ISG15-protein conjugation, cellular response to exogenous dsRNA, and receptor-dependent JAK-STAT signaling. These results indicate that IFNs and IL-27 induce the establishment of a common antiviral program that is dependent on AVPs/ISGs expression in human primary MDMs (**Figure 1D**). GO analysis also showed that common DEG were associated with the induction of a robust pro-inflammatory program (**Figure 1F**). The most significantly enriched biological processes were interleukins 7, peptide antigen assembly with MHC class II protein complex, regulation of antigen processing and presentation, and positive regulation of the inflammatory response. In summary, the results demonstrate that IFNs and IL-27 induce a common pro-inflammatory and antiviral program that is dependent on AVPs/ISGs expression in human MDMs.

### 3.3 Interferons and interleukin 27 modulated the expression of JAK-STAT signaling components and induced the establishment of an antiviral state in human macrophages

**Figure 2:**
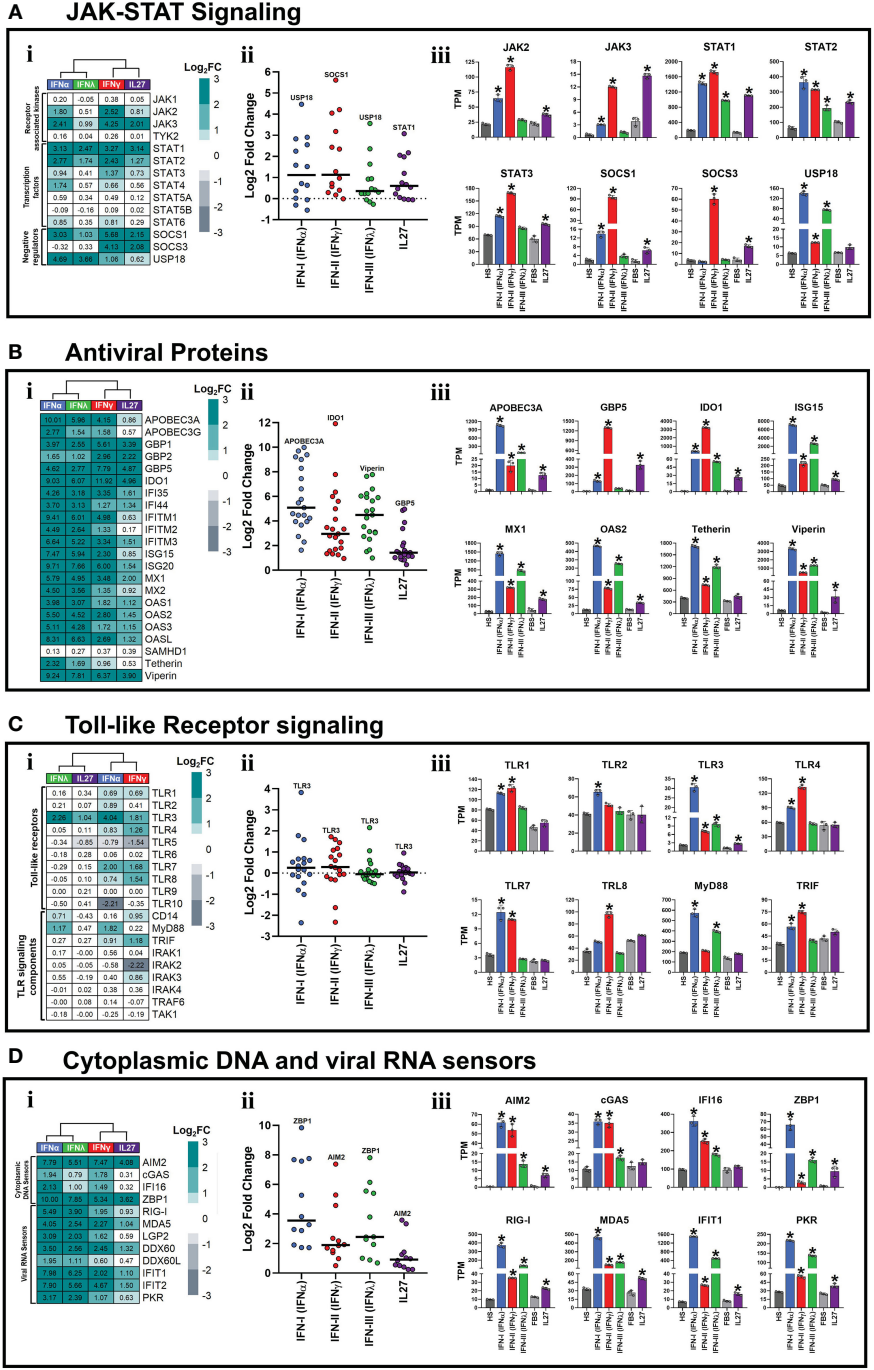
Interferons and Interleukin 27 modulated the expression of JAK-STAT signaling components and induced the establishment of an antiviral state in human macrophages. Human MDMs (n= 3) were stimulated or not with IFNs, or IL-27 as shown in **Figures 1A, B**. Gene expression (mRNA) is expressed as Log2FC heatmaps (i), median expression intensity of signaling axis components (ii), and transcripts per million (TPM) (iii). mRNA expression of JAK-STAT signaling components **(A)**, antiviral proteins **(B)**, Toll-like receptor signaling components **(C)**, and cytoplasmic viral DNA and RNA sensors **(D)** in IFN- and IL27-stimulated MDMs. TPM are presented as mean ± SD, data normality was evaluated using Shapiro-Wilk test, and a one-way ANOVA with Fisher’s LSD post-test was performed. Significant differences between unstimulated HS-MDMs and IFN-stimulated HS-MDMs, or unstimulated FBS-MDMs and IL27-stimulated FBS-MDMs were defined as p<0.05 (*). In (ii) every point corresponds to the mean gene expression (n= 3) for the specific signaling axis components reported in (i).

Since the main feature of IFNs is their ability to activate JAK-STAT signaling and induce the expression of AVPs/ISGs to establish an antiviral state (2–6), we next determined whether IL27 also has the ability to activate a similar biological process. As shown in **Figure 2A**, all three types of IFNs and IL27 modulated the expression of JAK-STAT signaling components, including JAK kinases, STAT transcription factors, and negative regulators, to varying degrees. As observed in the PCA (**Figure 1E**), IFNα/IFNλ or IFNγ/IL27 had similar patterns of JAK-STAT signaling component expression (**Figure 2A**i). Notably, the median intensity of JAK-STAT signaling component expression was higher for IFNα and IFNγ than for IFNλ and IL27 (**Figure 2A**ii). However, like IFNα- and IFNγ-stimulated HS-MDMs, IL27-stimulated FBS-MDMs up-regulated the mRNA expression of JAK2 and JAK3 kinases compared to their respective unstimulated controls (**Figure 2A**iii). Furthermore, while IFNγ induces higher mRNA expression of JAK3, a recognized target gene of STAT3 (64), it is highly induced by both IFNγ and IL27. All three types of IFNs and IL27 induce significant mRNA expression of STAT transcription factors, including STAT1, STAT2, and STAT3 (**Figure 2A**iii). In terms of negative regulators of JAK-STAT signaling, we observed that, like IFNα and IFNγ, IL27 upregulated SOCS1 mRNA expression compared to their respective unstimulated controls (**Figure 2A**iii). SOCS3, the main negative regulator of IL27 signaling (65), was induced by IFNγ and IL27, whereas USP18, the main negative regulator of IFN-I and IFN-III signaling (66), was the higher JAK-STAT signaling component induced by these two types of IFNs (**Figures 2A**ii,iii), suggesting that the induction of negative regulators of JAK-STAT signaling is differentially regulated and dependent on the type of IFN or IL27 involved in the activation of JAK-STAT signaling in human MDMs.

IFN-I and IFN-III have been classically associated with the induction of a robust antiviral program that is dependent on the expression of AVP/ISGs involved in establishing of antiviral state in host cells (16). In contrast, IFN-II has been classically associated with macrophage activation, induction of the inflammatory response, and promotion of antigen presentation (67). Based on these biological functions, we proceeded to determine whether both IFNs and IL-27 promoted the expression of AVPs/ISGs. Although the level of expression varied depending on the treatment, we observed that all types of IFNs and IL-27 significantly upregulated the mRNA expression of many AVPs/ISGs in human MDMs (**Figure 2B**i). As observed in the PCA (**Figure 1E**), IFN-α/IFN-λ or IFN-γ/IL-27 had similar patterns of ISGs/AVPs expression (**Figure 2B**i). However, type I IFN most strongly promoted AVPs/ISGs expression, followed by IFN-III, IFN-II, and lastly, IL-27 induced the lowest mRNA levels of AVPs/ISGs (**Figure 2B**ii). Additionally, except for *Tetherin*, which was not induced in response to IL-27 treatment, both IFNs and IL-27 promoted significant expression of AVPs/ISGs, including *APOBEC3A*, *GBP5*, *IDO1*, *ISG15*, *MX1*, *OAS2*, and *Viperin* (**Figure 2B**iii). These results suggest that IFN-I and IFN-III induce stronger antiviral responses than IFN-II and IL-27. However, IFN-γ and IL-27 expressed higher mRNA levels of *GBP5* (**Figure 2B**iii) than IFN-α and IFN-λ. In summary, the results suggest IFNs and IL-27 activate the JAK-STAT signaling pathway (**Figure 2A**) and induce significant expression of AVPs/ISGs involved in establishing an antiviral state in human MDMs.

### 3.4 Interferons and interleukin 27 modulated the expression of pattern-recognition receptors in human macrophages

TLRs work as key regulators of inflammatory signals, which, after binding with specific PAMPs or DAMPs, signal through IRFs and the NF-κB pathway (41, 68, 69). Although it has been well described that IFNs modulate PRRs expression to amplify the antiviral response (70), the role of IL-27 in the induction of PRRs expression is not well known.

As reported in **Figure 2C**i, a distinctive gene expression profile was observed in the IFN-stimulated HS-MDM or IL-27-stimulated FBS-MDM, compared with their unstimulated controls. However, IFN-α and IFN-γ induced the strongest modulation of TLRs and their signaling components compared to IFN-λ and IL-27 (**Figure 2C**ii). IFN-α promoted the expression of *TLR1*, *2*, *3*, *4*, *7*, and *8*, in addition to *MyD88* and *TRIF* (**Figure 2C**iii). IFN-γ induced the expression of *TLR1*, *3*, *4*, *7*, and *8*, as well as *TRIF* (**Figure 2C**iii). IFN-λ induced the expression of *TLR3* and *MyD88*, whereas *IL-27* induced only the expression of *TLR3* (**Figure 2C**iii), suggesting that IFN-I and IFN-II promote a higher TLR-dependent inflammatory and antimicrobial state than IFN-III and IL-27, in human MDMs.

We observed that mRNA expression levels varied depending on the treatment; however, both IFNs and IL-27 significantly up-regulated the expression of numerous cytoplasmic viral DNA and RNA sensors in human MDMs (**Figure 2D**i). As observed in the PCA (**Figure 1E**), IFN-α/IFN-λ or IFN-γ/IL-27 had similar patterns of these PRRs expression (**Figure 2D-i**). However, IFN-α exhibited the most significant promotion of cytoplasmic viral DNA and RNA sensor expression, followed by IFN-λ, IFN-γ, and IL-27 induced the lowest mRNA levels (**Figure 2D**ii). We found a significant upregulation of a wide range of viral DNA sensors, including *AIM2*, *cGAS*, *IFI16*, and *ZBP1*, by all three types of IFNs (**Figure 2D**iii). At lower levels, IL-27 also induced the expression of *AIM2* and *ZBP1* (**Figure 2D**iii). When examining viral RNA sensors, we found that IFNs and IL-27 significantly upregulated the mRNA expression of RIG-like receptors, including the key players in SARS-CoV-2 pathogenesis (68), *RIG-I* and *MDA5* long with other viral RNA sensors such as *IFIT1* and *PKR* (**Figure 2D**iii). Notably, IFN-α- and IFN-λ-stimulated HS-MDM exhibited higher mRNA expression of all viral RNA sensors than IFN-γ-stimulated HS-MDMs and IL-27-stimulated FBS-MDMs, suggesting that IFN-α and IFN-λ might elicit a stronger antiviral response against RNA viruses than IFN-γ and IL-27. In addition, our results indicate that while all types of IFNs and IL-27 modulate the expression of various types of PRRs, IFN-I and IFN-II induce more significant upregulation of TLRs and cytoplasmic viral DNA sensors in human primary MDMs compared to IFN-III and IL-27.

### 3.5 Type I and II interferons are the most potent inducers of STAT1-dependent pro-inflammatory state compared with IFN-III and IL-27, in human macrophages

**Figure 3:**
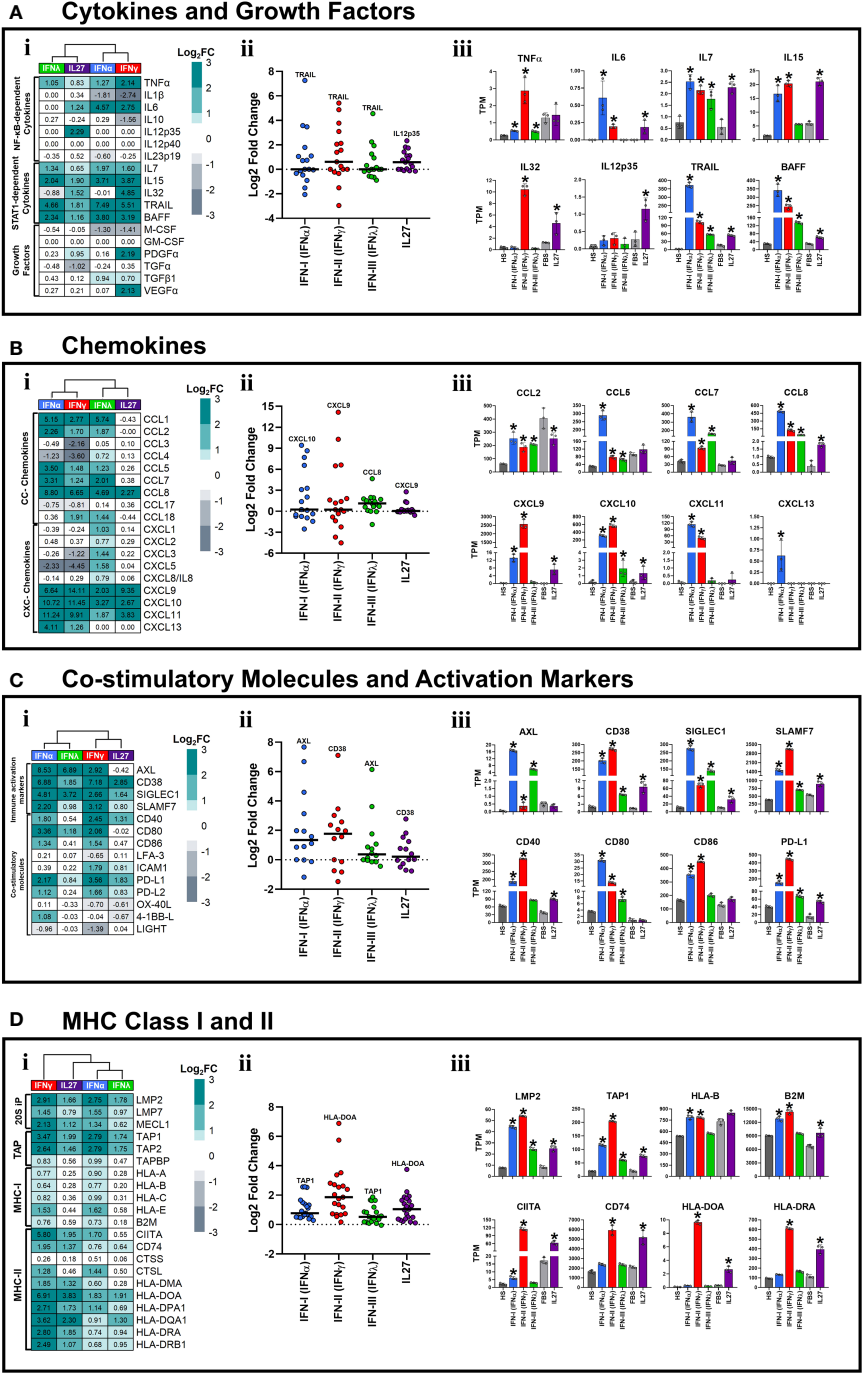
Type I and II interferons induced a robust pro-inflammatory state in human macrophages. Human MDMs (n= 3) were stimulated or not with IFNs, or IL-27 as shown in **Figures 1A, B**. Gene expression (mRNA) is expressed as Log2FC heatmaps (i), median expression intensity of signaling axis components (ii), and transcripts per million (TPM) (iii). mRNA expression of cytokines and growth factors **(A)**, CC- and CXC-motive chemokines **(B)**, costimulatory and immune activation markers **(C)**, and MHC class I and II **(D)** in IFN- and IL27-stimulated MDMs. TPM are presented as the mean ± SD, data normality was evaluated using Shapiro-Wilk test, and One-way ANOVA with Fisher’s LSD post-test was performed. Significant results between unstimulated HS-MDMs and IFN-stimulated HS-MDMs, or unstimulated FBS-MDMs and IL27-stimulated FBS-MDMs are defined as p<0.05 (*). In (ii) every point corresponds to the mean gene expression (n= 3) for the specific signaling axis components reported in (i).

Although it is less frequently reported, IFNs also help to regulate the activity of the immune system through the induction of pro-inflammatory factors, including cytokines and chemokines (7–10). These factors mediate the recruitment and activation of different immune cell populations at the infection site. Moreover, we recently reported that treatment of THP-1-derived macrophages with IL-27 induces high and significant expression of STAT-1-dependent cytokines and chemokines in an IFN-independent manner (26). Therefore, to determine whether IFNs and IL-27 induce a pro-inflammatory response in human primary macrophages, we evaluated the transcriptional response of IFN-stimulated HS-MDMs or IL-27-stimulated FBS-MDMs, compared with their respective controls. We observed a distinct or treatment-dependent gene expression profile (**Figure 3A**i). Notably, the median intensity of cytokine expression was higher for IFN-γ and IL-27 than for IFN-α and IFN-λ (**Figure 3A**ii). In addition, our findings revealed that while IFNs upregulated the expression of *TNFα*; IFN-α, IFN-γ, and IL-27 promoted the expression of *IL6* (**Figure 3A**iii). Furthermore, *IL7*, *IL15*, TNF-related apoptosis-inducing ligand (*TRAIL*), and B-cell activating factor (*BAFF*) levels were significantly increased in MDMs stimulated with each type of IFN or IL-27 (**Figure 3A**iii). IFN-γ and IL-27 significantly induced the expression of *IL32*, whereas *IL12p35* was upregulated by IL-27 only (**Figure 3A**iii).

Next, we sought to determine whether CC- and CXC-motif chemokine expression was altered in MDMs stimulated with IFNs or IL-27. As reported in **Figure 3B**i, a distinctive gene expression profile was observed in IFN-stimulated HS-MDM or IL-27-stimulated FBS-MDM compared to their respective unstimulated controls. However, IFN-α and IFN-γ induced the strongest of CC- and CXC-motive chemokines expression compared with IFN-λ and IL-27 (**Figure 3B**ii). For instance, we observed that IFNs significantly increased the mRNA expression of CC-motif chemokines, including *CCL2*, *CCL5*, *CCL7*, and *CCL8*. In contrast, IL-27 promoted the expression of *CCL8* but downregulated that of *CCL2* compared to that in the unstimulated control (**Figure 3B**iii). IFN-I was the most potent inducer of chemokine expression. Regarding CXC-motif chemokines, only *CXCL10* was induced by both IFNs and IL-27, with type I and II IFNs being the strongest inducers (**Figure 3B**iii). *CXCL9* was induced by type I and II IFNs and IL-27, whereas *CXCL11* was exclusively induced by IFN-I and II (**Figure 3B**iii). Interestingly, only IFN-I upregulated *CXCL13* expression (**Figure 3B**iii). Together, these results suggest that IFNs and IL-27 induce a robust STAT-1-dependent pro-inflammatory response in human MDMs, with IFN-I and II being the most potent inducers compared with IFN-III and IL-27.

### 3.6 Interferons and IL-27 induce macrophage activation and promote adaptive immune response by modulating cell-surface-expressed activation markers, co-stimulatory molecules, and MHC-I/II genes

The best characterized function of IFN-γ is its capability to induce macrophage activation and promote antigen presentation to T cells by upregulating co-stimulatory molecules, MHC-I and MHC-II proteins (11–13). However, the ability of other types of IFN and IL-27 to induce macrophage activation and promote antigen presentation is poorly understood.

Although the expression levels varied with treatment, both IFNs and IL-27 significantly upregulated the mRNA expression of many cell-surface-expressed immune activation markers in human MDMs (**Figure 3C**i). As observed in PCA (**Figure 1E**), IFN-α/IFN-λ and IFN-γ/IL-27 exhibited similar patterns of these genes (**Figure 3C**i). However, type I and II IFNs most strongly promoted cell-surface-expressed immune activation markers expression, followed by IFN-III and IL-27 (**Figure 3C**ii). Furthermore, we observed that IFNs and IL-27 upregulated the mRNA expression of *CD38*, sialic acid binding igg-like lectin 1 (*SIGLEC1*), and SLAM Family Member 7 (*SLAMF7*) in human MDMs (**Figure 3C**iii). However, IFN-γ and IFN-α induced higher mRNA expression of *CD38* and *SLAMF7* than IFN-λ and IL-27. Furthermore, IFNs induced high and significant expression of AXL Receptor Tyrosine Kinase (*AXL*) mRNA compared to unstimulated HS-MDMs (**Figure 3C**iii).

Among the co-stimulatory molecules, IFN-α and IFN-γ significantly increased the mRNA expression of *CD40*, *CD80*, *CD86*, and Programmed Cell Death 1 Ligand (*PD-L1*) compared to that in the control. IL-27, on the other hand, promoted a low but significant expression of *CD40* and *PD-L1* (**Figure 3C**iii). The latter was the only gene induced by both IFNs and IL-27.

Next, we assessed the expression of MHC-I/II molecules in MDMs stimulated with IFNs and IL-27. As reported in **Figure 3D**i, a distinctive gene expression profile was observed in IFNs- and IL-27-stimulated MDMs compared to their unstimulated controls. However, IFN-γ and IL-27 induced the strongest induction of MHC-I/II molecules compared with IFN-α and IFN-λ (**Figure 3D**ii). In addition, we observed that IFNs and IL-27 significantly upregulated the mRNA expression of 20S immunoproteasome (20S-IP) components, including low molecular mass protein 2 (*LMP2*), and MHC-encoded peptide transporter (TAP) components, including *TAP1* (**Figure 3D**iii). However, only IFN-α and IFN-γ significantly upregulated the expression of MHC-I molecules, including *HLA-B* and Beta-2-Microglobulin (*B2M*) mRNA, whereas IL-27 induced the expression of *B2M* only, compared to their respective unstimulated controls (**Figure 3D**iii). Among MHC-II genes, only IFN-γ and IL-27 significantly upregulated the mRNA expression of class II trans-activator (*CIITA*), *CD74*, *HLA-DOA*, and *HLA-DRA* (**Figure 3D**iii), whereas IFN-α only induced the expression of *CIITA*. These results suggest that both IFN-II and IL-27 may enhance the antigen presentation capacity of MDMs to CD4+T cells. In summary, IFNs and IL-27 induced macrophage activation and promotes adaptive immune response through the modulation of cell-surface-expressed immune activation markers, co-stimulatory molecules, and/or MHC-I/II genes.

### 3.7 Pro-inflammatory and antiviral properties of interleukin 27 are independent of the interferon induction in human macrophages

**Figure 4:**
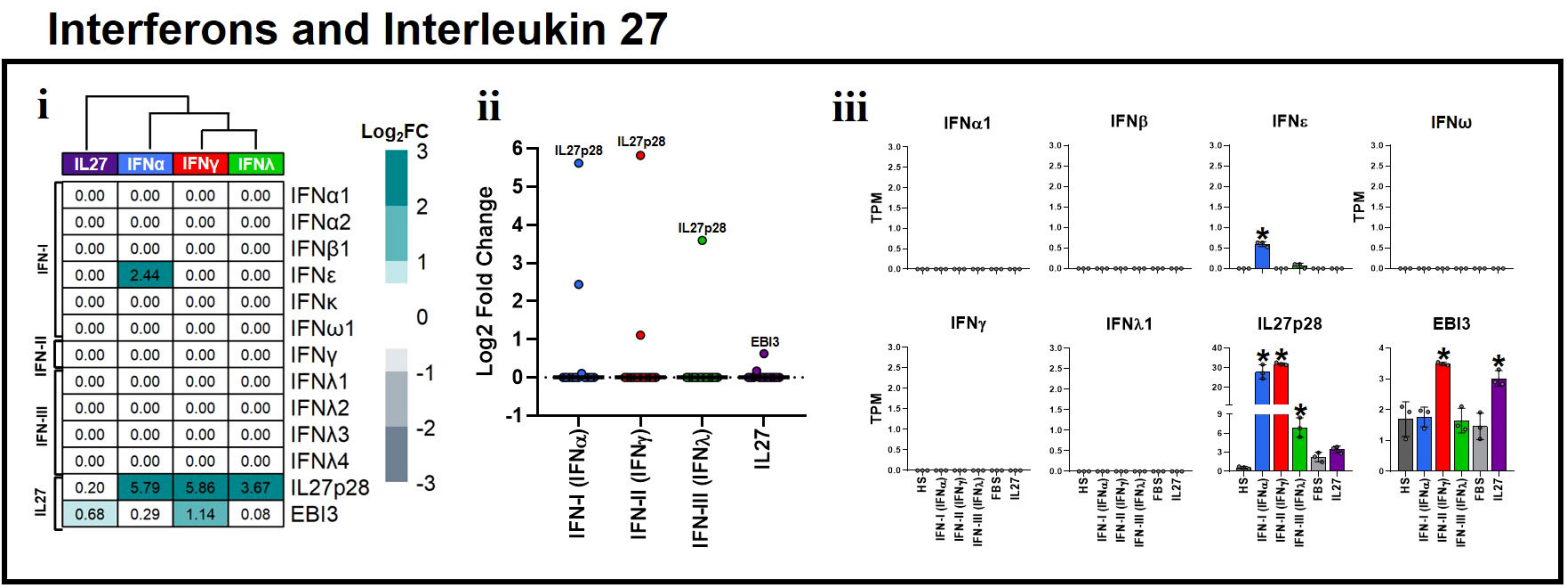
Proinflammatory and antiviral properties of Interleukin 27 are independent of interferon induction in human macrophages. Human MDMs (n= 3) were stimulated or not with IFNs, or IL-27 as shown in **Figures 1A, B**. Gene expression (mRNA) of IFN-I, IFN-II, IFN-III, and IL27 subunits is expressed as Log2FC heatmaps (i), median expression intensity of signaling axis components (ii), and transcripts per million (TPM) (iii). TPM are presented as the mean ± SD, data normality was evaluated using Shapiro-Wilk test, and One-way ANOVA with Fisher’s LSD post-test was performed. Significant results between unstimulated HS-MDMs and IFN-stimulated HS-MDMs, or unstimulated FBS-MDMs and IL27-stimulated FBS-MDMs are defined as p<0.05 (*). In (ii) every point corresponds to the mean gene expression (n= 3) for the specific signaling axis components reported in (i).

*In vitro* and *in vivo* evidence have demonstrated that IL-27 directly enhances IFN production in response to viral infections in various cell types (34, 35, 44, 71, 72). Therefore, the potent antiviral activity associated with IL-27 has been attributed to its increased IFN production, which has various antiviral effects. Nevertheless, the ability of IL-27 to induce the production of IFNs is unknown. Hence, to determine whether IFNs or IL-27 promote their own expression or that of another, we evaluated the transcriptional response of MDMs stimulated with IFN-α, IFN-γ, IFN-λ, or IL-27, and compared them with unstimulated controls. Interestingly, we found that neither IFNs nor IL-27 possessed the ability to induce the expression of itself, or the other types of IFN, except for IFN-α, which upregulated the expression of *IFNϵ* mRNA (**Figure 4**i). However, we found that IFNs, but not IL-27, significantly upregulated *IL27p28* mRNA expression, whereas IFN-γ and IL-27 significantly upregulated *EBI3* mRNA expression (**Figures 4**ii,iii). Overall, these results suggest that the antiviral and pro-inflammatory properties of IL-27 are IFN-independent. Moreover, IFNs induce a positive feedback loop on the antiviral response through the induction of IL27p28 gene expression in human MDMs, demonstrating that IL27p28 is an inductor of the antiviral state and an ISG by itself.

### 3.8 Experimental validation of RNA-Seq data by RT-qPCR, ELISA, and flow cytometry

**Figure 5:**
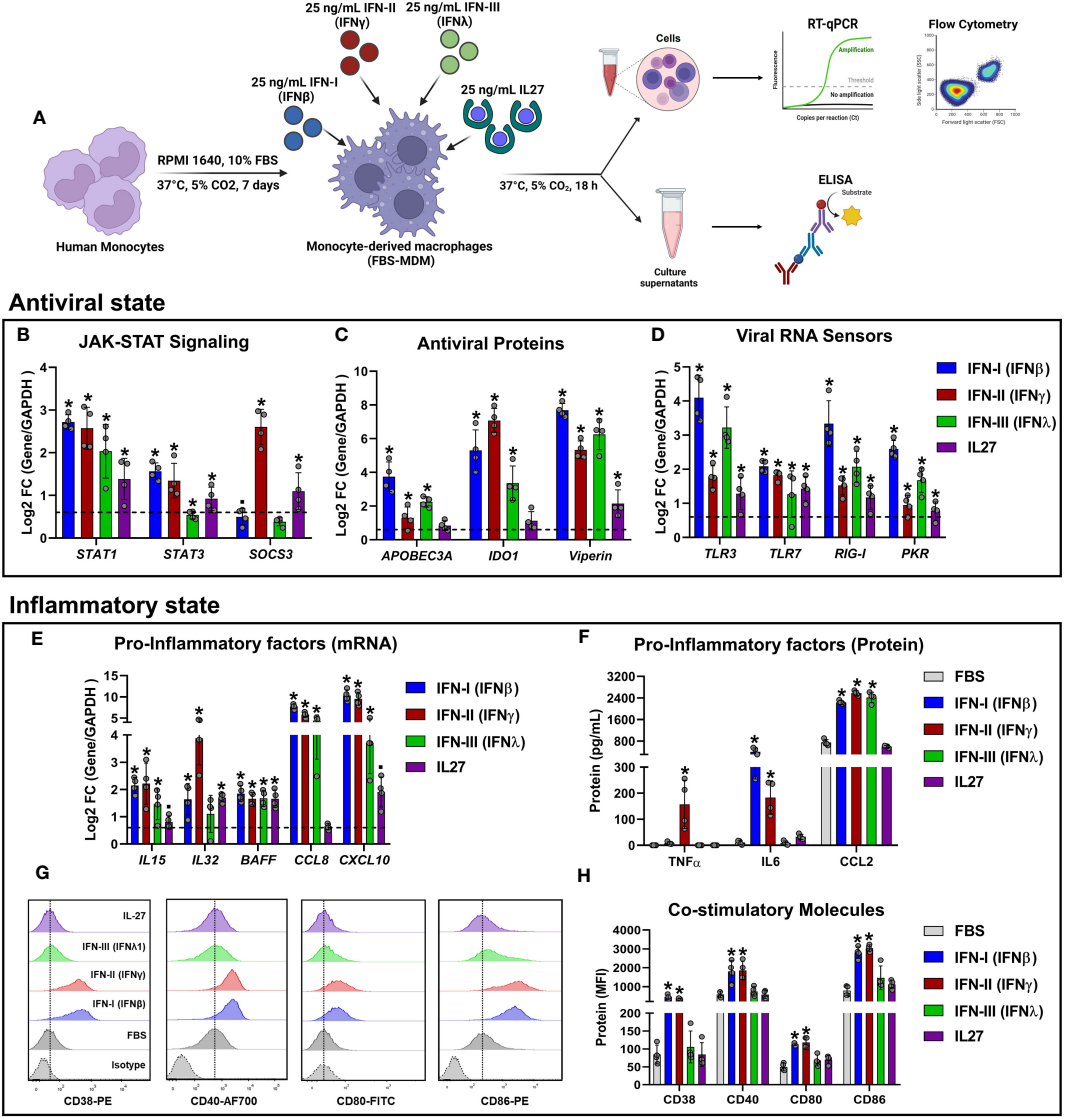
Experimental validation of RNA-Seq results using RT-qPCR, ELISA, and flow cytometry. Human primary FBS-MDM cultures (n= 4) were stimulated or not with 25 ng/mL recombinant human IFN-I (IFN-β), IFN-II (IFN-γ), IFN-III (IFN-λ), or IL-27. Total RNA, culture supernatants, and cells were obtained after 18 h, and RT-qPCR, ELISA, and flow cytometry were performed. The study flowchart shown in **(A)** was created using BioRender. mRNA expression of JAK-STAT signaling components **(B)**, antiviral proteins **(C)**, viral RNA sensors **(D)**, and pro-inflammatory factors **(E)** was analyzed by RT-qPCR. TNF-α, IL-6, and CCL-2 accumulation in the culture supernatants of FBS-MDMs was analyzed by ELISA **(F)**. Histograms **(G)** and MFI **(H)** of CD38, CD40, CD80, and CD86 on the cell surface of FBS-MDM cultures was analyzed by flow cytometry. Data are presented as mean ± standard deviation (SD), data normality was evaluated using Shapiro-Wilk test, and One-way ANOVA with Fisher’s LSD analysis post-test was performed. Significant differences between unstimulated FBS-MDMs and IFN- or IL27-stimulated FBS-MDMs were defined as p<0.05 (*).

To validate our RNA-Seq data, mRNA expression and protein production of the selected DEGs identified by RNA-Seq were measured by RT-qPCR, ELISA, and/or flow cytometry (**Figure 5A**). RT-qPCR results were aligned with RNA-Seq analysis (**Figure 2A**). Both IFNs and IL-27 significantly upregulated *STAT1* mRNA expression. However, while *STAT3* mRNA was significantly upregulated by IFNs and IL-27, *SOCS3* mRNA expression was significantly upregulated by IFN-γ and IL-27 (**Figure 5B**).

Regarding the AVPs encoded by ISGs, we found that both IFNs and IL-27 upregulated *APOBEC3A* and IDO1 mRNA expression (**Figure 5C**). However, these changes in mRNA expression were significant only for IFNs. Additionally, both IFNs and IL-27 significantly upregulated *Viperin* mRNA expression (**Figure 5C**), which was consistent with the findings from the RNA-seq analysis (**Figure 2B**). Next, we examined the expression profile of representative viral RNA sensors (**Figure 5D**), and found that *TLR3*, *RIG-I*, and *PKR* mRNAs were significantly induced by IFNs and IL-27, which was consistent with the findings from the RNA-seq analysis (**Figures 2C, D**). However, unlike RNA-seq data, all types IFN and IL-27 upregulated *TLR7* expression in FBS-MDMs (**Figure 5D**).

To confirm the inflammatory properties of IFNs and/or IL-27 signaling in MDMs, we quantified the expression of STAT-1-dependent pro-inflammatory factors in FBS-MDMs stimulated with either IFNs or IL-27. As expected, IFNs and IL-27 increased *IL15*, *CCL8*, and *CXCL10* mRNA expression (Log2FC >0.6) (**Figure 5E**). However, these changes in mRNA expression were significant only for IFNs, unlike as determined by RNA-Seq analysis (**Figures 3A, B**). However, *BAFF* mRNA expression was significantly upregulated by IFNs and IL-27 treatment (**Figure 5E**). Furthermore, as we found in the RNA-Seq analysis (**Figure 3A**), IFN-γ and IL-27 induced the higher *IL32* mRNA expression in MDMs (**Figure 5E**).

Next, we performed ELISA to quantify the production of TNF-α, IL-6, and CCL-2, which were identified by RNA-Seq analysis in MDMs treated with IFNs or IL-27 (**Figure 3A**). We found that only IFN-γ significantly upregulated TNF-α protein production, whereas IL-6 production was induced by IFN-β, and IFN-γ, compared to unstimulated MDMs (**Figure 5F**). Additionally, as was found by RNA-seq analysis (**Figure 3B**), all types of IFN, but not IL-27, significantly increased CCL-2 production by MDMs (**Figure 5F**).

Finally, we used flow cytometry to evaluate the expression of cell-surface costimulatory molecules following IFNs or IL-27 treatment. Unlike the findings from RNA-Seq analysis (**Figure 3C**), we found that only IFN-I and II significantly upregulated the protein levels of CD38, CD40, CD80, and CD86 on the cell surface of MDMs compared to IFN-III, and IL-27-stimulated MDMs (**Figures 5G, H**). The discrepancy between the mRNA/protein levels of these costimulatory molecules could be associated with their low mRNA induction in response to IFN-III and IL-27 (**Figure 3D**). In general, the experimental validation carried out by RT-qPCR, ELISA, and flow cytometry supported our RNA-Seq results and confirmed that treatment with IFNs or IL-27 induce robust inflammatory and antiviral states in human MDMs, with IFN-I and II being the most potent inducers of inflammatory factors as compared with IFN-III and IL-27.

### 3.9 Pro-inflammatory and antiviral properties of interleukin 27 signaling are independent of cellular lineage

**Figure 6:**
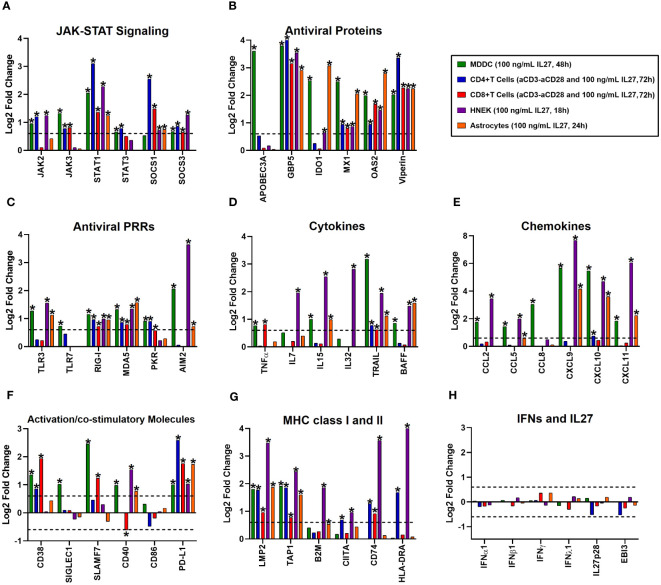
The proinflammatory and antiviral properties of Interleukin 27 signaling are independent of the cellular lineage. A comparative analysis of four publicly available microarray datasets from GEO of different cell populations treated with 100 ng/mL recombinant human IL27 at different time points was performed. It includes immune cell populations, such as monocyte-derived dendritic cells (MDDC, n= 3), naïve CD4+ or CD8+ T cells (n= 3), and non-immune cell populations, including human normal epidermal keratinocytes (HNEK, n= 3), and astrocytes (n= 4). Gene expression (mRNA) was expressed as Log2FC. The mRNA expression of various categories is as follows: JAK-STAT signaling components **(A)**, antiviral proteins/ISGs **(B)**, Antiviral PRRs **(C)**, cytokines **(D)**, chemokines **(E)**, immune activation and co-stimulatory molecules **(F)**, MHC class I and II **(G)**, and IFN/IL27 subunits **(H)**. DEG were selected from genes with an FDR< 0.05, and |Log2 Fold Change (IL27-treated cells/unstimulated cells) |> 0.6.

To investigate whether the pro-inflammatory and antiviral properties of IL-27 signaling are specific to macrophages, we performed a comparative analysis of the transcriptional profiles of various immune cell populations (MDDC, naïve CD4+, or CD8+ T cells), and non-immune cell populations (HNEK and astrocytes) treated with IL-27. This involved reanalyzing four publicly available microarray datasets from GEO, as described in the Materials and Methods section (Section 2.8). As was observed in IL-27-stimulated FBS-MDMs (**Figure 2A**), microarray analysis of different immune and non-immune cells stimulated with IL-27 showed significant upregulation of mRNA expression of different components of JAK-STAT signaling, including *STAT1*, *SOCS1*, and *SOCS3* in the different cell populations (**Figure 6A**); however, some of them were regulated in a cell lineage-dependent manner (for example, *JAK2*, *JAK3*, and *STAT3*). Moreover, treatment with IL-27 induced high and significant expression of AVPs/ISGs (**Figure 6B**) and PRRs (**Figure 6C**), such as *APOBEC3A*, *IDO1*, *TLR3*, *TLR7*, *PKR*, and *AIM2*, in a cell lineage-dependent manner. However, *GBP5*, *MX1*, *OAS2*, and *Viperin* were expressed in all the cell types. Together, these results indicate that IL-27 induces the activation of JAK-STAT signaling and promotes the establishment of an antiviral state in human cells independent of cellular lineage.

Next, we analyzed the expression of cell-specific pro-inflammatory genes following IL-27 stimulation. As was found in IL-27-stimulated FBS-MDMs (**Figure 3**), treatment with IL-27 significantly upregulated the mRNA expression of STAT1-dependend cytokines (**Figure 6D**) and CC- and CXC-motif chemokines (**Figure 6E**) in human MDDC, HNEK, and astrocytes. However, IL-27-stimulated T cells did not exhibit significant expression of pro-inflammatory genes, except *TNFα* in CD8+ T cells, *CXCL10* in CD4+ T cells, and *TRAIL* in both CD4+ and CD8+ T cells. Moreover, we found that IL-27 significantly increases the expression of cell-surface-expressed immune activation markers and costimulatory molecules in a cell-lineage-dependent manner (**Figure 6F**). MHC class I and II genes, including *LMP2* and *TAP1*, were induced in both immune and non-immune cells, whereas *B2M*, *CIITA*, *CD74*, and *HLA-DRA* were induced in a cell-lineage-dependent manner (**Figure 6G**). Together, these results suggest that pro-inflammatory properties of IL-27 are partially dependent on cellular lineage.

Finally, we evaluated the ability of IL-27 to induce the expression of IFNs in different immune and nonimmune cell populations. As we reported in IL-27-stimulated FBS-MDMs (**Figure 4**), we confirmed that IL-27 by itself did not induce significant expression of any type of IFN or IL-27 subunit in the different cell populations (**Figure 6H**), confirming that pro-inflammatory and antiviral properties of IL-27 signaling are independent of IFNs production. Altogether, these results demonstrate that like IFNs, IL-27 activates JAK-STAT signaling and induces a robust pro-inflammatory and antiviral response in human cells, independent of cellular lineage.

### 3.10 Interferons and interleukin 27 interfered with the replication of chikungunya and dengue viruses in human macrophages

**Figure 7:**
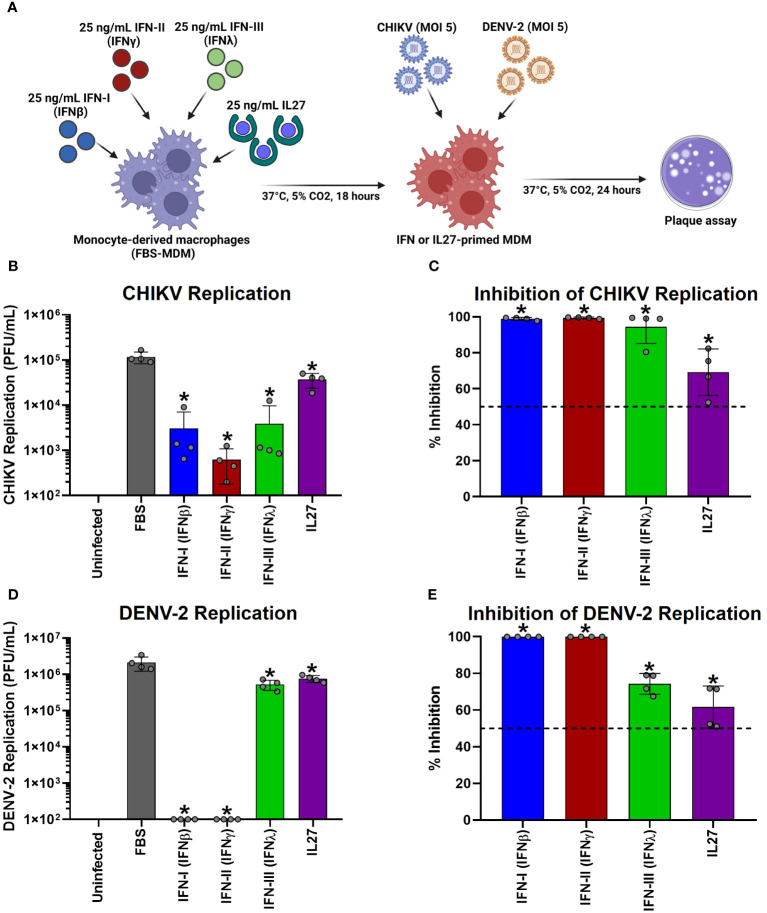
Interferons and Interleukin 27 interfered with the replication of chikungunya and dengue viruses in human macrophages. Human primary FBS-MDMs cultures (n= 4) were stimulated or not with 25 ng/mL recombinant-human IFN-I (IFNβ), IFN-II (IFNγ), IFN-III (IFNλ), or IL27 for 18 hours. Next, the FBS-MDM cultures were infected with CHIKV or DENV-2 at an MOI of 5. Culture supernatants were obtained at 24 hpi, and viral titration was performed by plaque assay on Vero or BHK-21 cells for CHIKV and DENV-2, respectively. The study flowchart shown in **(A)** was created using BioRender. CHIKV replication **(B)** and the percentage of viral inhibition in FBS-MDMs **(C)**. DENV-2 replication **(D)** and percentage of viral inhibition in FBS-MDMs **(E)**. Data are presented as the mean ± SD, data normality was evaluated using Shapiro-Wilk test, and One-way ANOVA with Fisher’s LSD post-test was performed. Significant differences between virus-infected FBS-MDMs and virus-infected IFN- or IL27-stimulated FBS-MDMs were defined as p<0.05 (*).

To test the effect of IL-27 stimulation of MDMs on viral replication, FBS-MDMs cultures (n= 4) were stimulated or not with 25 ng/mL recombinant human IFN-β, IFN-γ, IFN-λ, or IL-27 for 18 h. Subsequently, cells were infected with CHIKV or DENV-2 at MOI 5 and viral replication was evaluated at 24 h post-infection (hpi) by plaque assay on Vero or BHK-21 cells, respectively (**Figure 7A**). As we previously reported (45, 73), we observed that human MDMs are susceptible to CHIKV and DENV-2 infections and release a large number of infectious viral particles at 24 hpi (**Figures 7B, D**, respectively). Interestingly, treatment of MDMs with each type of IFNs or IL-27 for 18 h significantly decreased the release of infectious viral particles and promoted high inhibition of viral replication of CHIKV (**Figures 7B, C**) and DENV-2 (**Figures 7D, E**). It is worth noting that IFNs induced a higher control over CHIKV replication than IL-27 (**Figures 7B, C**). In contrast, type I and II IFNs showed strong antiviral activity against DENV-2 compared to IFN-III and IL-27, which showed similar control of DENV-2 replication in MDMs (**Figures 7D, E**). These results agree with our RNA-Seq and Microarray analysis, which showed that IL-27 significantly induced AVPs/ISGs expression (**Figure 2B**), but at lower levels than IFNs, especially IFN-I and IFN-III. Altogether, functional analysis demonstrated that like IFNs, IL-27-mediated response reduced chikungunya and dengue viruses replication in human MDMs, suggesting that IL-27 exhibits properties similar to those of all three types of human IFN, including the ability to stimulate a protective antiviral state that interfere with viral replication into the cells.

## 4 Discussion

The molecular structure of IL-27 and its receptor signaling complex (IL27R) has been described by Pflanz et al. (74, 75), and plays a significant role in triggering both pro- and anti-inflammatory responses in different immune cell populations. Although over 20 years have passed since their description, the molecular basis of IL-27 antiviral properties is only now beginning to be understood. The first report on the antiviral properties of IL-27 was by Fakruddin et al. (2007), who demonstrated that IL-27 was highly induced by PBMCs and human MDMs stimulated with non-infectious papillomavirus-like particles (76). The authors also reported that IL-27 treatment inhibits HIV-1 replication in CD4 + T cells, PBMCs, and MDMs in a dose-dependent manner. In a study by Bender et al. (2009), hepatoma cells and hepatocytes stimulated with IL-27 displayed sustained activation of STAT-1 and STAT-3, leading to an IFN-γ-like STAT-1-dependent response. This response induced interferon-regulated proteins, including STAT-1, STAT-2, IRF-1, IRF-9, and AVPs/ISGs such as GBP2 and MX1 (43), suggesting that IL-27 has IFNγ-like functions in liver cells. Furthermore, IL-27 has been shown to elicit a robust antiviral response against different acute and chronic viruses, including HIV-1 (19), HSV1 (20), HBV (21), HCV (22), IAV (23), ZIKV (24, 25), CHIKV (26), and SARS-CoV-2 (27) in their respective host cell types. Additionally, Kwock et al. (2020) demonstrated that IL-27 activates the JAK-STAT signaling pathway in HNEK and induces the expression of OAS1, OAS2, OASL, and MX1 in an IL-27Rα- and STAT-1-dependent manner. this induction occurs independently of IFNAR1 and STAT-2 (22). Furthermore, IL-27-treated HNEK cells inhibited ZIKV and Sendai virus replication *in vitro*. Using Ifnar1−/−, Il27ra−/−, and Ifnar1−/−/Il27ra−/− mice infected with ZIKV, Kwock et al. (2020) showed that subcutaneous administration of IL-27 reduced mortality and the onset of neurological symptoms of ZIKV infection in an IFN-independent manner (22). These results confirmed the ability of IL-27 to induce a protective antiviral response against ZIKV, both *in vitro* and *in vivo*.

**Figure 8:**
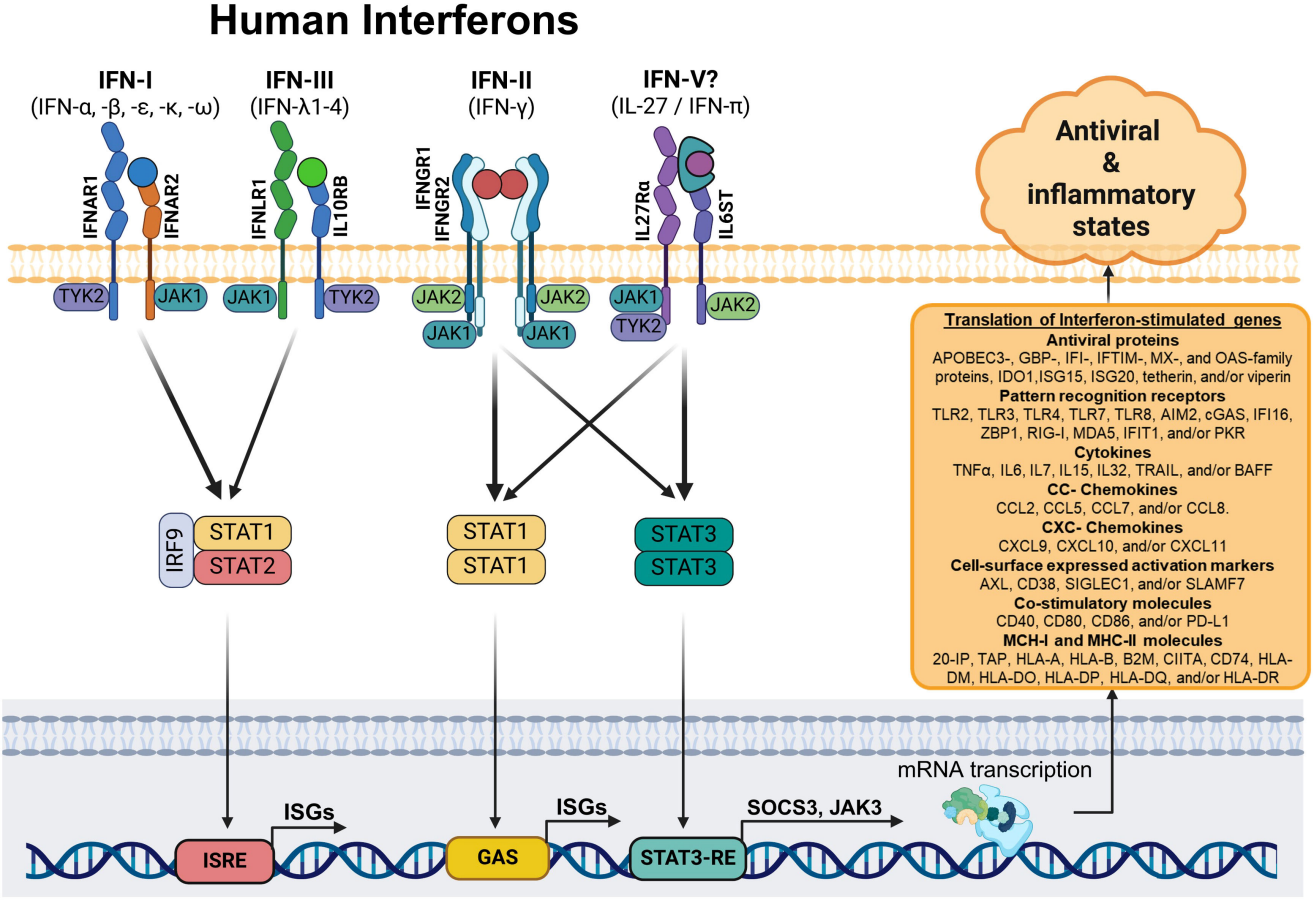
Schematic model: The interferon and interleukin 27-dependent JAK-STAT signaling pathway. Based on our results, we suggest that MDMs stimulated with type I, II, and III IFNs, or IL-27 (IFN-π)? activate different components of the JAK-STAT signaling pathway. Specifically, STAT1, the primary transcription factor involved in IFN-dependent signal transduction, forms a STAT1/STAT1 homodimer (for IFN-II and IL-27) or complex with STAT2 and IRF9 (for IFN-I and IFN-III). These complexes translocate into the nucleus and induce the coordinated expression of a robust ISG-dependent pro-inflammatory and antiviral programs that “interferes” with viral replication.

In agreement with these results, our researches showed that CHIKV-infected MDMs (26) and ZIKV-infected monocytes (25) exhibited high *IL27p28*/*EBI3* mRNA expression and IL-27 protein production. This upregulation was associated with the induction of AVPs/ISGs and control of viral replication in the absence of IFNs. In addition, treatment of human monocytes and MDMs with IL-27 inhibited the replication of ZIKV and CHIKV replication in a dose-dependent manner. Notably, PBMCs and monocytes from SARS-CoV-2-infected patients showed a robust *IL-27*-*STAT1*-dependent antiviral response as a function of a severe clinical course of COVID-19, in an IFN-independent manner (27). Furthermore, our RNA-Seq and Microarray analysis revealed that like interferons, IL-27 can activate the JAK-STAT signaling pathway in macrophages, as well as in other immune cell types (dendritic cells and T lymphocytes) and non-immune cells (keratinocytes and astrocytes). These data strongly support that IL-27 induces the expression of a broad range of ISGs, including AVPs and PRRs, although the expression of some of these genes may be cell-lineage-dependent. Moreover, our study revealed that like IFNs, IL-27 hinders the replication of CHIKV and DENV-2 in human primary MDMs. Hence, IL-27 shares similar properties with all three types of human IFN, including its role in a protective antiviral response. Based on the background presented above and the transcriptional profile showed in the present study, we propose that IL-27 could function as an interferon, which we hypothesized thus to be IFN-pi (IFN-π), constituting the fifth type of IFN (IFN-V) (**Figure 8**). However, to demonstrate this hypothesis, further studies are necessary to better understand the precise mechanism involved in the IL-27 functionality. These studies may include phylogenetic/evolutive analysis of IL-27 subunits, IFNs, IFNRs and IL27R, as well as additional protein/functional analysis.

Despite its crucial role in inducing the antiviral response, an uncontrolled or exacerbated production of IFNs and IL-27 could also contribute to the induction of immunopathology. Our RNA-Seq and Microarray data revealed that all three types of IFN and/or IL-27, particularly IFN-I and IFN-II, not only possess antiviral properties but also induce the expression of a wide array of STAT-1-dependent pro-inflammatory factors. These factors include CC- and CXC-motif chemokines such as CCL-2, *CCL5*, *CCL8*, *CXCL9*, *CXCL10*, and/or *CXCL11*. All these chemokines play pivotal roles in recruiting and activating various immune cell populations at the sites of infection. Additionally, IFNs and/or IL-27 promote the expression of different cytokines with inflammatory properties in both immune and non-immune cell populations. These include IL-7 which supports the development and homeostasis of T cells, B cells, and NK cells (77). IL-15, which aids in NK cell recruitment, differentiation, and cytotoxic/antiviral functions (42, 59); BAFF, crucial for B cells proliferation, differentiation, and antibodies production (78, 79). TRAIL, a member of TNF-superfamily with pro-apoptotic, anti-proliferative, and anti-tumoral properties (80). Together, our results suggest that pro-inflammatory functions of IFNs and IL-27 signaling promote a link with different immune cell populations, thus promoting more complex innate and adaptative immune responses.

It has been reported that IFN-II (IFN-γ) serves a link between innate and adaptative immune responses by inducing co-stimulatory molecules and MHC-II molecules, which promote antigen presentation to T cells by APCs (11, 13, 81). In our study, we found that, like IFN-II, IL-27 also significantly promotes the expression of *CIITA*, *CD74*, and MHC-II molecules, including *HLA-DOA1* and *HLA-DRA*. These results align with previous reports showing that the transcriptional expression of MHC-II molecules is regulated by STAT-1/STAT-1 homodimers, which translocate into the nucleus to promote CIITA and MHC-II genes expression in APCs (81). Like IFN-II, IFN-I (IFN-α and -β) induces the high expression of cell-surface-expressed immune activation markers such as *CD38* and *SLAMF7*; co-stimulatory molecules such as CD40, CD80, CD86, and *PD-L1*; 20S-IP components including *LMP2*; and MHC-encoded TAP components, including *TAP1*. Additionally, MHC-I molecules, including *HLA-A* and *B2M*, are upregulated by IFN-I. In contrast, IFN-III and IL-27 induce low mRNA and protein expression of all these factors, suggesting that IFN-I and IFN-II are more potent promoters of macrophage activation than IFN-III and IL-27.

The dysregulation of IFNs signaling have been implicated in the pathogenesis of a number of rheumatic diseases, including systemic lupus erythematosus, Sjögren syndrome, myositis, systemic sclerosis, and rheumatoid arthritis (82–84). Furthermore, various reports suggest a potential immunopathological role of IL-27 in chronic inflammatory diseases, including chronic CHIKV-dependent arthritis, and rheumatoid arthritis. High levels of IL-27 in serum and/or synovia from patients with these pathologies have been correlated with a persistent inflammatory state, immunopathology, and the clinical course of disease (36, 85–87). Moreover, high levels of inflammatory/arthrogenic factors, including cytokines (TNF-α, IL-6, IL-7, IL-15, and BAFF) and chemokines (CCL-2, CCL-5, and CXCL-10) have been reported in the serum and/or synovia of rheumatoid arthritis patients (88–90). These inflammatory factors are highly induced by all types of IFNs and/or IL-27 in different cell populations. This suggests that modulation or inhibition of the pro-inflammatory properties of IFNs and IL-27 signaling could be an interesting therapeutic target for controlling such immunopathology in patients. Furthermore, antiviral therapies based on the use of recombinant IFN-I and IFN-II are associated with counterproductive side effects in patients, mainly due to the high inflammatory properties of interferons (91, 92). Here, we report that unlike IFN-I and IFN-II, which promote a robust antiviral state along with a high pro-inflammatory response, IFN-III and IL-27 promote a robust antiviral state but induce a lower pro-inflammatory response than IFN-I and IFN-II. This suggests that IFN-III and IL-27 could be considered new therapeutic alternatives to help control viral infections without triggering an exacerbated inflammatory response in patients.

Our study demonstrated that IFNs, as well as IL-27, promote the induction of an antiviral state in human MDMs by activating JAK-STAT signaling and promoting a wide range of AVPs/ISGs. Importantly, IFNs and IL-27 also induced the expression of diverse ISGs that encode PRRs, which are critical components of the innate immune response. These receptors are essential for the recognition of PAMPs, which in the case of viruses, include non-structural proteins, membrane-integrated glycoproteins, modified lipids, and various types of nucleic acids. This recognition leads to the activation of pro-inflammatory and antiviral pathways and triggers an alarm state in uninfected or bystander cells (3, 41, 70, 93–96). This heightened sensitivity enables these cells to detect viral antigens and amplify their innate immune antiviral response. In general, the activation of TLRs, NLRs, and certain viral DNA and RNA sensors promote the activation of NF-kB complex, resulting in a robust NF-kB-dependent pro-inflammatory response (41, 93, 96). This occurs in parallel with the STAT-1-dependent pro-inflammatory response induced by IFNs or IL-27. Moreover, activation of some PRRs leads to the activation of IRF-1, IRF-3, and/or IRF-7, which in turn, induce the expression of IFN-I, IFN-III, and IL27-p28 (41, 93, 95–97). This creates a positive feedback loop between the IFN/IL-27 pathway and the PRRs system to amplify inflammatory and antiviral responses until the infection is controlled or resolved.

In line with this, we previously reported that both IFNs and IL-27 induce the expression of various families of PRRs. For instance, *TLR3*, a TRIF-dependent TLR that plays an important role in sensing viral dsRNA, leads to the activation of NF-kB, IRF-1, IRF-3, and/or IRF-7, which induces an inflammatory response and the production of IFN-β and IL27-p28 (41). *AIM2* is involved in the recognition of viral nucleic acids and intermediates of viral replication, as well as in activating the inflammasome complex (98). *cGAS* is involved in IFN-I induction by activating the cGAS-STING pathway (97). *ZBP1* is involved in the induction of IFN-I and PANoptosis death in response to z-DNA (99). *RIG-I* recognizes cytoplasmic 5’-triphosphate RNAs (ppp-RNA), short 5’-Cap0 ssRNAs, and dsRNAs (100–102), while *MDA5* recognizes long 5’-Cap0 dsRNAs (101, 103). Both RIG-I and MDA5 promote the activation of IRFs and induce a high production of IFN-I and IFN-III (101). Additionally, IFNs and IL-27 induce significant expression of AVPs/ISGs, including *IFIT1*, which recognizes viral ppp-RNAs and inhibits viral RNA translation (104); and *PKR*, which is activated in response to cytoplasmic dsRNA. PKR mediates a shutdown of translation within cells through phosphorylation of eIF2α, inducing the integrated-stress response and promoting the inhibition of translation of cap-dependent cellular and viral mRNAs (105, 106).

Thus, our transcriptomic analysis of IFN- and IL-27-dependent signaling pathways revealed that all types of IFNs and IL-27 induce an alarm state in cells by promoting the expression of TLRs, NLRs, RLRs, and/or other viral DNA and RNA sensors. All these factors are involved in the recognition of viral PAMPs, and they contribute to the amplification of NF-κB-dependent and IFN/IL-27-STAT-1-dependent pro-inflammatory and antiviral responses. These functions may be crucial in the control of viral spread into tissues, but they may also play a role in the development of inflammatory autoimmune diseases.

## 5 Limitations of study

This research was limited in several ways. While our research demonstrated transcriptional changes and previous studies have indicating the activation of JAK-STAT signaling in MDMs following stimulation with various types of IFN or IL-27 (14, 15, 17, 24, 43), we did not assess the precise mechanisms or protein levels of key factors such as STAT-1, p-STAT-1, or AVPs/ISGs. Nevertheless, previous research has established a correlation between p-STAT-1 and/or *STAT1* mRNA levels with the induction of AVPs/ISGs expression and entablement of antiviral state in macrophages (26, 41, 107). Second, despite our *in vitro* model utilizing primary human macrophages providing a clear characterization of the pro-inflammatory and antiviral properties of different types of IFN and IL-27, additional experiments are necessary to gain a more comprehensive understanding of the role of IL-27 signaling pathways in inducing the antiviral state in an *in vivo* model. Third, it should be noted that RNA-Seq of IFN-I was performed using IFN-α, although IFN-β was used for experimental validation. Furthermore, we recognize that concentration and duration of IL-27 stimulation in FBS-MDMs may not be optimal. It´s noteworthy that IL-27 signaling promote a high expression of SOCS3, its main negative regulation, which could contribute to the lower response observed with IL-27 at 18 h as compared to other IFNs.

## 6 Conclusions

In conclusion, our RNA-Seq analysis revealed the transcriptional profile of FBS-MDMs stimulated by IL-27 and identified similarities in their mRNA profile compared to IFN-stimulated HS-MDMs. Our findings indicate that both IFNs and IL-27 induce transcriptional change to various gene, including those involved in JAK-STAT signaling, and lead to a significant upregulation of ISGs associated with innate immune antiviral and pro-inflammatory responses. However, IFNs appear to be more potent than IL-27. These findings offer new insights into virus-host interactions and may contribute in the development of strategies utilizing both IFNs and IL-27 against viral agents. Furthermore, our study revealed that, like IFNs, IL-27 promotes a robust antiviral state that interfere with CHIKV and DENV-2 replication in human primary MDMs. This shown that IL-27 shares similar antiviral properties with all three types of human IFN. Building upon these results and in alignment with previous reports highlighting the STAT-1-dependent antiviral properties of IL-27 signaling (17, 24), we propose that IL-27 functions as an IFN. However, further studies should focus on elucidating the non-transcriptional cellular responses regulated by IL-27.

## Data availability statement

The data presented in the study are deposited in the GEO repository, accession number GSE262963.

## Ethics statement

The study was approved by the Ethics Committee of the “Sede de Investigación Universitaria-Universidad de Antioquia.” The studies were conducted in accordance with the local legislation and institutional requirements. The participants provided their written informed consent to participate in this study. Ethical approval was not required for the studies on animals in accordance with the local legislation and institutional requirements because only commercially available established cell lines were used.

## Author contributions

JV-L: Conceptualization, Data curation, Formal analysis, Investigation, Methodology, Supervision, Visualization, Writing – original draft, Writing – review & editing. LH-S: Conceptualization, Investigation, Methodology, Validation, Writing – review & editing. YT-M: Conceptualization, Data curation, Formal analysis, Investigation, Methodology, Writing – review & editing. PV: Conceptualization, Resources, Supervision, Writing – review & editing. IR-Z: Conceptualization, Formal analysis, Supervision, Writing – review & editing. SU-I: Conceptualization, Formal analysis, Project administration, Resources, Supervision, Writing – original draft, Writing – review & editing.

## Supplementary material

Supplementary Figure 1 Morphologic and phenotypic characterization of monocyte-derived macrophages (MDMs). Human monocytes were cultured in RPMI-1640 medium supplemented with 10% FBS for 7 days to obtain FBS-MDMs. These cultures then stained with SYBR-Green, and photomicrographs were captured using phase-contrast and epi-fluorescence microscopy. Representative images of cell morphology **(A)** and nuclear-cytoplasmic ratio **(B)** of FBS-MDMs are shown. The gating strategy used for flow cytometry analysis is depicted in **(C)**. Cell viability was assessed by staining FBS-MDMs cultures with fixable viability dye. The expression of CD68 **(D)**, CD40 **(E)**, and CD86 **(F)** on the cell surface of FBS-MDMs was analyzed by flow cytometry. The gray areas correspond to an appropriate isotype-control reaction. Data presented are representative of one of at least four independent experiments.

Supplementary Figure 2 Interferons and Interleukin 27 modulated the expression of housekeeping genes, macrophage markers, and/or IFN/IL27 receptor complexes in human macrophages. Human MDMs (n= 3) were stimulated or not with IFNs, or IL-27 as shown in A/B. Gene expression (mRNA) of housekeeping genes **(A)**, macrophage markers **(B)**, and IFN/IL-27 receptors **(C)** was expressed as TPM. Data are presented as mean ± SD. One-way ANOVA with Fisher’s LSD post-test was performed. Significant results between unstimulated HS-MDMs and IFN-stimulated HS-MDMs, or unstimulated FBS-MDMs and IL27-stimulated FBS-MDMs are defined as p<0.05 (*).

Supplementary Table 1 Gene-specific primers used for validation of RNA-seq data using RT-qPCR.

Supplementary Table 2 RT-qPCR data.

Supplementary Table 3 Statistical analysis results.
