# Supplementary figures and images for: Interleukin 27, like interferons, activates JAK-STAT signaling and promotes pro-inflammatory and antiviral states that interfere with dengue and chikungunya viruses replication in human macrophages
Source: Front Immunol. 2024 Apr 24;15:1385473. doi: 10.3389/fimmu.2024.1385473 (PMC11076713; doi:10.3389/fimmu.2024.1385473)

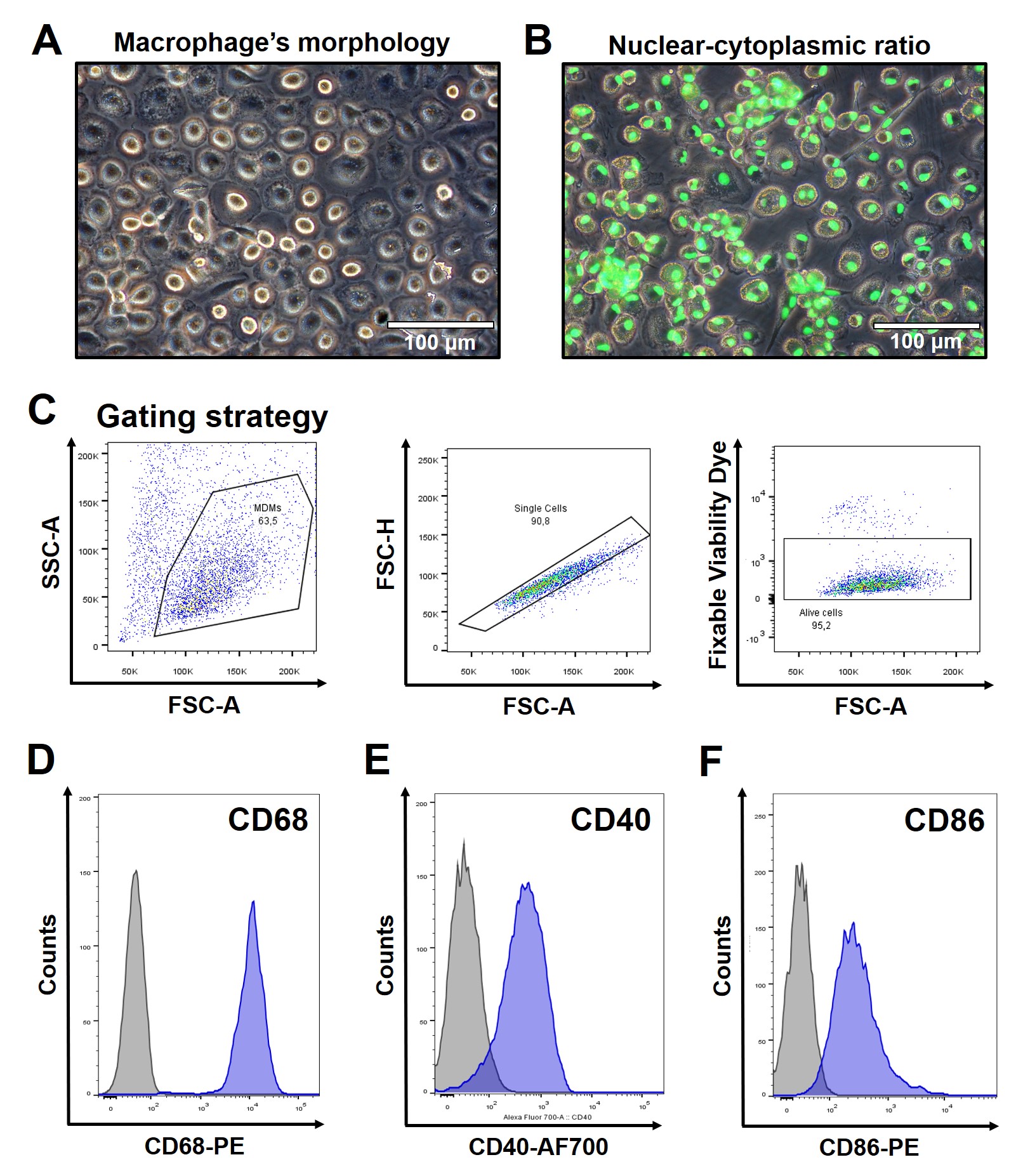

Supplement: Supplementary Figure 1 — Morphologic and phenotypic characterization of monocyte-derived macrophages (MDMs). Human monocytes were cultured in RPMI-1640 medium supplemented with 10% FBS for 7 days to obtain FBS-MDMs. These cultures then stained with SYBR-Green, and photomicrographs were captured using phase-contrast and epi-fluorescence microscopy. Representative images of cell morphology (A) and nuclear-cytoplasmic ratio (B) of FBS-MDMs are shown. The gating strategy used for flow cytometry analysis is depicted in (C). Cell viability was assessed by staining FBS-MDMs cultures with fixable viability dye. The expression of CD68 (D), CD40 (E), and CD86 (F) on the cell surface of FBS-MDMs was analyzed by flow cytometry. The gray areas correspond to an appropriate isotype-control reaction. Data presented are representative of one of at least four independent experiments. [file Image_1.jpeg]

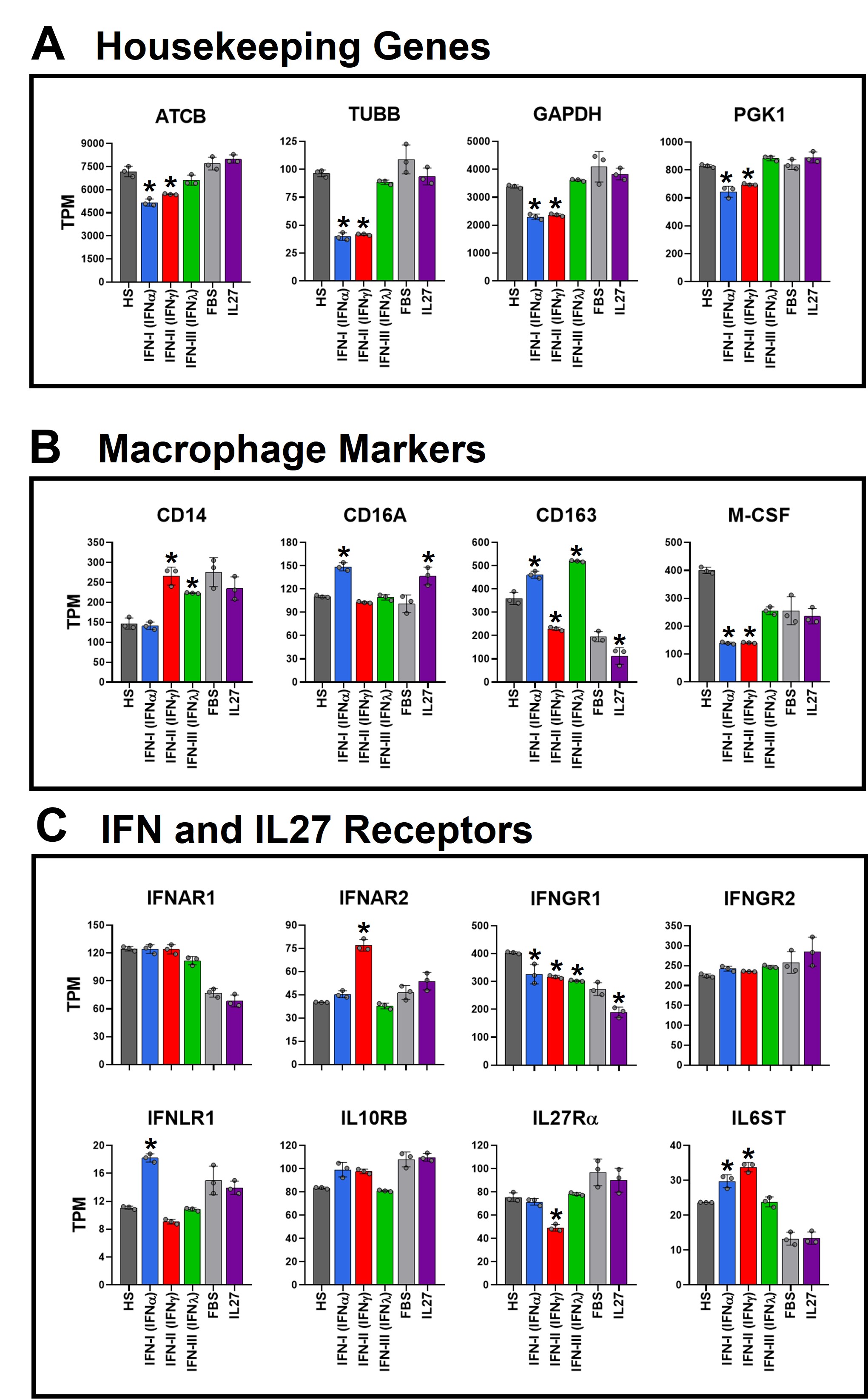

Supplement: Supplementary Figure 2 — Interferons and Interleukin 27 modulated the expression of housekeeping genes, macrophage markers, and/or IFN/IL27 receptor complexes in human macrophages. Human MDMs (n= 3) were stimulated or not with IFNs, or IL-27 as shown in A/B. Gene expression (mRNA) of housekeeping genes (A), macrophage markers (B), and IFN/IL-27 receptors (C) was expressed as TPM. Data are presented as mean ± SD. One-way ANOVA with Fisher’s LSD post-test was performed. Significant results between unstimulated HS-MDMs and IFN-stimulated HS-MDMs, or unstimulated FBS-MDMs and IL27-stimulated FBS-MDMs are defined as p<0.05 (*). [file Image_2.jpeg]
