# Supplementary material for: Interleukin 27, like interferons, activates JAK-STAT signaling and promotes pro-inflammatory and antiviral states that interfere with dengue and chikungunya viruses replication in human macrophages
Source: Front Immunol. 2024 Apr 24;15:1385473. doi: 10.3389/fimmu.2024.1385473 (PMC11076713; doi:10.3389/fimmu.2024.1385473)
Supplement: Supplementary Table 3 — Statistical analysis results. [file DataSheet_1.pdf]

TNF TPM DATA

Normality and Lognormality Tests		A	B	C	D	E	F
		**HS**	**IFN-I (IFNα)**	**IFN-II (IFNγ)**	**IFN-III (IFNλ)**	**FBS**	**IL27**
1	**Test for normal distribution**						
2	**Shapiro-Wilk test**						
3	W	0.8074	0.9925	0.9435	0.9373	0.8212	0.7580
4	P value	0.1323	0.8339	0.5416	0.5165	0.1662	0.0177
5	Passed normality test (alpha=0.05)?	Yes	Yes	Yes	Yes	Yes	No
6	P value summary	ns	ns	ns	ns	ns	*

**Ordinary one-way ANOVA**<br>ANOVA results						
1	**ANOVA summary**					
2	F	16.46				
3	P value	<0.0001				
4	P value summary	****				
5	Significant diff. among means (P < 0.05)?	Yes				
6	R square	0.8727				
7						
8	**Brown-Forsythe test**					
9	F (DFn, DFd)	1.027 (5, 12)				
10	P value	0.4445				
11	P value summary	ns				
12	Are SDs significantly different (P < 0.05)?	No				
13						
14	**ANOVA table**	**SS**	**DF**	**MS**	**F (DFn, DFd)**	**P value**
15	Treatment (between columns)	14.08	5	2.815	F (5, 12) = 16.46	P<0.0001
16	Residual (within columns)	2.053	12	0.1711		
18	Total	16.13	17			

**Ordinary one-way ANOVA**<br>Multiple comparisons						
1	**Uncorrected Fisher's LSD**	**Mean Diff.**	**95.00% CI of diff.**	**Significant?**	**Summary**	**Individual P Value**
2	**HS** vs. **IFN-I (IFNα)**	-0.2740	-1.010 to -0.4617	Yes	*	0.0329
3	**HS** vs. **IFN-II (IFNγ)**	-2.615	-3.350 to -1.879	Yes	****	<0.0001
4	**HS** vs. **IFN-III (IFNλ)**	-0.2358	-0.9716 to -0.5000	Yes	*	0.0498
5	**FBS** vs. **IL27**	-0.1606	-0.8964 to 0.5751	No	ns	0.6428

IL6 TPM DATA

Normality and Lognormality Tests		A	B	C	D	E	F
		HS	IFN-I (IFNα)	IFN-II (IFNγ)	IFN-III (IFNλ)	FBS	IL27
1	**Test for normal distribution**						
2	**Shapiro-Wilk test**						
3	W	Invalid input data	0.8521	0.7564	Invalid input data	Invalid input data	0.9620
4	P value		0.2462	0.0141			0.6252
5	Passed normality test (alpha=0.05)?		Yes	No			Yes
6	P value summary		ns	*			ns

Ordinary one-way ANOVA
ANOVA results

1	**ANOVA summary**					
2	F	14.26				
3	P value	0.0001				
4	P value summary	***				
5	Significant diff. among means (P < 0.05)?	Yes				
6	R square	0.8560				
7						
8	**Brown-Forsythe test**					
9	F (DFn, DFd)	1.340 (5, 12)				
10	P value	0.3127				
11	P value summary	ns				
12	Are SDs significantly different (P < 0.05)?	No				
13						
14	**ANOVA table**	**SS**	**DF**	**MS**	**F (DFn, DFd)**	**P value**
15	Treatment (between columns)	0.8371	5	0.1674	F (5, 12) = 14.26	P=0.0001
16	Residual (within columns)	0.1409	12	0.01174		
18	Total	0.9780	17			

Ordinary one-way ANOVA
Multiple comparisons

1	**Uncorrected Fisher's LSD**	**Mean Diff.**	**95.00% CI of diff.**	**Significant?**	**Summary**	**Individual P Value**
2	**HS** vs. **IFN-I (IFNα)**	-0.6079	-0.8006 to -0.4152	Yes	****	<0.0001
3	**HS** vs. **IFN-II (IFNγ)**	-0.1935	-0.3862 to -0.0007174	Yes	*	0.0493
4	**HS** vs. **IFN-III (IFNλ)**	0.000	-0.1927 to 0.1927	No	ns	>0.9999
5	**FBS** vs. **IL27**	-0.1850	-0.3778 to -0.007716	No	*	0.0484

IL7 TPM DATA

	Normality and Lognormality Tests	A	B	C	D	E	F
		HS	IFN-I (IFNα)	IFN-II (IFNy)	IFN-III (IFNλ)	FBS	IL27
1	**Test for normal distribution**						
2	**Shapiro-Wilk test**						
3	W	0.8393	0.9201	0.8358	0.8847	0.9763	0.8244
4	P value	0.2122	0.4526	0.2031	0.3383	0.7050	0.1743
5	Passed normality test (alpha=0.05)?	Yes	Yes	Yes	Yes	Yes	Yes
6	P value summary	ns	ns	ns	ns	ns	ns

	**Ordinary one-way ANOVA** ANOVA results					
1	**ANOVA summary**					
2	F	23.89				
3	P value	<0.0001				
4	P value summary	****				
5	Significant diff. among means (P < 0.05)?	Yes				
6	R square	0.9087				
7						
8	**Brown-Forsythe test**					
9	F (DFn, DFd)	0.1357 (5, 12)				
10	P value	0.9808				
11	P value summary	ns				
12	Are SDs significantly different (P < 0.05)?	No				
13						
14	**ANOVA table**	**SS**	**DF**	**MS**	**F (DFn, DFd)**	**P value**
15	Treatment (between columns)	10.33	5	2.066	F (5, 12) = 23.89	P<0.0001
16	Residual (within columns)	1.038	12	0.08650		
18	Total	11.37	17			

	**Ordinary one-way ANOVA** Multiple comparisons					
1	**Uncorrected Fisher's LSD**	**Mean Diff.**	**95.00% CI of diff.**	**Significant?**	**Summary**	**Individual P Value**
2	**HS** vs. **IFN-I (IFNα)**	-1.778	-2.301 to -1.255	Yes	****	<0.0001
3	**HS** vs. **IFN-II (IFNy)**	-1.397	-1.920 to -0.8740	Yes	****	<0.0001
4	**HS** vs. **IFN-III (IFNλ)**	-1.018	-1.541 to -0.4943	Yes	**	0.0012
5	**FBS** vs. **IL27**	-1.725	-2.249 to -1.202	Yes	****	<0.0001

IL15 TPM DATA

Normality and Lognormality Tests		A	B	C	D	E	F
		**HS**	**IFN-I (IFNα)**	**IFN-II (IFNγ)**	**IFN-III (IFNλ)**	**FBS**	**IL27**
1	**Test for normal distribution**						
2	**Shapiro-Wilk test**						
3	W	0.9567	0.9658	0.9961	0.8926	0.8771	0.9683
4	P value	0.5997	0.6445	0.8812	0.3623	0.3160	0.6579
5	Passed normality test (alpha=0.05)?	Yes	Yes	Yes	Yes	Yes	Yes
6	P value summary	ns	ns	ns	ns	ns	ns

Ordinary one-way ANOVA ANOVA results						
1	**ANOVA summary**					
2	F	106.3				
3	P value	<0.0001				
4	P value summary	****				
5	Significant diff. among means (P < 0.05)?	Yes				
6	R square	0.9779				
7						
8	**Brown-Forsythe test**					
9	F (DFn, DFd)	1.727 (5, 12)				
10	P value	0.2030				
11	P value summary	ns				
12	Are SDs significantly different (P < 0.05)?	No				
13						
14	**ANOVA table**	**SS**	**DF**	**MS**	**F (DFn, DFd)**	**P value**
15	Treatment (between columns)	1082	5	216.4	F (5, 12) = 106.3	P<0.0001
16	Residual (within columns)	24.42	12	2.035		
18	Total	1106	17			

Ordinary one-way ANOVA Multiple comparisons						
1	**Uncorrected Fisher's LSD**	**Mean Diff.**	**95.00% CI of diff.**	**Significant?**	**Summary**	**Individual P Value**
2	**HS** vs. **IFN-I (IFNα)**	-15.16	-17.70 to -12.63	Yes	****	<0.0001
3	**HS** vs. **IFN-II (IFNγ)**	-18.87	-21.40 to -16.33	Yes	****	<0.0001
4	**HS** vs. **IFN-III (IFNλ)**	-4.179	-6.717 to -1.641	Yes	**	0.0037
5	**FBS** vs. **IL27**	-15.10	-17.64 to -12.56	Yes	****	<0.0001

IL32 TPM DATA

Normality and Lognormality Tests		A	B	C	D	E	F
		**HS**	**IFN-I (IFNα)**	**IFN-II (IFNγ)**	**IFN-III (IFNλ)**	**FBS**	**IL27**
1	**Test for normal distribution**						
2	**Shapiro-Wilk test**						
3	W	0.9151	0.9920	0.7521	0.7705	1.000	0.9795
4	P value	0.4355	0.8286	0.0047	0.0458	0.9995	0.7257
5	Passed normality test (alpha=0.05)?	Yes	Yes	No	No	Yes	Yes
6	P value summary	ns	ns	**	*	ns	ns

**Ordinary one-way ANOVA** ANOVA results						
1	**ANOVA summary**					
2	F	65.71				
3	P value	<0.0001				
4	P value summary	****				
5	Significant diff. among means (P < 0.05)?	Yes				
6	R square	0.9648				
7						
8	**Brown-Forsythe test**					
9	F (DFn, DFd)	1.752 (5, 12)				
10	P value	0.1975				
11	P value summary	ns				
12	Are SDs significantly different (P < 0.05)?	No				
13						
14	**ANOVA table**	**SS**	**DF**	**MS**	**F (DFn, DFd)**	**P value**
15	Treatment (between columns)	248.2	5	49.64	F (5, 12) = 65.71	P<0.0001
16	Residual (within columns)	9.066	12	0.7555		
18	Total	257.3	17			

**Ordinary one-way ANOVA** Multiple comparisons						
1	**Uncorrected Fisher's LSD**	**Mean Diff.**	**95.00% CI of diff.**	**Significant?**	**Summary**	**Individual P Value**
2	**HS** vs. **IFN-I (IFNα)**	0.05408	-1.492 to 1.600	No	ns	0.9405
3	**HS** vs. **IFN-II (IFNγ)**	-10.05	-11.60 to -8.507	Yes	****	<0.0001
4	**HS** vs. **IFN-III (IFNλ)**	0.1888	-1.358 to 1.735	No	ns	0.7948
5	**FBS** vs. **IL27**	-3.369	-4.915 to -1.823	Yes	***	0.0005

TRAIL TPM DATA

Normality and Lognormality Tests		A	B	C	D	E	F
		HS	IFN-I (IFNα)	IFN-II (IFNγ)	IFN-III (IFNλ)	FBS	IL27
1	**Test for normal distribution**						
2	**Shapiro-Wilk test**						
3	W	0.9836	0.9638	0.9998	0.9982	0.8061	0.9760
4	P value	0.7546	0.6345	0.9707	0.9179	0.1291	0.7026
5	Passed normality test (alpha=0.05)?	Yes	Yes	Yes	Yes	Yes	Yes
6	P value summary	ns	ns	ns	ns	ns	ns

Ordinary one-way ANOVA<br>ANOVA results						
1	**ANOVA summary**					
2	F	1164				
3	P value	<0.0001				
4	P value summary	****				
5	Significant diff. among means (P < 0.05)?	Yes				
6	R square	0.9979				
7						
8	**Brown-Forsythe test**					
9	F (DFn, DFd)	1.884 (5, 12)				
10	P value	0.1710				
11	P value summary	ns				
12	Are SDs significantly different (P < 0.05)?	No				
13						
14	**ANOVA table**	**SS**	**DF**	**MS**	**F (DFn, DFd)**	**P value**
15	Treatment (between columns)	283485	5	56697	F (5, 12) = 1164	P<0.0001
16	Residual (within columns)	584.3	12	48.69		
18	Total	284069	17			

Ordinary one-way ANOVA<br>Multiple comparisons						
1	**Uncorrected Fisher's LSD**	**Mean Diff.**	**95.00% CI of diff.**	**Significant?**	**Summary**	**Individual P Value**
2	**HS** vs. **IFN-I (IFNα)**	-369.4	-381.8 to -357.0	Yes	****	<0.0001
3	**HS** vs. **IFN-II (IFNγ)**	-100.4	-112.9 to -88.02	Yes	****	<0.0001
4	**HS** vs. **IFN-III (IFNλ)**	-54.29	-66.71 to -41.88	Yes	****	<0.0001
5	**FBS** vs. **IL27**	-37.37	-49.78 to -24.95	Yes	****	<0.0001

BAFF TPM DATA

Normality and Lognormality Tests		A	B	C	D	E	F
		HS	IFN-I (IFNα)	IFN-II (IFNγ)	IFN-III (IFNλ)	FBS	IL27
1	**Test for normal distribution**						
2	**Shapiro-Wilk test**						
3	W	0.9092	0.9698	0.8786	0.9804	0.9999	0.8159
4	P value	0.4155	0.6665	0.3204	0.7318	0.9775	0.1531
5	Passed normality test (alpha=0.05)?	Yes	Yes	Yes	Yes	Yes	Yes
6	P value summary	ns	ns	ns	ns	ns	ns

Ordinary one-way ANOVA<br>ANOVA results						
1	**ANOVA summary**					
2	F	182.1				
3	P value	<0.0001				
4	P value summary	****				
5	Significant diff. among means (P < 0.05)?	Yes				
6	R square	0.9870				
7						
8	**Brown-Forsythe test**					
9	F (DFn, DFd)	1.741 (5, 12)				
10	P value	0.1999				
11	P value summary	ns				
12	Are SDs significantly different (P < 0.05)?	No				
13						
14	**ANOVA table**	**SS**	**DF**	**MS**	**F (DFn, DFd)**	**P value**
15	Treatment (between columns)	247895	5	49579	F (5, 12) = 182.1	P<0.0001
16	Residual (within columns)	3267	12	272.3		
18	Total	251162	17			

Ordinary one-way ANOVA<br>Multiple comparisons						
1	**Uncorrected Fisher's LSD**	**Mean Diff.**	**95.00% CI of diff.**	**Significant?**	**Summary**	**Individual P Value**
2	**HS** vs. **IFN-I (IFNα)**	-313.0	-342.3 to -283.6	Yes	****	<0.0001
3	**HS** vs. **IFN-II (IFNγ)**	-215.7	-245.0 to -186.3	Yes	****	<0.0001
4	**HS** vs. **IFN-III (IFNλ)**	-106.4	-135.8 to -77.05	Yes	****	<0.0001
5	**FBS** vs. **IL27**	-32.19	-61.55 to -2.839	Yes	*	0.0342

CCL2 TPM DATA

Normality and Lognormality Tests		A	B	C	D	E	F
		HS	IFN-I (IFNα)	IFN-II (IFNγ)	IFN-III (IFNλ)	FBS	IL27
1	**Test for normal distribution**						
2	**Shapiro-Wilk test**						
3	W	0.7613	0.8263	0.9693	0.9545	0.9989	0.8308
4	P value	0.0251	0.1790	0.6638	0.5896	0.9372	0.1903
5	Passed normality test (alpha=0.05)?	No	Yes	Yes	Yes	Yes	Yes
6	P value summary	*	ns	ns	ns	ns	ns

**Ordinary one-way ANOVA** ANOVA results						
1	**ANOVA summary**					
2	F	20.62				
3	P value	<0.0001				
4	P value summary	****				
5	Significant diff. among means (P < 0.05)?	Yes				
6	R square	0.8957				
7						
8	**Brown-Forsythe test**					
9	F (DFn, DFd)	1.058 (5, 12)				
10	P value	0.4296				
11	P value summary	ns				
12	Are SDs significantly different (P < 0.05)?	No				
13						
14	**ANOVA table**	**SS**	**DF**	**MS**	**F (DFn, DFd)**	**P value**
15	Treatment (between columns)	188391	5	37678	F (5, 12) = 20.62	P<0.0001
16	Residual (within columns)	21930	12	1828		
18	Total	210321	17			

**Ordinary one-way ANOVA** Multiple comparisons						
1	**Uncorrected Fisher's LSD**	**Mean Diff.**	**95.00% CI of diff.**	**Significant?**	**Summary**	**Individual P Value**
2	**HS** vs. **IFN-I (IFNα)**	-191.8	-267.9 to -115.8	Yes	***	0.0001
3	**HS** vs. **IFN-II (IFNγ)**	-126.5	-202.5 to -50.44	Yes	**	0.0035
4	**HS** vs. **IFN-III (IFNλ)**	-148.1	-224.1 to -72.01	Yes	**	0.0011
5	**FBS** vs. **IL27**	154.0	77.96 to 230.1	Yes	***	0.0008

CCL5 TPM DATA

	Normality and Lognormality Tests	A<br>HS	B<br>IFN-I (IFNα)	C<br>IFN-II (IFNγ)	D<br>IFN-III (IFNλ)	E<br>FBS	F<br>IL27
1	**Test for normal distribution**						
2	**Shapiro-Wilk test**						
3	W	0.9544	0.8975	0.8573	0.9308	0.8066	0.7823
4	P value	0.5888	0.3776	0.2601	0.4916	0.1303	0.0730
5	Passed normality test (alpha=0.05)?	Yes	Yes	Yes	Yes	Yes	Yes
6	P value summary	ns	ns	ns	ns	ns	ns

	**Ordinary one-way ANOVA**<br>ANOVA results					
1	**ANOVA summary**					
2	F	98.50				
3	P value	<0.0001				
4	P value summary	****				
5	Significant diff. among means (P < 0.05)?	Yes				
6	R square	0.9762				
7						
8	**Brown-Forsythe test**					
9	F (DFn, DFd)	0.6289 (5, 12)				
10	P value	0.6815				
11	P value summary	ns				
12	Are SDs significantly different (P < 0.05)?	No				
13						
14	**ANOVA table**	**SS**	**DF**	**MS**	**F (DFn, DFd)**	**P value**
15	Treatment (between columns)	124763	5	24953	F (5, 12) = 98.50	P<0.0001
16	Residual (within columns)	3040	12	253.3		
18	Total	127803	17			

	**Ordinary one-way ANOVA**<br>Multiple comparisons					
1	**Uncorrected Fisher's LSD**	**Mean Diff.**	**95.00% CI of diff.**	**Significant?**	**Summary**	**Individual P Value**
2	**HS** vs. **IFN-I (IFNα)**	-258.4	-286.7 to -230.1	Yes	****	<0.0001
3	**HS** vs. **IFN-II (IFNγ)**	-48.32	-76.63 to -20.00	Yes	**	0.0029
4	**HS** vs. **IFN-III (IFNλ)**	-35.03	-63.34 to -6.711	Yes	*	0.0195
5	**FBS** vs. **IL27**	-25.28	-53.59 to 3.037	No	ns	0.0756

CCL7 TPM DATA

Normality and Lognormality Tests		A<br>HS	B<br>IFN-I (IFNα)	C<br>IFN-II (IFNγ)	D<br>IFN-III (IFNλ)	E<br>FBS	F<br>IL27
1	**Test for normal distribution**						
2	**Shapiro-Wilk test**						
3	W	0.9814	0.9636	0.9507	0.9961	0.9127	0.9441
4	P value	0.7386	0.6334	0.5723	0.8800	0.4272	0.5443
5	Passed normality test (alpha=0.05)?	Yes	Yes	Yes	Yes	Yes	Yes
6	P value summary	ns	ns	ns	ns	ns	ns

Ordinary one-way ANOVA<br>ANOVA results						
1	**ANOVA summary**					
2	F	73.91				
3	P value	<0.0001				
4	P value summary	****				
5	Significant diff. among means (P < 0.05)?	Yes				
6	R square	0.9685				
7						
8	**Brown-Forsythe test**					
9	F (DFn, DFd)	2.265 (5, 12)				
10	P value	0.1142				
11	P value summary	ns				
12	Are SDs significantly different (P < 0.05)?	No				
13						
14	**ANOVA table**	**SS**	**DF**	**MS**	**F (DFn, DFd)**	**P value**
15	Treatment (between columns)	241926	5	48385	F (5, 12) = 73.91	P<0.0001
16	Residual (within columns)	7856	12	654.7		
18	Total	249782	17			

Ordinary one-way ANOVA<br>Multiple comparisons						
1	**Uncorrected Fisher's LSD**	**Mean Diff.**	**95.00% CI of diff.**	**Significant?**	**Summary**	**Individual P Value**
2	**HS** vs. **IFN-I (IFNα)**	-318.3	-363.8 to -272.8	Yes	****	<0.0001
3	**HS** vs. **IFN-II (IFNγ)**	-51.74	-97.26 to -6.218	Yes	*	0.0291
4	**HS** vs. **IFN-III (IFNλ)**	-116.8	-162.3 to -71.26	Yes	***	0.0001
5	**FBS** vs. **IL27**	-17.44	-62.96 to 28.08	No	ns	0.4202

CCL8 TPM DATA

Normality and Lognormality Tests		A	B	C	D	E	F
		HS	IFN-I (IFNα)	IFN-II (IFNγ)	IFN-III (IFNλ)	FBS	IL27
1	**Test for normal distribution**						
2	**Shapiro-Wilk test**						
3	W	0.8821	0.9706	0.7805	0.9885	0.9895	0.8388
4	P value	0.3307	0.6707	0.0687	0.7944	0.8039	0.2109
5	Passed normality test (alpha=0.05)?	Yes	Yes	Yes	Yes	Yes	Yes
6	P value summary	ns	ns	ns	ns	ns	ns

Ordinary one-way ANOVA<br>ANOVA results						
1	**ANOVA summary**					
2	F	437.7				
3	P value	<0.0001				
4	P value summary	****				
5	Significant diff. among means (P < 0.05)?	Yes				
6	R square	0.9945				
7						
8	**Brown-Forsythe test**					
9	F (DFn, DFd)	1.712 (5, 12)				
10	P value	0.2064				
11	P value summary	ns				
12	Are SDs significantly different (P < 0.05)?	No				
13						
14	**ANOVA table**	**SS**	**DF**	**MS**	**F (DFn, DFd)**	**P value**
15	Treatment (between columns)	569370	5	113874	F (5, 12) = 437.7	P<0.0001
16	Residual (within columns)	3122	12	260.1		
18	Total	572492	17			

Ordinary one-way ANOVA<br>Multiple comparisons						
1	**Uncorrected Fisher's LSD**	**Mean Diff.**	**95.00% CI of diff.**	**Significant?**	**Summary**	**Individual P Value**
2	**HS** vs. **IFN-I (IFNα)**	-493.5	-522.2 to -464.8	Yes	****	<0.0001
3	**HS** vs. **IFN-II (IFNγ)**	-120.8	-149.5 to -92.10	Yes	****	<0.0001
4	**HS** vs. **IFN-III (IFNλ)**	-29.99	-58.68 to -1.292	Yes	*	0.0419
5	**FBS** vs. **IL27**	-1.384	-30.08 to -7.31	Yes	*	0.0481

CXCL9 TPM DATA							
Normality and Lognormality Tests		A	B	C	D	E	F
		HS	IFN-I (IFNα)	IFN-II (IFNγ)	IFN-III (IFNλ)	FBS	IL27
1	**Test for normal distribution**						
2	**Shapiro-Wilk test**						
3	W	0.7505	0.9965	0.8465	0.9580	Invalid input data	0.9469
4	P value	0.0010	0.8875	0.2313	0.6057		0.5558
5	Passed normality test (alpha=0.05)?	No	Yes	Yes	Yes		Yes
6	P value summary	**	ns	ns	ns		ns

Ordinary one-way ANOVA<br>ANOVA results						
1	**ANOVA summary**					
2	F	294.3				
3	P value	<0.0001				
4	P value summary	****				
5	Significant diff. among means (P < 0.05)?	Yes				
6	R square	0.9919				
7						
8	**Brown-Forsythe test**					
9	F (DFn, DFd)	1.507 (5, 12)				
10	P value	0.2591				
11	P value summary	ns				
12	Are SDs significantly different (P < 0.05)?	No				
13						
14	**ANOVA table**	**SS**	**DF**	**MS**	**F (DFn, DFd)**	**P value**
15	Treatment (between columns)	16427398	5	3285480	F (5, 12) = 294.3	P<0.0001
16	Residual (within columns)	133962	12	11163		
18	Total	16561360	17			

Ordinary one-way ANOVA<br>Multiple comparisons						
1	**Uncorrected Fisher's LSD**	**Mean Diff.**	**95.00% CI of diff.**	**Significant?**	**Summary**	**Individual P Value**
2	**HS** vs. **IFN-I (IFNα)**	-13.05	-201.0 to 174.9	No	ns	0.8823
3	**HS** vs. **IFN-II (IFNγ)**	-2567	-2755 to -2379	Yes	****	<0.0001
4	**HS** vs. **IFN-III (IFNλ)**	-0.4466	-188.4 to 187.5	No	ns	0.9960
5	**FBS** vs. **IL27**	-7.177	-195.1 to 180.8	No	ns	0.9351

CXCL10 TPM DATA

Normality and Lognormality Tests		A	B	C	D	E	F
		HS	IFN-I (IFNα)	IFN-II (IFNγ)	IFN-III (IFNλ)	FBS	IL27
1	**Test for normal distribution**						
2	**Shapiro-Wilk test**						
3	W	0.9998	0.9867	0.8268	0.8008	0.9943	0.9076
4	P value	0.9706	0.7795	0.1801	0.1165	0.8554	0.4102
5	Passed normality test (alpha=0.05)?	Yes	Yes	Yes	Yes	Yes	Yes
6	P value summary	ns	ns	ns	ns	ns	ns

**Ordinary one-way ANOVA**<br>ANOVA results						
1	**ANOVA summary**					
2	F	445.1				
3	P value	<0.0001				
4	P value summary	****				
5	Significant diff. among means (P < 0.05)?	Yes				
6	R square	0.9946				
7						
8	**Brown-Forsythe test**					
9	F (DFn, DFd)	1.396 (5, 12)				
10	P value	0.2933				
11	P value summary	ns				
12	Are SDs significantly different (P < 0.05)?	No				
13						
14	**ANOVA table**	**SS**	**DF**	**MS**	**F (DFn, DFd)**	**P value**
15	Treatment (between columns)	867238	5	173448	F (5, 12) = 445.1	P<0.0001
16	Residual (within columns)	4676	12	389.7		
18	Total	871913	17			

**Ordinary one-way ANOVA**<br>Multiple comparisons						
1	**Uncorrected Fisher's LSD**	**Mean Diff.**	**95.00% CI of diff.**	**Significant?**	**Summary**	**Individual P Value**
2	**HS** vs. **IFN-I (IFNα)**	-311.6	-346.7 to -276.5	Yes	****	<0.0001
3	**HS** vs. **IFN-II (IFNγ)**	-567.0	-602.1 to -531.9	Yes	****	<0.0001
4	**HS** vs. **IFN-III (IFNλ)**	-1.726	-36.84 to 33.39	No	ns	0.9165
5	**FBS** vs. **IL27**	-1.097	-36.21 to 34.02	No	ns	0.9469

CXCL11 TPM DATA

Normality and Lognormality Tests		A	B	C	D	E	F
		HS	IFN-I (IFNα)	IFN-II (IFNγ)	IFN-III (IFNλ)	FBS	IL27
1	**Test for normal distribution**						
2	**Shapiro-Wilk test**						
3	W	0.7500	0.9925	0.9568	0.7530	Invalid input data	0.7500
4	P value		0.8341	0.6002	0.0067		
5	Passed normality test (alpha=0.05)?		Yes	Yes	No		
6	P value summary		ns	ns	**		

**Ordinary one-way ANOVA** ANOVA results						
1	**ANOVA summary**					
2	F	209.5				
3	P value	<0.0001				
4	P value summary	****				
5	Significant diff. among means (P < 0.05)?	Yes				
6	R square	0.9887				
7						
8	**Brown-Forsythe test**					
9	F (DFn, DFd)	2.814 (5, 12)				
10	P value	0.0659				
11	P value summary	ns				
12	Are SDs significantly different (P < 0.05)?	No				
13						
14	**ANOVA table**	**SS**	**DF**	**MS**	**F (DFn, DFd)**	**P value**
15	Treatment (between columns)	32730	5	6546	F (5, 12) = 209.5	P<0.0001
16	Residual (within columns)	375.0	12	31.25		
18	Total	33105	17			

**Ordinary one-way ANOVA** Multiple comparisons						
1	**Uncorrected Fisher's LSD**	**Mean Diff.**	**95.00% CI of diff.**	**Significant?**	**Summary**	**Individual P Value**
2	**HS** vs. **IFN-I (IFNα)**	-113.6	-123.6 to -103.7	Yes	****	<0.0001
3	**HS** vs. **IFN-II (IFNγ)**	-49.40	-59.35 to -39.46	Yes	****	<0.0001
4	**HS** vs. **IFN-III (IFNλ)**	-0.1261	-10.07 to 9.819	No	ns	0.9784
5	**FBS** vs. **IL27**	-0.2402	-10.19 to 9.705	No	ns	0.9589

CXCL13 TPM DATA							
Normality and Lognormality Tests		A	B	C	D	E	F
		HS	IFN-I (IFNα)	IFN-II (IFNγ)	IFN-III (IFNλ)	FBS	IL27
1	**Test for normal distribution**						
2	**Shapiro-Wilk test**						
3	W	Invalid input data	0.9641	Invalid input data	Invalid input data	Invalid input data	Invalid input data
4	P value		0.6360				
5	Passed normality test (alpha=0.05)?		Yes				
6	P value summary		ns				

Ordinary one-way ANOVA<br>ANOVA results						
1	**ANOVA summary**					
2	F	9.753				
3	P value	0.0007				
4	P value summary	***				
5	Significant diff. among means (P < 0.05)?	Yes				
6	R square	0.8025				
7						
8	**Brown-Forsythe test**					
9	F (DFn, DFd)	2.996 (5, 12)				
10	P value	0.0554				
11	P value summary	ns				
12	Are SDs significantly different (P < 0.05)?	No				
13						
14	**ANOVA table**	**SS**	**DF**	**MS**	**F (DFn, DFd)**	**P value**
15	Treatment (between columns)	0.9702	5	0.1940	F (5, 12) = 9.753	P=0.0007
16	Residual (within columns)	0.2387	12	0.01990		
18	Total	1.209	17			

Ordinary one-way ANOVA<br>Multiple comparisons						
1	**Uncorrected Fisher's LSD**	**Mean Diff.**	**95.00% CI of diff.**	**Significant?**	**Summary**	**Individual P Value**
2	**HS** vs. **IFN-I (IFNα)**	-0.6230	-0.8739 to -0.3720	Yes	***	0.0002
3	**HS** vs. **IFN-II (IFNγ)**	0.000	-0.2509 to 0.2509	No	ns	>0.9999
4	**HS** vs. **IFN-III (IFNλ)**	0.000	-0.2509 to 0.2509	No	ns	>0.9999
5	**FBS** vs. **IL27**	0.000	-0.2509 to 0.2509	No	ns	>0.9999

AXL TPM DATA

Normality and Lognormality Tests		A	B	C	D	E	F
		**HS**	**IFN-I (IFNα)**	**IFN-II (IFNγ)**	**IFN-III (IFNλ)**	**FBS**	**IL27**
1	**Test for normal distribution**						
2	**Shapiro-Wilk test**						
3	W	0.9998	0.9703	0.7922	0.9934	0.7609	0.9505
4	P value	0.9715	0.6694	0.0960	0.8443	0.0242	0.5714
5	Passed normality test (alpha=0.05)?	Yes	Yes	Yes	Yes	No	Yes
6	P value summary	ns	ns	ns	ns	*	ns

**Ordinary one-way ANOVA** ANOVA results						
1	**ANOVA summary**					
2	F	982.9				
3	P value	<0.0001				
4	P value summary	****				
5	Significant diff. among means (P < 0.05)?	Yes				
6	R square	0.9976				
7						
8	**Brown-Forsythe test**					
9	F (DFn, DFd)	1.487 (5, 12)				
10	P value	0.2649				
11	P value summary	ns				
12	Are SDs significantly different (P < 0.05)?	No				
13						
14	**ANOVA table**	**SS**	**DF**	**MS**	**F (DFn, DFd)**	**P value**
15	Treatment (between columns)	654.9	5	131.0	F (5, 12) = 982.9	P<0.0001
16	Residual (within columns)	1.599	12	0.1333		
18	Total	656.5	17			

**Ordinary one-way ANOVA** Multiple comparisons						
1	**Uncorrected Fisher's LSD**	**Mean Diff.**	**95.00% CI of diff.**	**Significant?**	**Summary**	**Individual P Value**
2	**HS** vs. **IFN-I (IFNα)**	-16.64	-17.29 to -15.99	Yes	****	<0.0001
3	**HS** vs. **IFN-II (IFNγ)**	-0.3238	-0.9732 to 0.3256	No	ns	0.2986
4	**HS** vs. **IFN-III (IFNλ)**	-5.767	-6.416 to -5.118	Yes	****	<0.0001
5	**FBS** vs. **IL27**	0.1409	-0.5085 to 0.7903	No	ns	0.6448

CD38 TPM DATA

Normality and Lognormality Tests		A	B	C	D	E	F
		**HS**	**IFN-I (IFNα)**	**IFN-II (IFNγ)**	**IFN-III (IFNλ)**	**FBS**	**IL27**
1	**Test for normal distribution**						
2	**Shapiro-Wilk test**						
3	W	0.9174	0.9931	0.8935	0.8402	0.8036	0.9108
4	P value	0.4431	0.8416	0.3652	0.2145	0.1232	0.4208
5	Passed normality test (alpha=0.05)?	Yes	Yes	Yes	Yes	Yes	Yes
6	P value summary	ns	ns	ns	ns	ns	ns

**Ordinary one-way ANOVA**<br>ANOVA results						
1	**ANOVA summary**					
2	F	830.6				
3	P value	<0.0001				
4	P value summary	****				
5	Significant diff. among means (P < 0.05)?	Yes				
6	R square	0.9971				
7						
8	**Brown-Forsythe test**					
9	F (DFn, DFd)	2.315 (5, 12)				
10	P value	0.1084				
11	P value summary	ns				
12	Are SDs significantly different (P < 0.05)?	No				
13						
14	**ANOVA table**	**SS**	**DF**	**MS**	**F (DFn, DFd)**	**P value**
15	Treatment (between columns)	221577	5	44315	F (5, 12) = 830.6	P<0.0001
16	Residual (within columns)	640.3	12	53.36		
18	Total	222217	17			

**Ordinary one-way ANOVA**<br>Multiple comparisons						
1	**Uncorrected Fisher's LSD**	**Mean Diff.**	**95.00% CI of diff.**	**Significant?**	**Summary**	**Individual P Value**
2	**HS** vs. **IFN-I (IFNα)**	-199.7	-212.7 to -186.7	Yes	****	<0.0001
3	**HS** vs. **IFN-II (IFNγ)**	-269.0	-282.0 to -256.0	Yes	****	<0.0001
4	**HS** vs. **IFN-III (IFNλ)**	-4.684	-17.68 to -4.310	No	*	0.0474
5	**FBS** vs. **IL27**	-8.255	-21.25 to -4.740	Yes	*	0.0191

SIGLEC1 TPM DATA

Normality and Lognormality Tests		A	B	C	D	E	F
		HS	IFN-I (IFNα)	IFN-II (IFNγ)	IFN-III (IFNλ)	FBS	IL27
1	Test for normal distribution						
2	Shapiro-Wilk test						
3	W	0.9062	0.9708	0.7717	0.8470	0.9491	0.9557
4	P value	0.4054	0.6719	0.0487	0.2323	0.5652	0.5952
5	Passed normality test (alpha=0.05)?	Yes	Yes	No	Yes	Yes	Yes
6	P value summary	ns	ns	*	ns	ns	ns

Ordinary one-way ANOVA<br>ANOVA results						
1	ANOVA summary					
2	F	462.7				
3	P value	<0.0001				
4	P value summary	****				
5	Significant diff. among means (P < 0.05)?	Yes				
6	R square	0.9948				
7						
8	Brown-Forsythe test					
9	F (DFn, DFd)	0.8758 (5, 12)				
10	P value	0.5256				
11	P value summary	ns				
12	Are SDs significantly different (P < 0.05)?	No				
13						
14	ANOVA table	SS	DF	MS	F (DFn, DFd)	P value
15	Treatment (between columns)	160845	5	32169	F (5, 12) = 462.7	P<0.0001
16	Residual (within columns)	834.3	12	69.52		
18	Total	161680	17			

Ordinary one-way ANOVA<br>Multiple comparisons						
1	Uncorrected Fisher's LSD	Mean Diff.	95.00% CI of diff.	Significant?	Summary	Individual P Value
2	**HS** vs. **IFN-I (IFNα)**	-265.0	-279.9 to -250.2	Yes	****	<0.0001
3	**HS** vs. **IFN-II (IFNγ)**	-56.72	-71.55 to -41.88	Yes	****	<0.0001
4	**HS** vs. **IFN-III (IFNλ)**	-128.7	-143.6 to -113.9	Yes	****	<0.0001
5	**FBS** vs. **IL27**	-21.11	-35.94 to -6.273	Yes	**	0.0092

SLAMF7 TPM DATA

Normality and Lognormality Tests		A	B	C	D	E	F
		**HS**	**IFN-I (IFNα)**	**IFN-II (IFNγ)**	**IFN-III (IFNλ)**	**FBS**	**IL27**
1	**Test for normal distribution**						
2	**Shapiro-Wilk test**						
3	W	0.9988	0.9920	0.9595	0.9996	0.9448	0.9899
4	P value	0.9348	0.8287	0.6129	0.9631	0.5472	0.8075
5	Passed normality test (alpha=0.05)?	Yes	Yes	Yes	Yes	Yes	Yes
6	P value summary	ns	ns	ns	ns	ns	ns

**Ordinary one-way ANOVA** ANOVA results						
1	**ANOVA summary**					
2	F	932.9				
3	P value	<0.0001				
4	P value summary	****				
5	Significant diff. among means (P < 0.05)?	Yes				
6	R square	0.9974				
7						
8	**Brown-Forsythe test**					
9	F (DFn, DFd)	1.971 (5, 12)				
10	P value	0.1556				
11	P value summary	ns				
12	Are SDs significantly different (P < 0.05)?	No				
13						
14	**ANOVA table**	**SS**	**DF**	**MS**	**F (DFn, DFd)**	**P value**
15	Treatment (between columns)	15806079	5	3161216	F (5, 12) = 932.9	P<0.0001
16	Residual (within columns)	40661	12	3388		
18	Total	15846741	17			

**Ordinary one-way ANOVA** Multiple comparisons						
1	**Uncorrected Fisher's LSD**	**Mean Diff.**	**95.00% CI of diff.**	**Significant?**	**Summary**	**Individual P Value**
2	**HS** vs. **IFN-I (IFNα)**	-1132	-1235 to -1028	Yes	****	<0.0001
3	**HS** vs. **IFN-II (IFNγ)**	-2746	-2850 to -2643	Yes	****	<0.0001
4	**HS** vs. **IFN-III (IFNλ)**	-318.0	-421.5 to -214.4	Yes	****	<0.0001
5	**FBS** vs. **IL27**	-355.1	-458.7 to -251.6	Yes	****	<0.0001

CD40 TPM DATA

Normality and Lognormality Tests		A<br>HS	B<br>IFN-I (IFNα)	C<br>IFN-II (IFNy)	D<br>IFN-III (IFNλ)	E<br>FBS	F<br>IL27
1	**Test for normal distribution**						
2	**Shapiro-Wilk test**						
3	W	0.9152	0.8373	0.7784	0.7811	0.9205	0.8587
4	P value	0.4358	0.2071	0.0639	0.0702	0.4542	0.2641
5	Passed normality test (alpha=0.05)?	Yes	Yes	Yes	Yes	Yes	Yes
6	P value summary	ns	ns	ns	ns	ns	ns

Ordinary one-way ANOVA
ANOVA results

1	**ANOVA summary**					
2	F	1030				
3	P value	<0.0001				
4	P value summary	****				
5	Significant diff. among means (P < 0.05)?	Yes				
6	R square	0.9977				
7						
8	**Brown-Forsythe test**					
9	F (DFn, DFd)	0.7741 (5, 12)				
10	P value	0.5865				
11	P value summary	ns				
12	Are SDs significantly different (P < 0.05)?	No				
13						
14	**ANOVA table**	**SS**	**DF**	**MS**	**F (DFn, DFd)**	**P value**
15	Treatment (between columns)	175957	5	35191	F (5, 12) = 1030	P<0.0001
16	Residual (within columns)	410.1	12	34.18		
18	Total	176367	17			

Ordinary one-way ANOVA
Multiple comparisons

1	**Uncorrected Fisher's LSD**	**Mean Diff.**	**95.00% CI of diff.**	**Significant?**	**Summary**	**Individual P Value**
2	**HS** vs. **IFN-I (IFNα)**	-126.7	-137.1 to -116.3	Yes	****	<0.0001
3	**HS** vs. **IFN-II (IFNy)**	-262.9	-273.3 to -252.5	Yes	****	<0.0001
4	**HS** vs. **IFN-III (IFNλ)**	-22.35	-32.75 to -11.95	Yes	***	0.0005
5	**FBS** vs. **IL27**	-51.63	-62.04 to -41.23	Yes	****	<0.0001

CD80 TPM DATA

	Normality and Lognormality Tests	A	B	C	D	E	F
		**HS**	**IFN-I (IFNα)**	**IFN-II (IFNγ)**	**IFN-III (IFNλ)**	**FBS**	**IL27**
1	**Test for normal distribution**						
2	**Shapiro-Wilk test**						
3	W	0.8534	0.9486	0.9979	0.7923	0.9319	0.9211
4	P value	0.2495	0.5632	0.9133	0.0963	0.4957	0.4564
5	Passed normality test (alpha=0.05)?	Yes	Yes	Yes	Yes	Yes	Yes
6	P value summary	ns	ns	ns	ns	ns	ns

	Ordinary one-way ANOVA<br>ANOVA results					
1	**ANOVA summary**					
2	F	773.8				
3	P value	<0.0001				
4	P value summary	****				
5	Significant diff. among means (P < 0.05)?	Yes				
6	R square	0.9969				
7						
8	**Brown-Forsythe test**					
9	F (DFn, DFd)	0.7432 (5, 12)				
10	P value	0.6060				
11	P value summary	ns				
12	Are SDs significantly different (P < 0.05)?	No				
13						
14	**ANOVA table**	**SS**	**DF**	**MS**	**F (DFn, DFd)**	**P value**
15	Treatment (between columns)	2018	5	403.6	F (5, 12) = 773.8	P<0.0001
16	Residual (within columns)	6.260	12	0.5216		
18	Total	2024	17			

	Ordinary one-way ANOVA<br>Multiple comparisons					
1	**Uncorrected Fisher's LSD**	**Mean Diff.**	**95.00% CI of diff.**	**Significant?**	**Summary**	**Individual P Value**
2	**HS** vs. **IFN-I (IFNα)**	-27.47	-28.76 to -26.19	Yes	****	<0.0001
3	**HS** vs. **IFN-II (IFNγ)**	-10.26	-11.54 to -8.971	Yes	****	<0.0001
4	**HS** vs. **IFN-III (IFNλ)**	-3.882	-5.167 to -2.597	Yes	****	<0.0001
5	**FBS** vs. **IL27**	0.05296	-1.232 to 1.338	No	ns	0.9299

CD86 TPM DATA

	Normality and Lognormality Tests	A<br>HS	B<br>IFN-I (IFNα)	C<br>IFN-II (IFNγ)	D<br>IFN-III (IFNλ)	E<br>FBS	F<br>IL27
1	**Test for normal distribution**						
2	**Shapiro-Wilk test**						
3	W	0.8787	0.9976	0.9062	0.9876	0.9869	0.9981
4	P value	0.3205	0.9069	0.4055	0.7869	0.7811	0.9173
5	Passed normality test (alpha=0.05)?	Yes	Yes	Yes	Yes	Yes	Yes
6	P value summary	ns	ns	ns	ns	ns	ns

	Ordinary one-way ANOVA<br>ANOVA results					
1	**ANOVA summary**					
2	F	236.4				
3	P value	<0.0001				
4	P value summary	****				
5	Significant diff. among means (P < 0.05)?	Yes				
6	R square	0.9899				
7						
8	**Brown-Forsythe test**					
9	F (DFn, DFd)	0.8694 (5, 12)				
10	P value	0.5293				
11	P value summary	ns				
12	Are SDs significantly different (P < 0.05)?	No				
13						
14	**ANOVA table**	**SS**	**DF**	**MS**	**F (DFn, DFd)**	**P value**
15	Treatment (between columns)	238758	5	47752	F (5, 12) = 236.4	P<0.0001
16	Residual (within columns)	2424	12	202.0		
18	Total	241182	17			

	Ordinary one-way ANOVA<br>Multiple comparisons					
1	**Uncorrected Fisher's LSD**	**Mean Diff.**	**95.00% CI of diff.**	**Significant?**	**Summary**	**Individual P Value**
2	**HS** vs. **IFN-I (IFNα)**	-191.2	-216.5 to -165.9	Yes	****	<0.0001
3	**HS** vs. **IFN-II (IFNγ)**	-283.9	-309.2 to -258.6	Yes	****	<0.0001
4	**HS** vs. **IFN-III (IFNλ)**	-37.81	-63.10 to -12.52	Yes	**	0.0069
5	**FBS** vs. **IL27**	-41.94	-67.22 to -16.65	Yes	**	0.0036

PD-L1 TPM DATA

Normality and Lognormality Tests		A	B	C	D	E	F
		**HS**	**IFN-I (IFNα)**	**IFN-II (IFNγ)**	**IFN-III (IFNλ)**	**FBS**	**IL27**
1	**Test for normal distribution**						
2	**Shapiro-Wilk test**						
3	W	0.9233	0.9492	0.8928	0.9776	0.9488	0.8048
4	P value	0.4640	0.5659	0.3630	0.7133	0.5642	0.1261
5	Passed normality test (alpha=0.05)?	Yes	Yes	Yes	Yes	Yes	Yes
6	P value summary	ns	ns	ns	ns	ns	ns

**Ordinary one-way ANOVA**<br>ANOVA results						
1	**ANOVA summary**					
2	F	858.8				
3	P value	<0.0001				
4	P value summary	****				
5	Significant diff. among means (P < 0.05)?	Yes				
6	R square	0.9972				
7						
8	**Brown-Forsythe test**					
9	F (DFn, DFd)	1.134 (5, 12)				
10	P value	0.3943				
11	P value summary	ns				
12	Are SDs significantly different (P < 0.05)?	No				
13						
14	**ANOVA table**	**SS**	**DF**	**MS**	**F (DFn, DFd)**	**P value**
15	Treatment (between columns)	394424	5	78885	F (5, 12) = 858.8	P<0.0001
16	Residual (within columns)	1102	12	91.86		
18	Total	395526	17			

**Ordinary one-way ANOVA**<br>Multiple comparisons						
1	**Uncorrected Fisher's LSD**	**Mean Diff.**	**95.00% CI of diff.**	**Significant?**	**Summary**	**Individual P Value**
2	**HS** vs. **IFN-I (IFNα)**	-115.8	-132.8 to -98.72	Yes	****	<0.0001
3	**HS** vs. **IFN-II (IFNγ)**	-405.7	-422.7 to -388.6	Yes	****	<0.0001
4	**HS** vs. **IFN-III (IFNλ)**	-26.78	-43.83 to -9.730	Yes	**	0.0051
5	**FBS** vs. **IL27**	-37.59	-54.64 to -20.54	Yes	***	0.0004

LMP2 TPM DATA

	Normality and Lognormality Tests	A	B	C	D	E	F
		HS	IFN-I (IFNα)	IFN-II (IFNγ)	IFN-III (IFNλ)	FBS	IL27
1	**Test for normal distribution**						
2	**Shapiro-Wilk test**						
3	W	0.8821	0.8661	0.9973	0.8579	0.8572	0.8189
4	P value	0.3306	0.2844	0.9015	0.2619	0.2599	0.1605
5	Passed normality test (alpha=0.05)?	Yes	Yes	Yes	Yes	Yes	Yes
6	P value summary	ns	ns	ns	ns	ns	ns

	Ordinary one-way ANOVA<br>ANOVA results					
1	**ANOVA summary**					
2	F	535.1				
3	P value	<0.0001				
4	P value summary	****				
5	Significant diff. among means (P < 0.05)?	Yes				
6	R square	0.9955				
7						
8	**Brown-Forsythe test**					
9	F (DFn, DFd)	0.4840 (5, 12)				
10	P value	0.7819				
11	P value summary	ns				
12	Are SDs significantly different (P < 0.05)?	No				
13						
14	**ANOVA table**	**SS**	**DF**	**MS**	**F (DFn, DFd)**	**P value**
15	Treatment (between columns)	5278	5	1056	F (5, 12) = 535.1	P<0.0001
16	Residual (within columns)	23.67	12	1.973		
18	Total	5301	17			

	Ordinary one-way ANOVA<br>Multiple comparisons					
1	**Uncorrected Fisher's LSD**	**Mean Diff.**	**95.00% CI of diff.**	**Significant?**	**Summary**	**Individual P Value**
2	**HS** vs. **IFN-I (IFNα)**	-36.67	-39.16 to -34.17	Yes	****	<0.0001
3	**HS** vs. **IFN-II (IFNγ)**	-46.29	-48.79 to -43.80	Yes	****	<0.0001
4	**HS** vs. **IFN-III (IFNλ)**	-16.81	-19.31 to -14.31	Yes	****	<0.0001
5	**FBS** vs. **IL27**	-16.74	-19.24 to -14.25	Yes	****	<0.0001

TAP1 TPM DATA

Normality and Lognormality Tests		A	B	C	D	E	F
		**HS**	**IFN-I (IFNα)**	**IFN-II (IFNγ)**	**IFN-III (IFNλ)**	**FBS**	**IL27**
1	**Test for normal distribution**						
2	**Shapiro-Wilk test**						
3	W	0.8419	0.9970	0.7978	0.9733	0.8517	0.9300
4	P value	0.2190	0.8949	0.1092	0.6865	0.2451	0.4888
5	Passed normality test (alpha=0.05)?	Yes	Yes	Yes	Yes	Yes	Yes
6	P value summary	ns	ns	ns	ns	ns	ns

**Ordinary one-way ANOVA** ANOVA results						
1	**ANOVA summary**					
2	F	1330				
3	P value	<0.0001				
4	P value summary	****				
5	Significant diff. among means (P < 0.05)?	Yes				
6	R square	0.9982				
7						
8	**Brown-Forsythe test**					
9	F (DFn, DFd)	0.6160 (5, 12)				
10	P value	0.6903				
11	P value summary	ns				
12	Are SDs significantly different (P < 0.05)?	No				
13						
14	**ANOVA table**	**SS**	**DF**	**MS**	**F (DFn, DFd)**	**P value**
15	Treatment (between columns)	72725	5	14545	F (5, 12) = 1330	P<0.0001
16	Residual (within columns)	131.2	12	10.93		
18	Total	72856	17			

**Ordinary one-way ANOVA** Multiple comparisons						
1	**Uncorrected Fisher's LSD**	**Mean Diff.**	**95.00% CI of diff.**	**Significant?**	**Summary**	**Individual P Value**
2	**HS** vs. **IFN-I (IFNα)**	-96.20	-102.1 to -90.32	Yes	****	<0.0001
3	**HS** vs. **IFN-II (IFNγ)**	-184.3	-190.2 to -178.4	Yes	****	<0.0001
4	**HS** vs. **IFN-III (IFNλ)**	-40.97	-46.85 to -35.09	Yes	****	<0.0001
5	**FBS** vs. **IL27**	-55.46	-61.34 to -49.57	Yes	****	<0.0001

HLA-B TPM DATA

Normality and Lognormality Tests		A<br>HS	B<br>IFN-I (IFNα)	C<br>IFN-II (IFNγ)	D<br>IFN-III (IFNλ)	E<br>FBS	F<br>IL27
1	**Test for normal distribution**						
2	**Shapiro-Wilk test**						
3	W	0.9743	0.9372	0.9421	0.9795	0.9946	0.9952
4	P value	0.6926	0.5163	0.5357	0.7255	0.8597	0.8672
5	Passed normality test (alpha=0.05)?	Yes	Yes	Yes	Yes	Yes	Yes
6	P value summary	ns	ns	ns	ns	ns	ns

**Ordinary one-way ANOVA**<br>ANOVA results						
1	**ANOVA summary**					
2	F	42.02				
3	P value	<0.0001				
4	P value summary	****				
5	Significant diff. among means (P < 0.05)?	Yes				
6	R square	0.9460				
7						
8	**Brown-Forsythe test**					
9	F (DFn, DFd)	2.194 (5, 12)				
10	P value	0.1229				
11	P value summary	ns				
12	Are SDs significantly different (P < 0.05)?	No				
13						
14	**ANOVA table**	**SS**	**DF**	**MS**	**F (DFn, DFd)**	**P value**
15	Treatment (between columns)	376491	5	75298	F (5, 12) = 42.02	P<0.0001
16	Residual (within columns)	21504	12	1792		
18	Total	397995	17			

**Ordinary one-way ANOVA**<br>Multiple comparisons						
1	**Uncorrected Fisher's LSD**	**Mean Diff.**	**95.00% CI of diff.**	**Significant?**	**Summary**	**Individual P Value**
2	**HS** vs. **IFN-I (IFNα)**	-250.0	-325.3 to -174.7	Yes	****	<0.0001
3	**HS** vs. **IFN-II (IFNγ)**	-245.3	-320.6 to -170.0	Yes	****	<0.0001
4	**HS** vs. **IFN-III (IFNλ)**	-34.77	-110.1 to 40.54	No	ns	0.3343
5	**FBS** vs. **IL27**	-254.6	-329.9 to -179.3	Yes	****	<0.0001

B2M TPM DATA							
Normality and Lognormality Tests		A	B	C	D	E	F
		HS	IFN-I (IFNα)	IFN-II (IFNγ)	IFN-III (IFNλ)	FBS	IL27
1	**Test for normal distribution**						
2	**Shapiro-Wilk test**						
3	W	0.9657	0.9902	0.8907	0.9897	0.8363	0.7854
4	P value	0.6441	0.8109	0.3564	0.8059	0.2045	0.0801
5	Passed normality test (alpha=0.05)?	Yes	Yes	Yes	Yes	Yes	Yes
6	P value summary	ns	ns	ns	ns	ns	ns

**Ordinary one-way ANOVA**<br>ANOVA results						
1	**ANOVA summary**					
2	F	78.41				
3	P value	<0.0001				
4	P value summary	****				
5	Significant diff. among means (P < 0.05)?	Yes				
6	R square	0.9703				
7						
8	**Brown-Forsythe test**					
9	F (DFn, DFd)	0.5411 (5, 12)				
10	P value	0.7421				
11	P value summary	ns				
12	Are SDs significantly different (P < 0.05)?	No				
13						
14	**ANOVA table**	**SS**	**DF**	**MS**	**F (DFn, DFd)**	**P value**
15	Treatment (between columns)	114268195	5	22853639	F (5, 12) = 78.41	P<0.0001
16	Residual (within columns)	3497742	12	291479		
18	Total	117765937	17			

**Ordinary one-way ANOVA**<br>Multiple comparisons						
1	**Uncorrected Fisher's LSD**	**Mean Diff.**	**95.00% CI of diff.**	**Significant?**	**Summary**	**Individual P Value**
2	**HS** vs. **IFN-I (IFNα)**	-3802	-4763 to -2842	Yes	****	<0.0001
3	**HS** vs. **IFN-II (IFNγ)**	-5299	-6259 to -4338	Yes	****	<0.0001
4	**HS** vs. **IFN-III (IFNλ)**	-486.6	-1447 to 473.8	No	ns	0.2913
5	**FBS** vs. **IL27**	-2918	-3878 to -1957	Yes	****	<0.0001

CIITA TPM DATA

Normality and Lognormality Tests		A<br>HS	B<br>IFN-I (IFNα)	C<br>IFN-II (IFNγ)	D<br>IFN-III (IFNλ)	E<br>FBS	F<br>IL27
1	**Test for normal distribution**						
2	**Shapiro-Wilk test**						
3	W	0.9810	0.8112	0.8380	0.9565	0.9442	0.9894
4	P value	0.7359	0.1414	0.2088	0.5989	0.5444	0.8028
5	Passed normality test (alpha=0.05)?	Yes	Yes	Yes	Yes	Yes	Yes
6	P value summary	ns	ns	ns	ns	ns	ns

**Ordinary one-way ANOVA**<br>ANOVA results						
1	**ANOVA summary**					
2	F	410.7				
3	P value	<0.0001				
4	P value summary	****				
5	Significant diff. among means (P < 0.05)?	Yes				
6	R square	0.9942				
7						
8	**Brown-Forsythe test**					
9	F (DFn, DFd)	1.548 (5, 12)				
10	P value	0.2473				
11	P value summary	ns				
12	Are SDs significantly different (P < 0.05)?	No				
13						
14	**ANOVA table**	**SS**	**DF**	**MS**	**F (DFn, DFd)**	**P value**
15	Treatment (between columns)	31177	5	6235	F (5, 12) = 410.7	P<0.0001
16	Residual (within columns)	182.2	12	15.18		
18	Total	31359	17			

**Ordinary one-way ANOVA**<br>Multiple comparisons						
1	**Uncorrected Fisher's LSD**	**Mean Diff.**	**95.00% CI of diff.**	**Significant?**	**Summary**	**Individual P Value**
2	**HS** vs. **IFN-I (IFNα)**	-3.925	-10.86 to 3.007	No	ns	0.2409
3	**HS** vs. **IFN-II (IFNγ)**	-112.4	-119.3 to -105.5	Yes	****	<0.0001
4	**HS** vs. **IFN-III (IFNλ)**	-0.7951	-7.727 to 6.137	No	ns	0.8069
5	**FBS** vs. **IL27**	-46.23	-53.16 to -39.30	Yes	****	<0.0001

CD74 TPM DATA

Normality and Lognormality Tests		A	B	C	D	E	F
		**HS**	**IFN-I (IFNα)**	**IFN-II (IFNγ)**	**IFN-III (IFNλ)**	**FBS**	**IL27**
1	**Test for normal distribution**						
2	**Shapiro-Wilk test**						
3	W	0.9896	0.9967	0.9999	0.9861	0.9641	0.9992
4	P value	0.8048	0.8910	0.9855	0.7741	0.6358	0.9452
5	Passed normality test (alpha=0.05)?	Yes	Yes	Yes	Yes	Yes	Yes
6	P value summary	ns	ns	ns	ns	ns	ns

**Ordinary one-way ANOVA**<br>ANOVA results						
1	**ANOVA summary**					
2	F	66.52				
3	P value	<0.0001				
4	P value summary	****				
5	Significant diff. among means (P < 0.05)?	Yes				
6	R square	0.9652				
7						
8	**Brown-Forsythe test**					
9	F (DFn, DFd)	1.875 (5, 12)				
10	P value	0.1727				
11	P value summary	ns				
12	Are SDs significantly different (P < 0.05)?	No				
13						
14	**ANOVA table**	**SS**	**DF**	**MS**	**F (DFn, DFd)**	**P value**
15	Treatment (between columns)	49194623	5	9838925	F (5, 12) = 66.52	P<0.0001
16	Residual (within columns)	1774801	12	147900		
18	Total	50969424	17			

**Ordinary one-way ANOVA**<br>Multiple comparisons						
1	**Uncorrected Fisher's LSD**	**Mean Diff.**	**95.00% CI of diff.**	**Significant?**	**Summary**	**Individual P Value**
2	**HS** vs. **IFN-I (IFNα)**	-744.6	-1429 to -60.49	Yes	*	0.0353
3	**HS** vs. **IFN-II (IFNγ)**	-4300	-4984 to -3616	Yes	****	<0.0001
4	**HS** vs. **IFN-III (IFNλ)**	-723.5	-1408 to -39.31	Yes	*	0.0399
5	**FBS** vs. **IL27**	-3071	-3755 to -2387	Yes	****	<0.0001

HLA-DOA TPM DATA

Normality and Lognormality Tests		A	B	C	D	E	F
		HS	IFN-I (IFNα)	IFN-II (IFNγ)	IFN-III (IFNλ)	FBS	IL27
1	**Test for normal distribution**						
2	**Shapiro-Wilk test**						
3	W	0.8882	0.9958	0.7907	0.9980	0.9989	0.9993
4	P value	0.3490	0.8764	0.0924	0.9145	0.9381	0.9496
5	Passed normality test (alpha=0.05)?	Yes	Yes	Yes	Yes	Yes	Yes
6	P value summary	ns	ns	ns	ns	ns	ns

**Ordinary one-way ANOVA** ANOVA results						
1	**ANOVA summary**					
2	F	15.70				
3	P value	<0.0001				
4	P value summary	****				
5	Significant diff. among means (P < 0.05)?	Yes				
6	R square	0.8674				
7						
8	**Brown-Forsythe test**					
9	F (DFn, DFd)	3.286 (5, 12)				
10	P value	0.0424				
11	P value summary	*				
12	Are SDs significantly different (P < 0.05)?	Yes				
13						
14	**ANOVA table**	**SS**	**DF**	**MS**	**F (DFn, DFd)**	**P value**
15	Treatment (between columns)	4753	5	950.5	F (5, 12) = 15.70	P<0.0001
16	Residual (within columns)	726.6	12	60.55		
18	Total	5479	17			

**Ordinary one-way ANOVA** Multiple comparisons									
1	**Uncorrected Fisher's LSD**	**Mean Diff.**	**95.00% CI of diff.**	**Significant?**	**Summary**	**Individual P Value**			
2	**HS** vs. **IFN-I (IFNα)**	1.576	-12.27 to 15.42	No	ns	0.8083	A-B		
3	**HS** vs. **IFN-II (IFNγ)**	-0.8035	-14.65 to 13.04	No	ns	0.9015	A-C		
4	**HS** vs. **IFN-III (IFNλ)**	-39.79	-53.63 to -25.95	Yes	****	<0.0001	A-D		
5	**FBS** vs. **IL27**	-25.43	-39.28 to -11.59	Yes	**	0.0018	E-F		

HLA-DRA TPM DATA

	Normality and Lognormality Tests	A	B	C	D	E	F
		**HS**	**IFN-I (IFNα)**	**IFN-II (IFNγ)**	**IFN-III (IFNλ)**	**FBS**	**IL27**
1	**Test for normal distribution**						
2	**Shapiro-Wilk test**						
3	W	0.8225	0.8715	0.7614	0.7715	0.7986	0.8285
4	P value	0.1695	0.2998	0.0253	0.0482	0.1111	0.1846
5	Passed normality test (alpha=0.05)?	Yes	Yes	No	No	Yes	Yes
	P value summary	ns	ns	*	*	ns	ns

	**Ordinary one-way ANOVA**<br>ANOVA results					
1	**ANOVA summary**					
2	F	299.0				
3	P value	<0.0001				
4	P value summary	****				
5	Significant diff. among means (P < 0.05)?	Yes				
6	R square	0.9920				
7						
8	**Brown-Forsythe test**					
9	F (DFn, DFd)	0.7459 (5, 12)				
10	P value	0.6043				
11	P value summary	ns				
12	Are SDs significantly different (P < 0.05)?	No				
13						
14	**ANOVA table**	**SS**	**DF**	**MS**	**F (DFn, DFd)**	**P value**
15	Treatment (between columns)	644857	5	128971	F (5, 12) = 299.0	P<0.0001
16	Residual (within columns)	5176	12	431.3		
18	Total	650033	17			

	**Ordinary one-way ANOVA**<br>Multiple comparisons					
1	**Uncorrected Fisher's LSD**	**Mean Diff.**	**95.00% CI of diff.**	**Significant?**	**Summary**	**Individual P Value**
2	**HS** vs. **IFN-I (IFNα)**	-39.81	-76.76 to -2.868	Yes	*	0.0368
3	**HS** vs. **IFN-II (IFNγ)**	-517.9	-554.9 to -481.0	Yes	****	<0.0001
4	**HS** vs. **IFN-III (IFNλ)**	-72.66	-109.6 to -35.72	Yes	**	0.0011
5	**FBS** vs. **IL27**	-279.9	-316.8 to -242.9	Yes	****	<0.0001

IFNE TPM DATA

Normality and Lognormality Tests		A	B	C	D	E	F
		HS	IFN-I (IFNα)	IFN-II (IFNγ)	IFN-III (IFNλ)	FBS	IL27
1	**Test for normal distribution**						
2	**Shapiro-Wilk test**						
3	W	Invalid input data	0.7955	Invalid input data	0.7581	Invalid input data	Invalid input data
4	P value		0.1038		0.0179		
5	Passed normality test (alpha=0.05)?		Yes		No		
6	P value summary		ns		*		

**Ordinary one-way ANOVA** ANOVA results						
1	**ANOVA summary**					
2	F	141.4				
3	P value	<0.0001				
4	P value summary	****				
5	Significant diff. among means (P < 0.05)?	Yes				
6	R square	0.9833				
7						
8	**Brown-Forsythe test**					
9	F (DFn, DFd)	0.8921 (5, 12)				
10	P value	0.5162				
11	P value summary	ns				
12	Are SDs significantly different (P < 0.05)?	No				
13						
14	**ANOVA table**	**SS**	**DF**	**MS**	**F (DFn, DFd)**	**P value**
15	Treatment (between columns)	0.8531	5	0.1706	F (5, 12) = 141.4	P<0.0001
16	Residual (within columns)	0.01448	12	0.001207		
18	Total	0.8675	17			

**Ordinary one-way ANOVA** Multiple comparisons						
1	**Uncorrected Fisher's LSD**	**Mean Diff.**	**95.00% CI of diff.**	**Significant?**	**Summary**	**Individual P Value**
2	**HS** vs. **IFN-I (IFNα)**	-0.5941	-0.6559 to -0.5323	Yes	****	<0.0001
3	**HS** vs. **IFN-II (IFNγ)**	0.000	-0.06180 to 0.06180	No	ns	>0.9999
4	**HS** vs. **IFN-III (IFNλ)**	-0.06957	-0.1314 to -0.007775	Yes	*	0.0304
5	**FBS** vs. **IL27**	0.000	-0.06180 to 0.06180	No	ns	>0.9999

IL27p28 TPM DATA

Normality and Lognormality Tests		A	B	C	D	E	F
		HS	IFN-I (IFNα)	IFN-II (IFNγ)	IFN-III (IFNλ)	FBS	IL27
1	**Test for normal distribution**						
2	**Shapiro-Wilk test**						
3	W	0.9804	0.9846	0.9523	0.9971	0.9943	0.9070
4	P value	0.7317	0.7627	0.5796	0.8969	0.8557	0.4082
5	Passed normality test (alpha=0.05)?	Yes	Yes	Yes	Yes	Yes	Yes
6	P value summary	ns	ns	ns	ns	ns	ns

**Ordinary one-way ANOVA** ANOVA results						
1	**ANOVA summary**					
2	F	208.9				
3	P value	<0.0001				
4	P value summary	****				
5	Significant diff. among means (P < 0.05)?	Yes				
6	R square	0.9886				
7						
8	**Brown-Forsythe test**					
9	F (DFn, DFd)	2.018 (5, 12)				
10	P value	0.1480				
11	P value summary	ns				
12	Are SDs significantly different (P < 0.05)?	No				
13						
14	**ANOVA table**	**SS**	**DF**	**MS**	**F (DFn, DFd)**	**P value**
15	Treatment (between columns)	2915	5	583.0	F (5, 12) = 208.9	P<0.0001
16	Residual (within columns)	33.49	12	2.791		
18	Total	2948	17			

**Ordinary one-way ANOVA** Multiple comparisons						
1	**Uncorrected Fisher's LSD**	**Mean Diff.**	**95.00% CI of diff.**	**Significant?**	**Summary**	**Individual P Value**
2	**HS** vs. **IFN-I (IFNα)**	-27.22	-30.20 to -24.25	Yes	****	<0.0001
3	**HS** vs. **IFN-II (IFNγ)**	-31.35	-34.32 to -28.38	Yes	****	<0.0001
4	**HS** vs. **IFN-III (IFNλ)**	-6.323	-9.295 to -3.352	Yes	***	0.0006
5	**FBS** vs. **IL27**	-1.194	-4.166 to 1.777	No	ns	0.3984

EBI3 TPM DATA							
Normality and Lognormality Tests		A	B	C	D	E	F
		HS	IFN-I (IFNα)	IFN-II (IFNγ)	IFN-III (IFNλ)	FBS	IL27
1	**Test for normal distribution**						
2	**Shapiro-Wilk test**						
3	W	0.8674	0.8033	0.9934	0.8672	0.9934	0.8523
4	P value	0.2882	0.1224	0.8447	0.2877	0.8447	0.2466
5	Passed normality test (alpha=0.05)?	Yes	Yes	Yes	Yes	Yes	Yes
6	P value summary	ns	ns	ns	ns	ns	ns

**Ordinary one-way ANOVA**<br>ANOVA results						
1	**ANOVA summary**					
2	F	15.56				
3	P value	<0.0001				
4	P value summary	****				
5	Significant diff. among means (P < 0.05)?	Yes				
6	R square	0.8664				
7						
8	**Brown-Forsythe test**					
9	F (DFn, DFd)	0.4151 (5, 12)				
10	P value	0.8295				
11	P value summary	ns				
12	Are SDs significantly different (P < 0.05)?	No				
13						
14	**ANOVA table**	**SS**	**DF**	**MS**	**F (DFn, DFd)**	**P value**
15	Treatment (between columns)	10.86	5	2.172	F (5, 12) = 15.56	P<0.0001
16	Residual (within columns)	1.675	12	0.1396		
18	Total	12.53	17			

**Ordinary one-way ANOVA**<br>Multiple comparisons						
1	**Uncorrected Fisher's LSD**	**Mean Diff.**	**95.00% CI of diff.**	**Significant?**	**Summary**	**Individual P Value**
2	**HS** vs. **IFN-I (IFNα)**	-0.06987	-0.7345 to 0.5947	No	ns	0.8227
3	**HS** vs. **IFN-II (IFNγ)**	-1.796	-2.461 to -1.132	Yes	****	<0.0001
4	**HS** vs. **IFN-III (IFNλ)**	0.04369	-0.6209 to 0.7083	No	ns	0.8885
5	**FBS** vs. **IL27**	-1.553	-2.217 to -0.8881	Yes	***	0.0003

ATCB TPM DATA

Normality and Lognormality Tests		A	B	C	D	E	F
		**HS**	**IFN-I (IFNα)**	**IFN-II (IFNγ)**	**IFN-III (IFNλ)**	**FBS**	**IL27**
1	**Test for normal distribution**						
2	**Shapiro-Wilk test**						
3	W	0.9988	0.8895	0.9530	0.7761	0.9622	0.7606
4	P value	0.9340	0.3527	0.5828	0.0586	0.6265	0.0235
5	Passed normality test (alpha=0.05)?	Yes	Yes	Yes	Yes	Yes	No
6	P value summary	ns	ns	ns	ns	ns	*

**Ordinary one-way ANOVA** ANOVA results						
1	**ANOVA summary**					
2	F	43.76				
3	P value	<0.0001				
4	P value summary	****				
5	Significant diff. among means (P < 0.05)?	Yes				
6	R square	0.9480				
7						
8	**Brown-Forsythe test**					
9	F (DFn, DFd)	0.4004 (5, 12)				
10	P value	0.8394				
11	P value summary	ns				
12	Are SDs significantly different (P < 0.05)?	No				
13						
14	**ANOVA table**	**SS**	**DF**	**MS**	**F (DFn, DFd)**	**P value**
15	Treatment (between columns)	18697911	5	3739582	F (5, 12) = 43.76	P<0.0001
16	Residual (within columns)	1025577	12	85465		
18	Total	19723488	17			

**Ordinary one-way ANOVA** Multiple comparisons						
1	**Uncorrected Fisher's LSD**	**Mean Diff.**	**95.00% CI of diff.**	**Significant?**	**Summary**	**Individual P Value**
2	**HS** vs. **IFN-I (IFNα)**	2018	1498 to 2538	Yes	****	<0.0001
3	**HS** vs. **IFN-II (IFNγ)**	1480	960.2 to 2000	Yes	****	<0.0001
4	**HS** vs. **IFN-III (IFNλ)**	571.9	51.85 to 1092	Yes	*	0.0338
5	**FBS** vs. **IL27**	-297.0	-817.1 to 223.1	No	ns	0.2372

TUBB TPM DATA							
Normality and Lognormality Tests		A	B	C	D	E	F
		HS	IFN-I (IFNα)	IFN-II (IFNγ)	IFN-III (IFNλ)	FBS	IL27
1	**Test for normal distribution**						
2	**Shapiro-Wilk test**						
3	W	0.8661	0.9518	0.7890	0.7635	0.9760	0.9274
4	P value	0.2844	0.5774	0.0885	0.0300	0.7032	0.4791
5	Passed normality test (alpha=0.05)?	Yes	Yes	Yes	No	Yes	Yes
6	P value summary	ns	ns	ns	*	ns	ns

Ordinary one-way ANOVA<br>ANOVA results						
1	**ANOVA summary**					
2	F	64.23				
3	P value	<0.0001				
4	P value summary	****				
5	Significant diff. among means (P < 0.05)?	Yes				
6	R square	0.9640				
7						
8	**Brown-Forsythe test**					
9	F (DFn, DFd)	1.457 (5, 12)				
10	P value	0.2741				
11	P value summary	ns				
12	Are SDs significantly different (P < 0.05)?	No				
13						
14	**ANOVA table**	**SS**	**DF**	**MS**	**F (DFn, DFd)**	**P value**
15	Treatment (between columns)	13266	5	2653	F (5, 12) = 64.23	P<0.0001
16	Residual (within columns)	495.7	12	41.31		
18	Total	13761	17			

Ordinary one-way ANOVA<br>Multiple comparisons						
1	**Uncorrected Fisher's LSD**	**Mean Diff.**	**95.00% CI of diff.**	**Significant?**	**Summary**	**Individual P Value**
2	**HS** vs. **IFN-I (IFNα)**	56.74	45.31 to 68.18	Yes	****	<0.0001
3	**HS** vs. **IFN-II (IFNγ)**	54.73	43.30 to 66.16	Yes	****	<0.0001
4	**HS** vs. **IFN-III (IFNλ)**	8.037	-3.397 to 19.47	No	ns	0.1516
5	**FBS** vs. **IL27**	15.26	3.831 to 26.70	Yes	*	0.0131

GAPDH TPM DATA

Normality and Lognormality Tests		A	B	C	D	E	F
		HS	IFN-I (IFNα)	IFN-II (IFNγ)	IFN-III (IFNλ)	FBS	IL27
1	**Test for normal distribution**						
2	**Shapiro-Wilk test**						
3	W	0.9579	0.9974	0.9938	0.8901	0.8414	0.9914
4	P value	0.6053	0.9019	0.8492	0.3547	0.2178	0.8231
5	Passed normality test (alpha=0.05)?	Yes	Yes	Yes	Yes	Yes	Yes
6	P value summary	ns	ns	ns	ns	ns	ns

**Ordinary one-way ANOVA** ANOVA results						
1	**ANOVA summary**					
2	F	29.19				
3	P value	<0.0001				
4	P value summary	****				
5	Significant diff. among means (P < 0.05)?	Yes				
6	R square	0.9240				
7						
8	**Brown-Forsythe test**					
9	F (DFn, DFd)	1.056 (5, 12)				
10	P value	0.4306				
11	P value summary	ns				
12	Are SDs significantly different (P < 0.05)?	No				
13						
14	**ANOVA table**	**SS**	**DF**	**MS**	**F (DFn, DFd)**	**P value**
15	Treatment (between columns)	8642193	5	1728439	F (5, 12) = 29.19	P<0.0001
16	Residual (within columns)	710677	12	59223		
18	Total	9352869	17			

**Ordinary one-way ANOVA** Multiple comparisons						
1	**Uncorrected Fisher's LSD**	**Mean Diff.**	**95.00% CI of diff.**	**Significant?**	**Summary**	**Individual P Value**
2	**HS** vs. **IFN-I (IFNα)**	1088	654.8 to 1521	Yes	***	0.0001
3	**HS** vs. **IFN-II (IFNγ)**	1021	588.0 to 1454	Yes	***	0.0002
4	**HS** vs. **IFN-III (IFNλ)**	-226.2	-659.1 to 206.7	No	ns	0.2772
5	**FBS** vs. **IL27**	260.4	-172.6 to 693.3	No	ns	0.2146

PGK1 TPM DATA							
Normality and Lognormality Tests		A	B	C	D	E	F
		HS	IFN-I (IFNα)	IFN-II (IFNy)	IFN-III (IFNλ)	FBS	IL27
1	**Test for normal distribution**						
2	**Shapiro-Wilk test**						
3	W	0.9980	0.8034	0.7798	0.7725	0.9665	0.8947
4	P value	0.9139	0.1227	0.0671	0.0503	0.6486	0.3688
5	Passed normality test (alpha=0.05)?	Yes	Yes	Yes	Yes	Yes	Yes
6	P value summary	ns	ns	ns	ns	ns	ns

Ordinary one-way ANOVA<br>ANOVA results						
1	**ANOVA summary**					
2	F	40.14				
3	P value	<0.0001				
4	P value summary	****				
5	Significant diff. among means (P < 0.05)?	Yes				
6	R square	0.9436				
7						
8	**Brown-Forsythe test**					
9	F (DFn, DFd)	0.5294 (5, 12)				
10	P value	0.7503				
11	P value summary	ns				
12	Are SDs significantly different (P < 0.05)?	No				
13						
14	**ANOVA table**	**SS**	**DF**	**MS**	**F (DFn, DFd)**	**P value**
15	Treatment (between columns)	156939	5	31388	F (5, 12) = 40.14	P<0.0001
16	Residual (within columns)	9384	12	782.0		
18	Total	166323	17			

Ordinary one-way ANOVA<br>Multiple comparisons						
1	**Uncorrected Fisher's LSD**	**Mean Diff.**	**95.00% CI of diff.**	**Significant?**	**Summary**	**Individual P Value**
2	**HS** vs. **IFN-I (IFNα)**	184.9	135.2 to 234.7	Yes	****	<0.0001
3	**HS** vs. **IFN-II (IFNy)**	132.9	83.17 to 182.7	Yes	****	<0.0001
4	**HS** vs. **IFN-III (IFNλ)**	-54.04	-103.8 to -4.291	Yes	*	0.0356
5	**FBS** vs. **IL27**	-50.88	-100.6 to -1.136	Yes	*	0.0457

CD14 TPM DATA							
Normality and Lognormality Tests		A	B	C	D	E	F
		HS	IFN-I (IFNα)	IFN-II (IFNγ)	IFN-III (IFNλ)	FBS	IL27
1	**Test for normal distribution**						
2	**Shapiro-Wilk test**						
3	W	0.8574	0.9920	0.7646	0.9998	0.9292	0.9872
4	P value	0.2604	0.8286	0.0325	0.9726	0.4857	0.7834
5	Passed normality test (alpha=0.05)?	Yes	Yes	No	Yes	Yes	Yes
6	P value summary	ns	ns	*	ns	ns	ns

Ordinary one-way ANOVA<br>ANOVA results						
1	**ANOVA summary**					
2	F	20.52				
3	P value	<0.0001				
4	P value summary	****				
5	Significant diff. among means (P < 0.05)?	Yes				
6	R square	0.8953				
7						
8	**Brown-Forsythe test**					
9	F (DFn, DFd)	0.7552 (5, 12)				
10	P value	0.5984				
11	P value summary	ns				
12	Are SDs significantly different (P < 0.05)?	No				
13						
14	**ANOVA table**	**SS**	**DF**	**MS**	**F (DFn, DFd)**	**P value**
15	Treatment (between columns)	50653	5	10131	F (5, 12) = 20.52	P<0.0001
16	Residual (within columns)	5924	12	493.6		
18	Total	56577	17			

Ordinary one-way ANOVA<br>Multiple comparisons						
1	**Uncorrected Fisher's LSD**	**Mean Diff.**	**95.00% CI of diff.**	**Significant?**	**Summary**	**Individual P Value**
2	**HS** vs. **IFN-I (IFNα)**	5.944	-33.58 to 45.47	No	ns	0.7488
3	**HS** vs. **IFN-II (IFNγ)**	-119.1	-158.7 to -79.62	Yes	****	<0.0001
4	**HS** vs. **IFN-III (IFNλ)**	-76.49	-116.0 to -36.96	Yes	**	0.0012
5	**FBS** vs. **IL27**	40.93	1.407 to 80.46	Yes	*	0.0435

CD16A TPM DATA

	Normality and Lognormality Tests	A	B	C	D	E	F
		HS	IFN-I (IFNα)	IFN-II (IFNγ)	IFN-III (IFNλ)	FBS	IL27
1	**Test for normal distribution**						
2	**Shapiro-Wilk test**						
3	W	0.8600	0.9809	0.9075	0.9502	0.9395	0.9941
4	P value	0.2675	0.7349	0.4096	0.5700	0.5254	0.8530
5	Passed normality test (alpha=0.05)?	Yes	Yes	Yes	Yes	Yes	Yes
6	P value summary	ns	ns	ns	ns	ns	ns

	**Ordinary one-way ANOVA** ANOVA results					
1	**ANOVA summary**					
2	F	23.52				
3	P value	<0.0001				
4	P value summary	****				
5	Significant diff. among means (P < 0.05)?	Yes				
6	R square	0.9074				
7						
8	**Brown-Forsythe test**					
9	F (DFn, DFd)	1.326 (5, 12)				
10	P value	0.3176				
11	P value summary	ns				
12	Are SDs significantly different (P < 0.05)?	No				
13						
14	**ANOVA table**	**SS**	**DF**	**MS**	**F (DFn, DFd)**	**P value**
15	Treatment (between columns)	5845	5	1169	F (5, 12) = 23.52	P<0.0001
16	Residual (within columns)	596.5	12	49.70		
18	Total	6442	17			

	**Ordinary one-way ANOVA** Multiple comparisons					
1	**Uncorrected Fisher's LSD**	**Mean Diff.**	**95.00% CI of diff.**	**Significant?**	**Summary**	**Individual P Value**
2	**HS** vs. **IFN-I (IFNα)**	-38.35	-50.89 to -25.81	Yes	****	<0.0001
3	**HS** vs. **IFN-II (IFNγ)**	7.546	-4.996 to 20.09	No	ns	0.2144
4	**HS** vs. **IFN-III (IFNλ)**	1.053	-11.49 to 13.59	No	ns	0.8580
5	**FBS** vs. **IL27**	-35.75	-48.29 to -23.21	Yes	****	<0.0001

CD11B TPM DATA

Normality and Lognormality Tests		A	B	C	D	E	F
		HS	IFN-I (IFNα)	IFN-II (IFNγ)	IFN-III (IFNλ)	FBS	IL27
1	Test for normal distribution						
2	Shapiro-Wilk test						
3	W	0.9458	0.9830	0.9450	0.8411	0.8983	0.8350
4	P value	0.5512	0.7502	0.5478	0.2171	0.3801	0.2011
5	Passed normality test (alpha=0.05)?	Yes	Yes	Yes	Yes	Yes	Yes
6	P value summary	ns	ns	ns	ns	ns	ns

Ordinary one-way ANOVA<br>ANOVA results						
1	ANOVA summary					
2	F	98.63				
3	P value	<0.0001				
4	P value summary	****				
5	Significant diff. among means (P < 0.05)?	Yes				
6	R square	0.9762				
7						
8	Brown-Forsythe test					
9	F (DFn, DFd)	0.7619 (5, 12)				
10	P value	0.5942				
11	P value summary	ns				
12	Are SDs significantly different (P < 0.05)?	No				
13						
14	ANOVA table	SS	DF	MS	F (DFn, DFd)	P value
15	Treatment (between columns)	458272	5	91654	F (5, 12) = 98.63	P<0.0001
16	Residual (within columns)	11152	12	929.3		
18	Total	469424	17			

Ordinary one-way ANOVA<br>Multiple comparisons						
1	Uncorrected Fisher's LSD	Mean Diff.	95.00% CI of diff.	Significant?	Summary	Individual P Value
2	**HS** vs. **IFN-I (IFNα)**	50.56	-3.674 to 104.8	No	ns	0.0650
3	**HS** vs. **IFN-II (IFNγ)**	-2.740	-56.97 to 51.49	No	ns	0.9142
4	**HS** vs. **IFN-III (IFNλ)**	71.39	17.16 to 125.6	Yes	*	0.0141
5	**FBS** vs. **IL27**	-31.07	-85.30 to 23.16	No	ns	0.2358

CD163 TPM DATA

Normality and Lognormality Tests		A HS	B IFN-I (IFNα)	C IFN-II (IFNγ)	D IFN-III (IFNλ)	E FBS	F IL27
1	**Test for normal distribution**						
2	**Shapiro-Wilk test**						
3	W	0.9139	0.9968	0.9827	0.9487	0.8441	0.7902
4	P value	0.4314	0.8913	0.7478	0.5637	0.2249	0.0912
5	Passed normality test (alpha=0.05)?	Yes	Yes	Yes	Yes	Yes	Yes
6	P value summary	ns	ns	ns	ns	ns	ns

Ordinary one-way ANOVA<br>ANOVA results						
1	**ANOVA summary**					
2	F	169.6				
3	P value	<0.0001				
4	P value summary	****				
5	Significant diff. among means (P < 0.05)?	Yes				
6	R square	0.9860				
7						
8	**Brown-Forsythe test**					
9	F (DFn, DFd)	0.4779 (5, 12)				
10	P value	0.7862				
11	P value summary	ns				
12	Are SDs significantly different (P < 0.05)?	No				
13						
14	**ANOVA table**	**SS**	**DF**	**MS**	**F (DFn, DFd)**	**P value**
15	Treatment (between columns)	385506	5	77101	F (5, 12) = 169.6	P<0.0001
16	Residual (within columns)	5457	12	454.7		
18	Total	390963	17			

Ordinary one-way ANOVA<br>Multiple comparisons						
1	**Uncorrected Fisher's LSD**	**Mean Diff.**	**95.00% CI of diff.**	**Significant?**	**Summary**	**Individual P Value**
2	**HS** vs. **IFN-I (IFNα)**	-101.5	-139.5 to -63.61	Yes	****	<0.0001
3	**HS** vs. **IFN-II (IFNγ)**	130.8	92.91 to 168.8	Yes	****	<0.0001
4	**HS** vs. **IFN-III (IFNλ)**	-159.9	-197.9 to -122.0	Yes	****	<0.0001
5	**FBS** vs. **IL27**	83.21	45.27 to 121.1	Yes	***	0.0004

IFNAR1 TPM DATA

	Normality and Lognormality Tests	A	B	C	D	E	F
		HS	IFN-I (IFNα)	IFN-II (IFNγ)	IFN-III (IFNλ)	FBS	IL27
1	**Test for normal distribution**						
2	**Shapiro-Wilk test**						
3	W	0.9756	0.9556	0.9994	0.8425	0.9734	0.8852
4	P value	0.7002	0.5944	0.9540	0.2206	0.6871	0.3398
5	Passed normality test (alpha=0.05)?	Yes	Yes	Yes	Yes	Yes	Yes
6	P value summary	ns	ns	ns	ns	ns	ns

	**Ordinary one-way ANOVA**<br>ANOVA results					
1	**ANOVA summary**					
2	F	86.53				
3	P value	<0.0001				
4	P value summary	****				
5	Significant diff. among means (P < 0.05)?	Yes				
6	R square	0.9730				
7						
8	**Brown-Forsythe test**					
9	F (DFn, DFd)	0.1734 (5, 12)				
10	P value	0.9675				
11	P value summary	ns				
12	Are SDs significantly different (P < 0.05)?	No				
13						
14	**ANOVA table**	**SS**	**DF**	**MS**	**F (DFn, DFd)**	**P value**
15	Treatment (between columns)	9842	5	1968	F (5, 12) = 86.53	P<0.0001
16	Residual (within columns)	273.0	12	22.75		
18	Total	10115	17			

	**Ordinary one-way ANOVA**<br>Multiple comparisons					
1	**Uncorrected Fisher's LSD**	**Mean Diff.**	**95.00% CI of diff.**	**Significant?**	**Summary**	**Individual P Value**
2	**HS** vs. **IFN-I (IFNα)**	0.3710	-8.114 to 8.856	No	ns	0.9257
3	**HS** vs. **IFN-II (IFNγ)**	0.6785	-7.806 to 9.163	No	ns	0.8646
4	**HS** vs. **IFN-III (IFNλ)**	12.93	4.443 to 21.41	Yes	**	0.0061
5	**FBS** vs. **IL27**	8.792	0.3077 to 17.28	Yes	*	0.0434

IFNAR2 TPM DATA							
Normality and Lognormality Tests		A	B	C	D	E	F
		HS	IFN-I (IFNα)	IFN-II (IFNy)	IFN-III (IFNλ)	FBS	IL27
1	**Test for normal distribution**						
2	**Shapiro-Wilk test**						
3	W	0.9090	0.9398	0.8299	0.9578	0.9814	0.9884
4	P value	0.4146	0.5265	0.1880	0.6049	0.7384	0.7940
5	Passed normality test (alpha=0.05)?	Yes	Yes	Yes	Yes	Yes	Yes
6	P value summary	ns	ns	ns	ns	ns	ns

**Ordinary one-way ANOVA** ANOVA results						
1	**ANOVA summary**					
2	F	48.77				
3	P value	<0.0001				
4	P value summary	****				
5	Significant diff. among means (P < 0.05)?	Yes				
6	R square	0.9531				
7						
8	**Brown-Forsythe test**					
9	F (DFn, DFd)	0.9290 (5, 12)				
10	P value	0.4957				
11	P value summary	ns				
12	Are SDs significantly different (P < 0.05)?	No				
13						
14	**ANOVA table**	**SS**	**DF**	**MS**	**F (DFn, DFd)**	**P value**
15	Treatment (between columns)	3069	5	613.8	F (5, 12) = 48.77	P<0.0001
16	Residual (within columns)	151.0	12	12.58		
18	Total	3220	17			

**Ordinary one-way ANOVA** Multiple comparisons						
1	**Uncorrected Fisher's LSD**	**Mean Diff.**	**95.00% CI of diff.**	**Significant?**	**Summary**	**Individual P Value**
2	**HS** vs. **IFN-I (IFNα)**	-5.114	-11.42 to 1.197	No	ns	0.1029
3	**HS** vs. **IFN-II (IFNy)**	-36.81	-43.12 to -30.50	Yes	****	<0.0001
4	**HS** vs. **IFN-III (IFNλ)**	2.403	-3.908 to 8.714	No	ns	0.4230
5	**FBS** vs. **IL27**	-7.266	-13.58 to -0.9547	Yes	*	0.0275

IFNGR1 TPM DATA

Normality and Lognormality Tests		A<br>HS	B<br>IFN-I (IFNα)	C<br>IFN-II (IFNγ)	D<br>IFN-III (IFNλ)	E<br>FBS	F<br>IL27
1	**Test for normal distribution**						
2	**Shapiro-Wilk test**						
3	W	0.9554	0.9881	1.000	0.9434	0.9615	0.9701
4	P value	0.5937	0.7912	0.9880	0.5411	0.6228	0.6684
5	Passed normality test (alpha=0.05)?	Yes	Yes	Yes	Yes	Yes	Yes
6	P value summary	ns	ns	ns	ns	ns	ns

Ordinary one-way ANOVA<br>ANOVA results						
1	**ANOVA summary**					
2	F	41.77				
3	P value	<0.0001				
4	P value summary	****				
5	Significant diff. among means (P < 0.05)?	Yes				
6	R square	0.9457				
7						
8	**Brown-Forsythe test**					
9	F (DFn, DFd)	1.619 (5, 12)				
10	P value	0.2288				
11	P value summary	ns				
12	Are SDs significantly different (P < 0.05)?	No				
13						
14	**ANOVA table**	**SS**	**DF**	**MS**	**F (DFn, DFd)**	**P value**
15	Treatment (between columns)	73831	5	14766	F (5, 12) = 41.77	P<0.0001
16	Residual (within columns)	4242	12	353.5		
18	Total	78073	17			

Ordinary one-way ANOVA<br>Multiple comparisons						
1	**Uncorrected Fisher's LSD**	**Mean Diff.**	**95.00% CI of diff.**	**Significant?**	**Summary**	**Individual P Value**
2	**HS** vs. **IFN-I (IFNα)**	77.10	43.65 to 110.5	Yes	***	0.0003
3	**HS** vs. **IFN-II (IFNγ)**	86.82	53.38 to 120.3	Yes	***	0.0001
4	**HS** vs. **IFN-III (IFNλ)**	101.0	67.56 to 134.5	Yes	****	<0.0001
5	**FBS** vs. **IL27**	83.19	49.75 to 116.6	Yes	***	0.0002

IFNGR2 TPM DATA

Normality and Lognormality Tests		A	B	C	D	E	F
		**HS**	**IFN-I (IFNα)**	**IFN-II (IFNy)**	**IFN-III (IFNλ)**	**FBS**	**IL27**
1	**Test for normal distribution**						
2	**Shapiro-Wilk test**						
3	W	0.9102	0.9994	0.9099	0.7652	0.8955	0.9981
4	P value	0.4186	0.9518	0.4179	0.0339	0.3713	0.9176
5	Passed normality test (alpha=0.05)?	Yes	Yes	Yes	No	Yes	Yes
6	P value summary	ns	ns	ns	*	ns	ns

**Ordinary one-way ANOVA** ANOVA results						
1	**ANOVA summary**					
2	F	3.775				
3	P value	0.0276				
4	P value summary	*				
5	Significant diff. among means (P < 0.05)?	Yes				
6	R square	0.6113				
7						
8	**Brown-Forsythe test**					
9	F (DFn, DFd)	1.864 (5, 12)				
10	P value	0.1748				
11	P value summary	ns				
12	Are SDs significantly different (P < 0.05)?	No				
13						
14	**ANOVA table**	**SS**	**DF**	**MS**	**F (DFn, DFd)**	**P value**
15	Treatment (between columns)	6613	5	1323	F (5, 12) = 3.775	P=0.0276
16	Residual (within columns)	4205	12	350.4		
18	Total	10818	17			

**Ordinary one-way ANOVA** Multiple comparisons						
1	**Uncorrected Fisher's LSD**	**Mean Diff.**	**95.00% CI of diff.**	**Significant?**	**Summary**	**Individual P Value**
2	**HS** vs. **IFN-I (IFNα)**	-17.51	-50.81 to 15.79	No	ns	0.2743
3	**HS** vs. **IFN-II (IFNy)**	-10.81	-44.11 to 22.49	No	ns	0.4930
4	**HS** vs. **IFN-III (IFNλ)**	-22.71	-56.01 to 10.59	No	ns	0.1631
5	**FBS** vs. **IL27**	-26.80	-60.10 to 6.501	No	ns	0.1050

IFNLR1 TPM DATA

Normality and Lognormality Tests		A	B	C	D	E	F
		HS	IFN-I (IFNα)	IFN-II (IFNγ)	IFN-III (IFNλ)	FBS	IL27
1	**Test for normal distribution**						
2	**Shapiro-Wilk test**						
3	W	0.8694	0.9979	1.000	0.8966	0.9789	0.9634
4	P value	0.2937	0.9131	0.9882	0.3747	0.7218	0.6325
5	Passed normality test (alpha=0.05)?	Yes	Yes	Yes	Yes	Yes	Yes
6	P value summary	ns	ns	ns	ns	ns	ns

Ordinary one-way ANOVA<br>ANOVA results						
1	**ANOVA summary**					
2	F	35.51				
3	P value	<0.0001				
4	P value summary	****				
5	Significant diff. among means (P < 0.05)?	Yes				
6	R square	0.9367				
7						
8	**Brown-Forsythe test**					
9	F (DFn, DFd)	1.732 (5, 12)				
10	P value	0.2018				
11	P value summary	ns				
12	Are SDs significantly different (P < 0.05)?	No				
13						
14	**ANOVA table**	**SS**	**DF**	**MS**	**F (DFn, DFd)**	**P value**
15	Treatment (between columns)	167.2	5	33.44	F (5, 12) = 35.51	P<0.0001
16	Residual (within columns)	11.30	12	0.9417		
18	Total	178.5	17			

Ordinary one-way ANOVA<br>Multiple comparisons						
1	**Uncorrected Fisher's LSD**	**Mean Diff.**	**95.00% CI of diff.**	**Significant?**	**Summary**	**Individual P Value**
2	**HS** vs. **IFN-I (IFNα)**	-7.123	-8.849 to -5.397	Yes	****	<0.0001
3	**HS** vs. **IFN-II (IFNγ)**	2.012	0.2861 to 3.739	Yes	*	0.0259
4	**HS** vs. **IFN-III (IFNλ)**	0.2577	-1.469 to 1.984	No	ns	0.7506
5	**FBS** vs. **IL27**	1.043	-0.6838 to 2.769	No	ns	0.2128

IL10RB TPM DATA							
Normality and Lognormality Tests		A	B	C	D	E	F
		HS	IFN-I (IFNα)	IFN-II (IFNγ)	IFN-III (IFNλ)	FBS	IL27
1	**Test for normal distribution**						
2	**Shapiro-Wilk test**						
3	W	0.7594	0.9678	0.9974	0.9981	0.8925	0.9537
4	P value	0.0208	0.6552	0.9028	0.9158	0.3621	0.5859
5	Passed normality test (alpha=0.05)?	No	Yes	Yes	Yes	Yes	Yes
6	P value summary	*	ns	ns	ns	ns	ns

Ordinary one-way ANOVA<br>ANOVA results						
1	**ANOVA summary**					
2	F	26.74				
3	P value	<0.0001				
4	P value summary	****				
5	Significant diff. among means (P < 0.05)?	Yes				
6	R square	0.9176				
7						
8	**Brown-Forsythe test**					
9	F (DFn, DFd)	1.019 (5, 12)				
10	P value	0.4486				
11	P value summary	ns				
12	Are SDs significantly different (P < 0.05)?	No				
13						
14	**ANOVA table**	**SS**	**DF**	**MS**	**F (DFn, DFd)**	**P value**
15	Treatment (between columns)	2189	5	437.7	F (5, 12) = 26.74	P<0.0001
16	Residual (within columns)	196.4	12	16.37		
18	Total	2385	17			

Ordinary one-way ANOVA<br>Multiple comparisons						
1	**Uncorrected Fisher's LSD**	**Mean Diff.**	**95.00% CI of diff.**	**Significant?**	**Summary**	**Individual P Value**
2	**HS** vs. **IFN-I (IFNα)**	-15.76	-22.95 to -8.558	Yes	***	0.0005
3	**HS** vs. **IFN-II (IFNγ)**	-14.26	-21.46 to -7.066	Yes	**	0.0010
4	**HS** vs. **IFN-III (IFNλ)**	2.440	-4.758 to 9.637	No	ns	0.4744
5	**FBS** vs. **IL27**	-1.787	-8.985 to 5.410	No	ns	0.5984

IL27R TPM DATA

Normality and Lognormality Tests		A	B	C	D	E	F
		HS	IFN-I (IFNα)	IFN-II (IFNγ)	IFN-III (IFNλ)	FBS	IL27
1	**Test for normal distribution**						
2	**Shapiro-Wilk test**						
3	W	0.8650	0.7880	0.9159	0.9737	0.9568	0.9738
4	P value	0.2814	0.0861	0.4379	0.6889	0.5999	0.6894
5	Passed normality test (alpha=0.05)?	Yes	Yes	Yes	Yes	Yes	Yes
6	P value summary	ns	ns	ns	ns	ns	ns

**Ordinary one-way ANOVA** ANOVA results						
1	**ANOVA summary**					
2	F	18.22				
3	P value	<0.0001				
4	P value summary	****				
5	Significant diff. among means (P < 0.05)?	Yes				
6	R square	0.8836				
7						
8	**Brown-Forsythe test**					
9	F (DFn, DFd)	1.213 (5, 12)				
10	P value	0.3607				
11	P value summary	ns				
12	Are SDs significantly different (P < 0.05)?	No				
13						
14	**ANOVA table**	**SS**	**DF**	**MS**	**F (DFn, DFd)**	**P value**
15	Treatment (between columns)	4131	5	826.3	F (5, 12) = 18.22	P<0.0001
16	Residual (within columns)	544.2	12	45.35		
18	Total	4676	17			

**Ordinary one-way ANOVA** Multiple comparisons						
1	**Uncorrected Fisher's LSD**	**Mean Diff.**	**95.00% CI of diff.**	**Significant?**	**Summary**	**Individual P Value**
2	**HS** vs. **IFN-I (IFNα)**	3.936	-8.044 to 15.92	No	ns	0.4878
3	**HS** vs. **IFN-II (IFNγ)**	26.28	14.30 to 38.26	Yes	***	0.0004
4	**HS** vs. **IFN-III (IFNλ)**	-2.771	-14.75 to 9.209	No	ns	0.6234
5	**FBS** vs. **IL27**	6.650	-5.330 to 18.63	No	ns	0.2498

gp130 TPM DATA

	Normality and Lognormality Tests	A	B	C	D	E	F
		HS	IFN-I (IFNα)	IFN-II (IFNγ)	IFN-III (IFNλ)	FBS	IL27
1	**Test for normal distribution**						
2	**Shapiro-Wilk test**						
3	W	0.8436	0.8470	0.7740	0.9836	0.9815	0.7979
4	P value	0.2234	0.2324	0.0539	0.7550	0.7398	0.1094
5	Passed normality test (alpha=0.05)?	Yes	Yes	Yes	Yes	Yes	Yes
6	P value summary	ns	ns	ns	ns	ns	ns

	**Ordinary one-way ANOVA**<br>ANOVA results					
1	**ANOVA summary**					
2	F	92.32				
3	P value	<0.0001				
4	P value summary	****				
5	Significant diff. among means (P < 0.05)?	Yes				
6	R square	0.9747				
7						
8	**Brown-Forsythe test**					
9	F (DFn, DFd)	0.3868 (5, 12)				
10	P value	0.8485				
11	P value summary	ns				
12	Are SDs significantly different (P < 0.05)?	No				
13						
14	**ANOVA table**	**SS**	**DF**	**MS**	**F (DFn, DFd)**	**P value**
15	Treatment (between columns)	1046	5	209.1	F (5, 12) = 92.32	P<0.0001
16	Residual (within columns)	27.18	12	2.265		
18	Total	1073	17			

	**Ordinary one-way ANOVA**<br>Multiple comparisons					
1	**Uncorrected Fisher's LSD**	**Mean Diff.**	**95.00% CI of diff.**	**Significant?**	**Summary**	**Individual P Value**
2	**HS** vs. **IFN-I (IFNα)**	-6.050	-8.728 to -3.373	Yes	***	0.0004
3	**HS** vs. **IFN-II (IFNγ)**	-10.09	-12.76 to -7.410	Yes	****	<0.0001
4	**HS** vs. **IFN-III (IFNλ)**	-0.1199	-2.797 to 2.558	No	ns	0.9239
5	**FBS** vs. **IL27**	-0.2012	-2.879 to 2.476	No	ns	0.8726

JAK2 TPM DATA							
Normality and Lognormality Tests		A	B	C	D	E	F
		HS	IFN-I (IFNα)	IFN-II (IFNγ)	IFN-III (IFNλ)	FBS	IL27
1	Test for normal distribution						
2	Shapiro-Wilk test						
3	W	0.7605	0.9244	0.8884	0.7601	0.9957	0.8719
4	P value	0.0234	0.4681	0.3494	0.0225	0.8750	0.3010
5	Passed normality test (alpha=0.05)?	No	Yes	Yes	No	Yes	Yes
6	P value summary	*	ns	ns	*	ns	ns

Ordinary one-way ANOVA<br>ANOVA results						
1	ANOVA summary					
2	F	332.4				
3	P value	<0.0001				
4	P value summary	****				
5	Significant diff. among means (P < 0.05)?	Yes				
6	R square	0.9928				
7						
8	Brown-Forsythe test					
9	F (DFn, DFd)	0.4914 (5, 12)				
10	P value	0.7767				
11	P value summary	ns				
12	Are SDs significantly different (P < 0.05)?	No				
13						
14	ANOVA table	SS	DF	MS	F (DFn, DFd)	P value
15	Treatment (between columns)	20317	5	4063	F (5, 12) = 332.4	P<0.0001
16	Residual (within columns)	146.7	12	12.23		
18	Total	20463	17			

Ordinary one-way ANOVA<br>Multiple comparisons						
1	Uncorrected Fisher's LSD	Mean Diff.	95.00% CI of diff.	Significant?	Summary	Individual P Value
2	**HS** vs. **IFN-I (IFNα)**	-43.03	-49.25 to -36.81	Yes	****	<0.0001
3	**HS** vs. **IFN-II (IFNγ)**	-94.67	-100.9 to -88.45	Yes	****	<0.0001
4	**HS** vs. **IFN-III (IFNλ)**	-7.056	-13.28 to -0.8362	Yes	*	0.0294
5	**FBS** vs. **IL27**	-15.02	-21.24 to -8.795	Yes	***	0.0002

JAK3 TPM DATA

Normality and Lognormality Tests		A	B	C	D	E	F
		HS	IFN-I (IFNα)	IFN-II (IFNγ)	IFN-III (IFNλ)	FBS	IL27
1	**Test for normal distribution**						
2	**Shapiro-Wilk test**						
3	W	0.9990	0.9619	0.9728	0.8638	0.8272	0.8193
4	P value	0.9407	0.6250	0.6836	0.2782	0.1812	0.1614
5	Passed normality test (alpha=0.05)?	Yes	Yes	Yes	Yes	Yes	Yes
6	P value summary	ns	ns	ns	ns	ns	ns

**Ordinary one-way ANOVA** ANOVA results						
1	**ANOVA summary**					
2	F	405.8				
3	P value	<0.0001				
4	P value summary	****				
5	Significant diff. among means (P < 0.05)?	Yes				
6	R square	0.9941				
7						
8	**Brown-Forsythe test**					
9	F (DFn, DFd)	0.6986 (5, 12)				
10	P value	0.6349				
11	P value summary	ns				
12	Are SDs significantly different (P < 0.05)?	No				
13						
14	**ANOVA table**	**SS**	**DF**	**MS**	**F (DFn, DFd)**	**P value**
15	Treatment (between columns)	524.9	5	105.0	F (5, 12) = 405.8	P<0.0001
16	Residual (within columns)	3.105	12	0.2587		
18	Total	528.0	17			

**Ordinary one-way ANOVA** Multiple comparisons						
1	**Uncorrected Fisher's LSD**	**Mean Diff.**	**95.00% CI of diff.**	**Significant?**	**Summary**	**Individual P Value**
2	**HS** vs. **IFN-I (IFNα)**	-2.399	-3.304 to -1.494	Yes	****	<0.0001
3	**HS** vs. **IFN-II (IFNγ)**	-11.32	-12.23 to -10.42	Yes	****	<0.0001
4	**HS** vs. **IFN-III (IFNλ)**	-0.5694	-1.474 to 0.3355	No	ns	0.1955
5	**FBS** vs. **IL27**	-10.83	-11.73 to -9.923	Yes	****	<0.0001

STAT1 TPM DATA

Normality and Lognormality Tests		A	B	C	D	E	F
		**HS**	**IFN-I (IFNα)**	**IFN-II (IFNy)**	**IFN-III (IFNλ)**	**FBS**	**IL27**
1	**Test for normal distribution**						
2	**Shapiro-Wilk test**						
3	W	0.9999	0.9774	0.9926	0.8010	0.8395	0.8454
4	P value	0.9808	0.7115	0.8358	0.1170	0.2128	0.2282
5	Passed normality test (alpha=0.05)?	Yes	Yes	Yes	Yes	Yes	Yes
6	P value summary	ns	ns	ns	ns	ns	ns

Ordinary one-way ANOVA<br>ANOVA results						
1	**ANOVA summary**					
2	F	1806				
3	P value	<0.0001				
4	P value summary	****				
5	Significant diff. among means (P < 0.05)?	Yes				
6	R square	0.9987				
7						
8	**Brown-Forsythe test**					
9	F (DFn, DFd)	1.308 (5, 12)				
10	P value	0.3240				
11	P value summary	ns				
12	Are SDs significantly different (P < 0.05)?	No				
13						
14	**ANOVA table**	**SS**	**DF**	**MS**	**F (DFn, DFd)**	**P value**
15	Treatment (between columns)	6274625	5	1254925	F (5, 12) = 1806	P<0.0001
16	Residual (within columns)	8339	12	694.9		
18	Total	6282964	17			

Ordinary one-way ANOVA<br>Multiple comparisons						
1	**Uncorrected Fisher's LSD**	**Mean Diff.**	**95.00% CI of diff.**	**Significant?**	**Summary**	**Individual P Value**
2	**HS** vs. **IFN-I (IFNα)**	-1237	-1284 to -1190	Yes	****	<0.0001
3	**HS** vs. **IFN-II (IFNy)**	-1532	-1579 to -1485	Yes	****	<0.0001
4	**HS** vs. **IFN-III (IFNλ)**	-785.3	-832.2 to -738.4	Yes	****	<0.0001
5	**FBS** vs. **IL27**	-975.3	-1022 to -928.4	Yes	****	<0.0001

STAT2 TPM DATA

Normality and Lognormality Tests		A	B	C	D	E	F
		HS	IFN-I (IFNα)	IFN-II (IFNy)	IFN-III (IFNλ)	FBS	IL27
1	**Test for normal distribution**						
2	**Shapiro-Wilk test**						
3	W	0.9984	0.9289	0.8825	0.7632	0.9136	0.9975
4	P value	0.9245	0.4845	0.3319	0.0293	0.4303	0.9041
5	Passed normality test (alpha=0.05)?	Yes	Yes	Yes	No	Yes	Yes
6	P value summary	ns	ns	ns	*	ns	ns

**Ordinary one-way ANOVA** ANOVA results						
1	**ANOVA summary**					
2	F	115.9				
3	P value	<0.0001				
4	P value summary	****				
5	Significant diff. among means (P < 0.05)?	Yes				
6	R square	0.9797				
7						
8	**Brown-Forsythe test**					
9	F (DFn, DFd)	1.057 (5, 12)				
10	P value	0.4301				
11	P value summary	ns				
12	Are SDs significantly different (P < 0.05)?	No				
13						
14	**ANOVA table**	**SS**	**DF**	**MS**	**F (DFn, DFd)**	**P value**
15	Treatment (between columns)	206850	5	41370	F (5, 12) = 115.9	P<0.0001
16	Residual (within columns)	4284	12	357.0		
18	Total	211134	17			

**Ordinary one-way ANOVA** Multiple comparisons						
1	**Uncorrected Fisher's LSD**	**Mean Diff.**	**95.00% CI of diff.**	**Significant?**	**Summary**	**Individual P Value**
2	**HS** vs. **IFN-I (IFNα)**	-301.6	-335.3 to -268.0	Yes	****	<0.0001
3	**HS** vs. **IFN-II (IFNy)**	-252.4	-286.0 to -218.8	Yes	****	<0.0001
4	**HS** vs. **IFN-III (IFNλ)**	-130.8	-164.4 to -97.16	Yes	****	<0.0001
5	**FBS** vs. **IL27**	-132.0	-165.6 to -98.35	Yes	****	<0.0001

STAT2 TPM DATA

	Normality and Lognormality Tests	A	B	C	D	E	F
		**HS**	**IFN-I (IFNα)**	**IFN-II (IFNγ)**	**IFN-III (IFNλ)**	**FBS**	**IL27**
1	**Test for normal distribution**						
2	**Shapiro-Wilk test**						
3	W	0.9993	0.9115	0.8296	0.8120	0.9983	0.9671
4	P value	0.9485	0.4230	0.1873	0.1434	0.9201	0.6519
5	Passed normality test (alpha=0.05)?	Yes	Yes	Yes	Yes	Yes	Yes
6	P value summary	ns	ns	ns	ns	ns	ns

	Ordinary one-way ANOVA<br>ANOVA results					
1	**ANOVA summary**					
2	F	363.2				
3	P value	<0.0001				
4	P value summary	****				
5	Significant diff. among means (P < 0.05)?	Yes				
6	R square	0.9934				
7						
8	**Brown-Forsythe test**					
9	F (DFn, DFd)	1.213 (5, 12)				
10	P value	0.3606				
11	P value summary	ns				
12	Are SDs significantly different (P < 0.05)?	No				
13						
14	**ANOVA table**	**SS**	**DF**	**MS**	**F (DFn, DFd)**	**P value**
15	Treatment (between columns)	22929	5	4586	F (5, 12) = 363.2	P<0.0001
16	Residual (within columns)	151.5	12	12.63		
18	Total	23080	17			

	Ordinary one-way ANOVA<br>Multiple comparisons					
1	**Uncorrected Fisher's LSD**	**Mean Diff.**	**95.00% CI of diff.**	**Significant?**	**Summary**	**Individual P Value**
2	**HS** vs. **IFN-I (IFNα)**	-44.54	-50.86 to -38.22	Yes	****	<0.0001
3	**HS** vs. **IFN-II (IFNγ)**	-99.19	-105.5 to -92.86	Yes	****	<0.0001
4	**HS** vs. **IFN-III (IFNλ)**	-16.38	-22.70 to -10.06	Yes	***	0.0001
5	**FBS** vs. **IL27**	-34.81	-41.14 to -28.49	Yes	****	<0.0001

SOCS1 TPM DATA

Normality and Lognormality Tests		A	B	C	D	E	F
		HS	IFN-I (IFNα)	IFN-II (IFNγ)	IFN-III (IFNλ)	FBS	IL27
1	**Test for normal distribution**						
2	**Shapiro-Wilk test**						
3	W	0.9984	0.7522	0.8861	0.9240	0.9512	0.9918
4	P value	0.9226	0.0048	0.3426	0.4664	0.5747	0.8267
5	Passed normality test (alpha=0.05)?	Yes	No	Yes	Yes	Yes	Yes
6	P value summary	ns	**	ns	ns	ns	ns

Ordinary one-way ANOVA<br>ANOVA results						
1	**ANOVA summary**					
2	F	785.0				
3	P value	<0.0001				
4	P value summary	****				
5	Significant diff. among means (P < 0.05)?	Yes				
6	R square	0.9970				
7						
8	**Brown-Forsythe test**					
9	F (DFn, DFd)	1.054 (5, 12)				
10	P value	0.4312				
11	P value summary	ns				
12	Are SDs significantly different (P < 0.05)?	No				
13						
14	**ANOVA table**	**SS**	**DF**	**MS**	**F (DFn, DFd)**	**P value**
15	Treatment (between columns)	20366	5	4073	F (5, 12) = 785.0	P<0.0001
16	Residual (within columns)	62.27	12	5.189		
18	Total	20428	17			

Ordinary one-way ANOVA<br>Multiple comparisons						
1	**Uncorrected Fisher's LSD**	**Mean Diff.**	**95.00% CI of diff.**	**Significant?**	**Summary**	**Individual P Value**
2	**HS** vs. **IFN-I (IFNα)**	-11.85	-15.90 to -7.799	Yes	****	<0.0001
3	**HS** vs. **IFN-II (IFNγ)**	-93.10	-97.15 to -89.05	Yes	****	<0.0001
4	**HS** vs. **IFN-III (IFNλ)**	-1.800	-5.853 to 2.252	No	ns	0.3522
5	**FBS** vs. **IL27**	-4.911	-8.963 to -0.8581	Yes	*	0.0216

SOCS3 TPM DATA

Normality and Lognormality Tests		A	B	C	D	E	F
		HS	IFN-I (IFNα)	IFN-II (IFNγ)	IFN-III (IFNλ)	FBS	IL27
1	**Test for normal distribution**						
2	**Shapiro-Wilk test**						
3	W	0.8321	0.7857	0.9848	0.9915	0.8236	0.8507
4	P value	0.1938	0.0809	0.7639	0.8235	0.1722	0.2422
5	Passed normality test (alpha=0.05)?	Yes	Yes	Yes	Yes	Yes	Yes
6	P value summary	ns	ns	ns	ns	ns	ns

Ordinary one-way ANOVA<br>ANOVA results						
1	**ANOVA summary**					
2	F	343.8				
3	P value	<0.0001				
4	P value summary	****				
5	Significant diff. among means (P < 0.05)?	Yes				
6	R square	0.9931				
7						
8	**Brown-Forsythe test**					
9	F (DFn, DFd)	1.684 (5, 12)				
10	P value	0.2128				
11	P value summary	ns				
12	Are SDs significantly different (P < 0.05)?	No				
13						
14	**ANOVA table**	**SS**	**DF**	**MS**	**F (DFn, DFd)**	**P value**
15	Treatment (between columns)	7679	5	1536	F (5, 12) = 343.8	P<0.0001
16	Residual (within columns)	53.61	12	4.468		
18	Total	7732	17			

Ordinary one-way ANOVA<br>Multiple comparisons						
1	**Uncorrected Fisher's LSD**	**Mean Diff.**	**95.00% CI of diff.**	**Significant?**	**Summary**	**Individual P Value**
2	**HS** vs. **IFN-I (IFNα)**	1.156	-2.604 to 4.916	No	ns	0.5156
3	**HS** vs. **IFN-II (IFNγ)**	-56.51	-60.27 to -52.75	Yes	****	<0.0001
4	**HS** vs. **IFN-III (IFNλ)**	-0.6223	-4.383 to 3.138	No	ns	0.7247
5	**FBS** vs. **IL27**	-12.51	-16.27 to -8.750	Yes	****	<0.0001

USP18 TPM DATA

Normality and Lognormality Tests		A	B	C	D	E	F
		HS	IFN-I (IFNα)	IFN-II (IFNγ)	IFN-III (IFNλ)	FBS	IL27
1	**Test for normal distribution**						
2	**Shapiro-Wilk test**						
3	W	0.9111	0.9948	0.8044	0.8411	0.9820	0.7548
4	P value	0.4216	0.8627	0.1250	0.2170	0.7432	0.0107
5	Passed normality test (alpha=0.05)?	Yes	Yes	Yes	Yes	Yes	No
6	P value summary	ns	ns	ns	ns	ns	*

Ordinary one-way ANOVA<br>ANOVA results						
1	**ANOVA summary**					
2	F	774.8				
3	P value	<0.0001				
4	P value summary	****				
5	Significant diff. among means (P < 0.05)?	Yes				
6	R square	0.9969				
7						
8	**Brown-Forsythe test**					
9	F (DFn, DFd)	2.902 (5, 12)				
10	P value	0.0606				
11	P value summary	ns				
12	Are SDs significantly different (P < 0.05)?	No				
13						
14	**ANOVA table**	**SS**	**DF**	**MS**	**F (DFn, DFd)**	**P value**
15	Treatment (between columns)	45596	5	9119	F (5, 12) = 774.8	P<0.0001
16	Residual (within columns)	141.2	12	11.77		
18	Total	45738	17			

Ordinary one-way ANOVA<br>Multiple comparisons						
1	**Uncorrected Fisher's LSD**	**Mean Diff.**	**95.00% CI of diff.**	**Significant?**	**Summary**	**Individual P Value**
2	**HS** vs. **IFN-I (IFNα)**	-134.1	-140.2 to -128.0	Yes	****	<0.0001
3	**HS** vs. **IFN-II (IFNγ)**	-6.078	-12.18 to -0.02481	Yes	*	0.0500
4	**HS** vs. **IFN-III (IFNλ)**	-68.31	-74.41 to -62.21	Yes	****	<0.0001
5	**FBS** vs. **IL27**	-3.056	-9.160 to 3.047	No	ns	0.2967

APOBEC3A TPM DATA							
Normality and Lognormality Tests		A	B	C	D	E	F
		HS	IFN-I (IFNα)	IFN-II (IFNγ)	IFN-III (IFNλ)	FBS	IL27
1	**Test for normal distribution**						
2	**Shapiro-Wilk test**						
3	W	0.9149	0.9591	0.8361	0.7659	0.7843	0.8563
4	P value	0.4346	0.6109	0.2040	0.0354	0.0775	0.2575
5	Passed normality test (alpha=0.05)?	Yes	Yes	Yes	No	Yes	Yes
6	P value summary	ns	ns	ns	*	ns	ns

Ordinary one-way ANOVA<br>ANOVA results						
1	**ANOVA summary**					
2	F	1718				
3	P value	<0.0001				
4	P value summary	****				
5	Significant diff. among means (P < 0.05)?	Yes				
6	R square	0.9986				
7						
8	**Brown-Forsythe test**					
9	F (DFn, DFd)	2.491 (5, 12)				
10	P value	0.0907				
11	P value summary	ns				
12	Are SDs significantly different (P < 0.05)?	No				
13						
14	**ANOVA table**	**SS**	**DF**	**MS**	**F (DFn, DFd)**	**P value**
15	Treatment (between columns)	2709990	5	541998	F (5, 12) = 1718	P<0.0001
16	Residual (within columns)	3786	12	315.5		
18	Total	2713776	17			

Ordinary one-way ANOVA<br>Multiple comparisons						
1	**Uncorrected Fisher's LSD**	**Mean Diff.**	**95.00% CI of diff.**	**Significant?**	**Summary**	**Individual P Value**
2	**HS** vs. **IFN-I (IFNα)**	-1059	-1090 to -1027	Yes	****	<0.0001
3	**HS** vs. **IFN-II (IFNγ)**	-18.81	-50.41 to 12.79	No	ns	0.2191
4	**HS** vs. **IFN-III (IFNλ)**	-67.82	-99.43 to -36.22	Yes	***	0.0005
5	**FBS** vs. **IL27**	-11.76	-43.36 to -19.84	Yes	*	0.0333

GBP5 TPM DATA							
Normality and Lognormality Tests		A	B	C	D	E	F
		HS	IFN-I (IFNα)	IFN-II (IFNγ)	IFN-III (IFNλ)	FBS	IL27
1	**Test for normal distribution**						
2	**Shapiro-Wilk test**						
3	W	0.8757	0.7962	0.9395	0.8154	0.9439	0.9984
4	P value	0.3118	0.1054	0.5254	0.1518	0.5431	0.9235
5	Passed normality test (alpha=0.05)?	Yes	Yes	Yes	Yes	Yes	Yes
6	P value summary	ns	ns	ns	ns	ns	ns

Ordinary one-way ANOVA<br>ANOVA results						
1	**ANOVA summary**					
2	F	1582				
3	P value	<0.0001				
4	P value summary	****				
5	Significant diff. among means (P < 0.05)?	Yes				
6	R square	0.9985				
7						
8	**Brown-Forsythe test**					
9	F (DFn, DFd)	2.520 (5, 12)				
10	P value	0.0881				
11	P value summary	ns				
12	Are SDs significantly different (P < 0.05)?	No				
13						
14	**ANOVA table**	**SS**	**DF**	**MS**	**F (DFn, DFd)**	**P value**
15	Treatment (between columns)	3771782	5	754356	F (5, 12) = 1582	P<0.0001
16	Residual (within columns)	5724	12	477.0		
18	Total	3777506	17			

Ordinary one-way ANOVA<br>Multiple comparisons						
1	**Uncorrected Fisher's LSD**	**Mean Diff.**	**95.00% CI of diff.**	**Significant?**	**Summary**	**Individual P Value**
2	**HS** vs. **IFN-I (IFNα)**	-124.6	-163.5 to -85.78	Yes	****	<0.0001
3	**HS** vs. **IFN-II (IFNγ)**	-1289	-1328 to -1250	Yes	****	<0.0001
4	**HS** vs. **IFN-III (IFNλ)**	-33.28	-72.14 to 5.569	No	ns	0.0866
5	**FBS** vs. **IL27**	-315.6	-354.5 to -276.8	Yes	****	<0.0001

IDO1 TPM DATA

Normality and Lognormality Tests		A	B	C	D	E	F
		HS	IFN-I (IFNα)	IFN-II (IFNγ)	IFN-III (IFNλ)	FBS	IL27
1	**Test for normal distribution**						
2	**Shapiro-Wilk test**						
3	W	0.7855	0.8886	0.8642	0.9340	0.7821	0.9454
4	P value	0.0803	0.3502	0.2793	0.5036	0.0723	0.5495
5	Passed normality test (alpha=0.05)?	Yes	Yes	Yes	Yes	Yes	Yes
6	P value summary	ns	ns	ns	ns	ns	ns

**Ordinary one-way ANOVA** ANOVA results						
1	**ANOVA summary**					
2	F	8223				
3	P value	<0.0001				
4	P value summary	****				
5	Significant diff. among means (P < 0.05)?	Yes				
6	R square	0.9997				
7						
8	**Brown-Forsythe test**					
9	F (DFn, DFd)	1.337 (5, 12)				
10	P value	0.3138				
11	P value summary	ns				
12	Are SDs significantly different (P < 0.05)?	No				
13						
14	**ANOVA table**	**SS**	**DF**	**MS**	**F (DFn, DFd)**	**P value**
15	Treatment (between columns)	24673823	5	4934765	F (5, 12) = 8223	P<0.0001
16	Residual (within columns)	7202	12	600.1		
18	Total	24681025	17			

**Ordinary one-way ANOVA** Multiple comparisons						
1	**Uncorrected Fisher's LSD**	**Mean Diff.**	**95.00% CI of diff.**	**Significant?**	**Summary**	**Individual P Value**
2	**HS** vs. **IFN-I (IFNα)**	-394.8	-438.4 to -351.3	Yes	****	<0.0001
3	**HS** vs. **IFN-II (IFNγ)**	-3215	-3258 to -3171	Yes	****	<0.0001
4	**HS** vs. **IFN-III (IFNλ)**	-54.11	-97.69 to -10.53	Yes	*	0.0191
5	**FBS** vs. **IL27**	-25.60	-69.18 to -17.98	Yes	*	0.0248

ISG15 TPM DATA							
Normality and Lognormality Tests		A	B	C	D	E	F
		HS	IFN-I (IFNα)	IFN-II (IFNy)	IFN-III (IFNλ)	FBS	IL27
1	**Test for normal distribution**						
2	**Shapiro-Wilk test**						
3	W	0.9692	0.9901	0.8376	0.9879	0.9495	0.9898
4	P value	0.6631	0.8098	0.2078	0.7894	0.5670	0.8071
5	Passed normality test (alpha=0.05)?	Yes	Yes	Yes	Yes	Yes	Yes
6	P value summary	ns	ns	ns	ns	ns	ns

**Ordinary one-way ANOVA**<br>ANOVA results						
1	**ANOVA summary**					
2	F	2284				
3	P value	<0.0001				
4	P value summary	****				
5	Significant diff. among means (P < 0.05)?	Yes				
6	R square	0.9990				
7						
8	**Brown-Forsythe test**					
9	F (DFn, DFd)	2.650 (5, 12)				
10	P value	0.0774				
11	P value summary	ns				
12	Are SDs significantly different (P < 0.05)?	No				
13						
14	**ANOVA table**	**SS**	**DF**	**MS**	**F (DFn, DFd)**	**P value**
15	Treatment (between columns)	119424809	5	23884962	F (5, 12) = 2284	P<0.0001
16	Residual (within columns)	125481	12	10457		
18	Total	119550290	17			

**Ordinary one-way ANOVA**<br>Multiple comparisons						
1	**Uncorrected Fisher's LSD**	**Mean Diff.**	**95.00% CI of diff.**	**Significant?**	**Summary**	**Individual P Value**
2	**HS** vs. **IFN-I (IFNα)**	-7009	-7190 to -6827	Yes	****	<0.0001
3	**HS** vs. **IFN-II (IFNy)**	-168.6	-350.6 to 13.27	No	ns	0.0663
4	**HS** vs. **IFN-III (IFNλ)**	-2594	-2776 to -2412	Yes	****	<0.0001
5	**FBS** vs. **IL27**	-43.60	-225.5 to -138.3	Yes	*	0.0410

MX1 TPM DATA

Normality and Lognormality Tests		A<br>HS	B<br>IFN-I (IFNα)	C<br>IFN-II (IFNγ)	D<br>IFN-III (IFNλ)	E<br>FBS	F<br>IL27
1	**Test for normal distribution**						
2	**Shapiro-Wilk test**						
3	W	0.9149	0.7777	0.9734	0.9329	0.9718	0.9485
4	P value	0.4345	0.0623	0.6874	0.4996	0.6780	0.5628
5	Passed normality test (alpha=0.05)?	Yes	Yes	Yes	Yes	Yes	Yes
6	P value summary	ns	ns	ns	ns	ns	ns

**Ordinary one-way ANOVA**<br>ANOVA results						
1	**ANOVA summary**					
2	F	874.9				
3	P value	<0.0001				
4	P value summary	****				
5	Significant diff. among means (P < 0.05)?	Yes				
6	R square	0.9973				
7						
8	**Brown-Forsythe test**					
9	F (DFn, DFd)	0.7574 (5, 12)				
10	P value	0.5970				
11	P value summary	ns				
12	Are SDs significantly different (P < 0.05)?	No				
13						
14	**ANOVA table**	**SS**	**DF**	**MS**	**F (DFn, DFd)**	**P value**
15	Treatment (between columns)	4757026	5	951405	F (5, 12) = 874.9	P<0.0001
16	Residual (within columns)	13049	12	1087		
18	Total	4770076	17			

**Ordinary one-way ANOVA**<br>Multiple comparisons						
1	**Uncorrected Fisher's LSD**	**Mean Diff.**	**95.00% CI of diff.**	**Significant?**	**Summary**	**Individual P Value**
2	**HS** vs. **IFN-I (IFNα)**	-1412	-1470 to -1353	Yes	****	<0.0001
3	**HS** vs. **IFN-II (IFNγ)**	-288.7	-347.3 to -230.0	Yes	****	<0.0001
4	**HS** vs. **IFN-III (IFNλ)**	-841.4	-900.1 to -782.7	Yes	****	<0.0001
5	**FBS** vs. **IL27**	-132.0	-190.7 to -73.32	Yes	***	0.0004

OAS2 TPM DATA							
Normality and Lognormality Tests		A	B	C	D	E	F
		HS	IFN-I (IFNα)	IFN-II (IFNγ)	IFN-III (IFNλ)	FBS	IL27
1	**Test for normal distribution**						
2	**Shapiro-Wilk test**						
3	W	0.9946	0.8348	0.8654	0.9459	0.8590	0.8501
4	P value	0.8596	0.2005	0.2827	0.5516	0.2648	0.2408
5	Passed normality test (alpha=0.05)?	Yes	Yes	Yes	Yes	Yes	Yes
6	P value summary	ns	ns	ns	ns	ns	ns

**Ordinary one-way ANOVA** ANOVA results						
1	**ANOVA summary**					
2	F	6434				
3	P value	<0.0001				
4	P value summary	****				
5	Significant diff. among means (P < 0.05)?	Yes				
6	R square	0.9996				
7						
8	**Brown-Forsythe test**					
9	F (DFn, DFd)	0.8353 (5, 12)				
10	P value	0.5493				
11	P value summary	ns				
12	Are SDs significantly different (P < 0.05)?	No				
13						
14	**ANOVA table**	**SS**	**DF**	**MS**	**F (DFn, DFd)**	**P value**
15	Treatment (between columns)	495373	5	99075	F (5, 12) = 6434	P<0.0001
16	Residual (within columns)	184.8	12	15.40		
18	Total	495557	17			

**Ordinary one-way ANOVA** Multiple comparisons						
1	**Uncorrected Fisher's LSD**	**Mean Diff.**	**95.00% CI of diff.**	**Significant?**	**Summary**	**Individual P Value**
2	**HS** vs. **IFN-I (IFNα)**	-451.2	-458.1 to -444.2	Yes	****	<0.0001
3	**HS** vs. **IFN-II (IFNγ)**	-65.89	-72.87 to -58.91	Yes	****	<0.0001
4	**HS** vs. **IFN-III (IFNλ)**	-241.9	-248.8 to -234.9	Yes	****	<0.0001
5	**FBS** vs. **IL27**	-20.45	-27.43 to -13.47	Yes	****	<0.0001

Tetherin TPM DATA

Normality and Lognormality Tests		A	B	C	D	E	F
		HS	IFN-I (IFNα)	IFN-II (IFNγ)	IFN-III (IFNλ)	FBS	IL27
1	**Test for normal distribution**						
2	**Shapiro-Wilk test**						
3	W	0.9654	0.9960	0.9712	0.8988	0.9133	0.9975
4	P value	0.6425	0.8795	0.6741	0.3816	0.4293	0.9041
5	Passed normality test (alpha=0.05)?	Yes	Yes	Yes	Yes	Yes	Yes
6	P value summary	ns	ns	ns	ns	ns	ns

**Ordinary one-way ANOVA** ANOVA results						
1	**ANOVA summary**					
2	F	740.4				
3	P value	<0.0001				
4	P value summary	****				
5	Significant diff. among means (P < 0.05)?	Yes				
6	R square	0.9968				
7						
8	**Brown-Forsythe test**					
9	F (DFn, DFd)	0.7270 (5, 12)				
10	P value	0.6164				
11	P value summary	ns				
12	Are SDs significantly different (P < 0.05)?	No				
13						
14	**ANOVA table**	**SS**	**DF**	**MS**	**F (DFn, DFd)**	**P value**
15	Treatment (between columns)	4530874	5	906175	F (5, 12) = 740.4	P<0.0001
16	Residual (within columns)	14686	12	1224		
18	Total	4545561	17			

**Ordinary one-way ANOVA** Multiple comparisons						
1	**Uncorrected Fisher's LSD**	**Mean Diff.**	**95.00% CI of diff.**	**Significant?**	**Summary**	**Individual P Value**
2	**HS** vs. **IFN-I (IFNα)**	-1313	-1376 to -1251	Yes	****	<0.0001
3	**HS** vs. **IFN-II (IFNγ)**	-330.7	-393.0 to -268.5	Yes	****	<0.0001
4	**HS** vs. **IFN-III (IFNλ)**	-798.1	-860.3 to -735.9	Yes	****	<0.0001
5	**FBS** vs. **IL27**	-122.7	-185.0 to -60.49	Yes	**	0.0010

Viperin TPM DATA

Normality and Lognormality Tests		A	B	C	D	E	F
		HS	IFN-I (IFNα)	IFN-II (IFNγ)	IFN-III (IFNλ)	FBS	IL27
1	**Test for normal distribution**						
2	**Shapiro-Wilk test**						
3	W	0.7703	0.9861	0.8879	0.8799	0.8829	0.9849
4	P value	0.0455	0.7741	0.3479	0.3240	0.3330	0.7647
5	Passed normality test (alpha=0.05)?	No	Yes	Yes	Yes	Yes	Yes
6	P value summary	*	ns	ns	ns	ns	ns

**Ordinary one-way ANOVA** ANOVA results						
1	**ANOVA summary**					
2	F	1910				
3	P value	<0.0001				
4	P value summary	****				
5	Significant diff. among means (P < 0.05)?	Yes				
6	R square	0.9987				
7						
8	**Brown-Forsythe test**					
9	F (DFn, DFd)	3.141 (5, 12)				
10	P value	0.0484				
11	P value summary	*				
12	Are SDs significantly different (P < 0.05)?	Yes				
13						
14	**ANOVA table**	**SS**	**DF**	**MS**	**F (DFn, DFd)**	**P value**
15	Treatment (between columns)	25401587	5	5080317	F (5, 12) = 1910	P<0.0001
16	Residual (within columns)	31925	12	2660		
18	Total	25433512	17			

**Ordinary one-way ANOVA** Multiple comparisons						
1	**Uncorrected Fisher's LSD**	**Mean Diff.**	**95.00% CI of diff.**	**Significant?**	**Summary**	**Individual P Value**
2	**HS** vs. **IFN-I (IFNα)**	-3296	-3388 to -3204	Yes	****	<0.0001
3	**HS** vs. **IFN-II (IFNγ)**	-487.8	-579.5 to -396.0	Yes	****	<0.0001
4	**HS** vs. **IFN-III (IFNλ)**	-1319	-1411 to -1227	Yes	****	<0.0001
5	**FBS** vs. **IL27**	-29.27	-121.0 to -62.48	Yes	*	0.0490

Tlr2 TPM DATA							
Normality and Lognormality Tests		A	B	C	D	E	F
		HS	IFN-I (IFNα)	IFN-II (IFNy)	IFN-III (IFNλ)	FBS	IL27
1	Test for normal distribution						
2	Shapiro-Wilk test						
3	W	0.9341	0.8465	0.9697	0.9814	0.9987	0.8371
4	P value	0.5043	0.2311	0.6661	0.7386	0.9309	0.2066
5	Passed normality test (alpha=0.05)?	Yes	Yes	Yes	Yes	Yes	Yes
6	P value summary	ns	ns	ns	ns	ns	ns

Ordinary one-way ANOVA ANOVA results						
1	ANOVA summary					
2	F	132.8				
3	P value	<0.0001				
4	P value summary	****				
5	Significant diff. among means (P < 0.05)?	Yes				
6	R square	0.9822				
7						
8	Brown-Forsythe test					
9	F (DFn, DFd)	0.5825 (5, 12)				
10	P value	0.7133				
11	P value summary	ns				
12	Are SDs significantly different (P < 0.05)?	No				
13						
14	ANOVA table	SS	DF	MS	F (DFn, DFd)	P value
15	Treatment (between columns)	13856	5	2771	F (5, 12) = 132.8	P<0.0001
16	Residual (within columns)	250.4	12	20.87		
18	Total	14107	17			

Ordinary one-way ANOVA Multiple comparisons						
1	Uncorrected Fisher's LSD	Mean Diff.	95.00% CI of diff.	Significant?	Summary	Individual P Value
2	**HS** vs. **IFN-I (IFNα)**	-31.39	-39.52 to -23.27	Yes	****	<0.0001
3	**HS** vs. **IFN-II (IFNy)**	-41.57	-49.69 to -33.44	Yes	****	<0.0001
4	**HS** vs. **IFN-III (IFNλ)**	-3.157	-11.28 to 4.970	No	ns	0.4140
5	**FBS** vs. **IL27**	-9.316	-17.44 to -1.189	Yes	*	0.0280

TLR2 TPM DATA							
Normality and Lognormality Tests		A	B	C	D	E	F
		HS	IFN-I (IFNα)	IFN-II (IFNγ)	IFN-III (IFNλ)	FBS	IL27
1	Test for normal distribution						
2	Shapiro-Wilk test						
3	W	0.9944	0.7568	0.8272	0.7796	0.9230	0.9623
4	P value	0.8570	0.0151	0.1813	0.0667	0.4631	0.6270
5	Passed normality test (alpha=0.05)?	Yes	No	Yes	Yes	Yes	Yes
6	P value summary	ns	*	ns	ns	ns	ns

Ordinary one-way ANOVA<br>ANOVA results						
1	ANOVA summary					
2	F	13.58				
3	P value	0.0001				
4	P value summary	***				
5	Significant diff. among means (P < 0.05)?	Yes				
6	R square	0.8498				
7						
8	Brown-Forsythe test					
9	F (DFn, DFd)	0.8643 (5, 12)				
10	P value	0.5322				
11	P value summary	ns				
12	Are SDs significantly different (P < 0.05)?	No				
13						
14	ANOVA table	SS	DF	MS	F (DFn, DFd)	P value
15	Treatment (between columns)	1476	5	295.2	F (5, 12) = 13.58	P=0.0001
16	Residual (within columns)	260.9	12	21.74		
18	Total	1737	17			

Ordinary one-way ANOVA<br>Multiple comparisons						
1	Uncorrected Fisher's LSD	Mean Diff.	95.00% CI of diff.	Significant?	Summary	Individual P Value
2	**HS** vs. **IFN-I (IFNα)**	-24.26	-32.55 to -15.96	Yes	****	<0.0001
3	**HS** vs. **IFN-II (IFNγ)**	-10.08	-18.38 to -1.788	Yes	*	0.0212
4	**HS** vs. **IFN-III (IFNλ)**	-3.022	-11.32 to 5.273	No	ns	0.4427
5	**FBS** vs. **IL27**	-0.03846	-8.333 to 8.257	No	ns	0.9921

TLR3 TPM DATA

Normality and Lognormality Tests		A	B	C	D	E	F
		HS	IFN-I (IFNα)	IFN-II (IFNγ)	IFN-III (IFNλ)	FBS	IL27
1	Test for normal distribution						
2	Shapiro-Wilk test						
3	W	0.8256	0.9617	0.9945	0.9650	0.8110	0.8276
4	P value	0.1771	0.6236	0.8584	0.6407	0.1410	0.1822
5	Passed normality test (alpha=0.05)?	Yes	Yes	Yes	Yes	Yes	Yes
6	P value summary	ns	ns	ns	ns	ns	ns

Ordinary one-way ANOVA<br>ANOVA results						
1	ANOVA summary					
2	F	448.8				
3	P value	<0.0001				
4	P value summary	****				
5	Significant diff. among means (P < 0.05)?	Yes				
6	R square	0.9947				
7						
8	Brown-Forsythe test					
9	F (DFn, DFd)	1.502 (5, 12)				
10	P value	0.2605				
11	P value summary	ns				
12	Are SDs significantly different (P < 0.05)?	No				
13						
14	ANOVA table	SS	DF	MS	F (DFn, DFd)	P value
15	Treatment (between columns)	1872	5	374.3	F (5, 12) = 448.8	P<0.0001
16	Residual (within columns)	10.01	12	0.8339		
18	Total	1882	17			

Ordinary one-way ANOVA<br>Multiple comparisons						
1	Uncorrected Fisher's LSD	Mean Diff.	95.00% CI of diff.	Significant?	Summary	Individual P Value
2	**HS** vs. **IFN-I (IFNα)**	-28.44	-30.07 to -26.82	Yes	****	<0.0001
3	**HS** vs. **IFN-II (IFNγ)**	-4.961	-6.586 to -3.337	Yes	****	<0.0001
4	**HS** vs. **IFN-III (IFNλ)**	-7.480	-9.104 to -5.855	Yes	****	<0.0001
5	**FBS** vs. **IL27**	-1.247	-2.871 to -0.3777	Yes	*	0.0420

TLR4 TPM DATA

Normality and Lognormality Tests		A	B	C	D	E	F
		HS	IFN-I (IFNα)	IFN-II (IFNγ)	IFN-III (IFNλ)	FBS	IL27
1	**Test for normal distribution**						
2	**Shapiro-Wilk test**						
3	W	0.9816	0.9302	0.9576	0.8330	0.9335	0.9344
4	P value	0.7399	0.4893	0.6041	0.1960	0.5020	0.5054
5	Passed normality test (alpha=0.05)?	Yes	Yes	Yes	Yes	Yes	Yes
6	P value summary	ns	ns	ns	ns	ns	ns

**Ordinary one-way ANOVA** ANOVA results						
1	**ANOVA summary**					
2	F	138.0				
3	P value	<0.0001				
4	P value summary	****				
5	Significant diff. among means (P < 0.05)?	Yes				
6	R square	0.9829				
7						
8	**Brown-Forsythe test**					
9	F (DFn, DFd)	0.8806 (5, 12)				
10	P value	0.5228				
11	P value summary	ns				
12	Are SDs significantly different (P < 0.05)?	No				
13						
14	**ANOVA table**	**SS**	**DF**	**MS**	**F (DFn, DFd)**	**P value**
15	Treatment (between columns)	15199	5	3040	F (5, 12) = 138.0	P<0.0001
16	Residual (within columns)	264.3	12	22.02		
18	Total	15463	17			

**Ordinary one-way ANOVA** Multiple comparisons						
1	**Uncorrected Fisher's LSD**	**Mean Diff.**	**95.00% CI of diff.**	**Significant?**	**Summary**	**Individual P Value**
2	**HS** vs. **IFN-I (IFNα)**	-30.74	-39.09 to -22.39	Yes	****	<0.0001
3	**HS** vs. **IFN-II (IFNγ)**	-73.87	-82.22 to -65.53	Yes	****	<0.0001
4	**HS** vs. **IFN-III (IFNλ)**	2.264	-6.085 to 10.61	No	ns	0.5656
5	**FBS** vs. **IL27**	-1.242	-9.590 to 7.107	No	ns	0.7515

TLR7 TPM DATA

Normality and Lognormality Tests		A	B	C	D	E	F
		HS	IFN-I (IFNα)	IFN-II (IFNγ)	IFN-III (IFNλ)	FBS	IL27
1	Test for normal distribution						
2	Shapiro-Wilk test						
3	W	0.9785	0.7550	0.9895	0.9977	0.9773	0.8913
4	P value	0.7189	0.0111	0.8039	0.9077	0.7113	0.3583
5	Passed normality test (alpha=0.05)?	Yes	No	Yes	Yes	Yes	Yes
6	P value summary	ns	*	ns	ns	ns	ns

Ordinary one-way ANOVA<br>ANOVA results						
1	ANOVA summary					
2	F	156.1				
3	P value	<0.0001				
4	P value summary	****				
5	Significant diff. among means (P < 0.05)?	Yes				
6	R square	0.9849				
7						
8	Brown-Forsythe test					
9	F (DFn, DFd)	0.7507 (5, 12)				
10	P value	0.6013				
11	P value summary	ns				
12	Are SDs significantly different (P < 0.05)?	No				
13						
14	ANOVA table	SS	DF	MS	F (DFn, DFd)	P value
15	Treatment (between columns)	323.0	5	64.59	F (5, 12) = 156.1	P<0.0001
16	Residual (within columns)	4.965	12	0.4137		
18	Total	327.9	17			

Ordinary one-way ANOVA<br>Multiple comparisons						
1	Uncorrected Fisher's LSD	Mean Diff.	95.00% CI of diff.	Significant?	Summary	Individual P Value
2	**HS** vs. **IFN-I (IFNα)**	-8.810	-9.954 to -7.666	Yes	****	<0.0001
3	**HS** vs. **IFN-II (IFNγ)**	-7.299	-8.443 to -6.154	Yes	****	<0.0001
4	**HS** vs. **IFN-III (IFNλ)**	0.8729	-0.2714 to 2.017	No	ns	0.1224
5	**FBS** vs. **IL27**	-0.1142	-1.258 to 1.030	No	ns	0.8316

TLR8 TPM DATA

Normality and Lognormality Tests		A	B	C	D	E	F
		HS	IFN-I (IFNα)	IFN-II (IFNγ)	IFN-III (IFNλ)	FBS	IL27
1	Test for normal distribution						
2	Shapiro-Wilk test						
3	W	0.8949	0.8642	0.7534	0.8252	0.7768	0.9520
4	P value	0.3693	0.2792	0.0076	0.1761	0.0602	0.5781
5	Passed normality test (alpha=0.05)?	Yes	Yes	No	Yes	Yes	Yes
6	P value summary	ns	ns	**	ns	ns	ns

Ordinary one-way ANOVA<br>ANOVA results						
1	ANOVA summary					
2	F	273.8				
3	P value	<0.0001				
4	P value summary	****				
5	Significant diff. among means (P < 0.05)?	Yes				
6	R square	0.9913				
7						
8	Brown-Forsythe test					
9	F (DFn, DFd)	0.5005 (5, 12)				
10	P value	0.7704				
11	P value summary	ns				
12	Are SDs significantly different (P < 0.05)?	No				
13						
14	ANOVA table	SS	DF	MS	F (DFn, DFd)	P value
15	Treatment (between columns)	8066	5	1613	F (5, 12) = 273.8	P<0.0001
16	Residual (within columns)	70.71	12	5.892		
18	Total	8137	17			

Ordinary one-way ANOVA<br>Multiple comparisons						
1	Uncorrected Fisher's LSD	Mean Diff.	95.00% CI of diff.	Significant?	Summary	Individual P Value
2	**HS** vs. **IFN-I (IFNα)**	-15.18	-19.50 to -10.86	Yes	****	<0.0001
3	**HS** vs. **IFN-II (IFNγ)**	-60.78	-65.10 to -56.46	Yes	****	<0.0001
4	**HS** vs. **IFN-III (IFNλ)**	3.702	-0.6167 to 8.020	No	ns	0.0864
5	**FBS** vs. **IL27**	-8.970	-13.29 to -4.651	Yes	***	0.0007

MyD88 TPM DATA

	Normality and Lognormality Tests	A	B	C	D	E	F
		HS	IFN-I (IFNα)	IFN-II (IFNγ)	IFN-III (IFNλ)	FBS	IL27
1	**Test for normal distribution**						
2	**Shapiro-Wilk test**						
3	W	0.9071	0.9845	0.9863	0.9732	0.9363	0.9955
4	P value	0.4085	0.7619	0.7763	0.6861	0.5126	0.8716
5	Passed normality test (alpha=0.05)?	Yes	Yes	Yes	Yes	Yes	Yes
6	P value summary	ns	ns	ns	ns	ns	ns

	**Ordinary one-way ANOVA** ANOVA results					
1	**ANOVA summary**					
2	F	281.7				
3	P value	<0.0001				
4	P value summary	****				
5	Significant diff. among means (P < 0.05)?	Yes				
6	R square	0.9916				
7						
8	**Brown-Forsythe test**					
9	F (DFn, DFd)	1.861 (5, 12)				
10	P value	0.1752				
11	P value summary	ns				
12	Are SDs significantly different (P < 0.05)?	No				
13						
14	**ANOVA table**	**SS**	**DF**	**MS**	**F (DFn, DFd)**	**P value**
15	Treatment (between columns)	430994	5	86199	F (5, 12) = 281.7	P<0.0001
16	Residual (within columns)	3672	12	306.0		
18	Total	434666	17			

	**Ordinary one-way ANOVA** Multiple comparisons					
1	**Uncorrected Fisher's LSD**	**Mean Diff.**	**95.00% CI of diff.**	**Significant?**	**Summary**	**Individual P Value**
2	**HS** vs. **IFN-I (IFNα)**	-382.9	-414.0 to -351.8	Yes	****	<0.0001
3	**HS** vs. **IFN-II (IFNγ)**	-18.06	-49.18 to 13.06	No	ns	0.2300
4	**HS** vs. **IFN-III (IFNλ)**	-205.9	-237.0 to -174.8	Yes	****	<0.0001
5	**FBS** vs. **IL27**	-43.62	-74.74 to -12.50	Yes	*	0.0100

TRIF TPM DATA

Normality and Lognormality Tests		A	B	C	D	E	F
		HS	IFN-I (IFNα)	IFN-II (IFNγ)	IFN-III (IFNλ)	FBS	IL27
1	**Test for normal distribution**						
2	**Shapiro-Wilk test**						
3	W	0.8919	0.9002	0.8211	0.8463	0.9758	0.9871
4	P value	0.3603	0.3863	0.1660	0.2305	0.7017	0.7825
5	Passed normality test (alpha=0.05)?	Yes	Yes	Yes	Yes	Yes	Yes
6	P value summary	ns	ns	ns	ns	ns	ns

Ordinary one-way ANOVA<br>ANOVA results						
1	**ANOVA summary**					
2	F	80.61				
3	P value	<0.0001				
4	P value summary	****				
5	Significant diff. among means (P < 0.05)?	Yes				
6	R square	0.9711				
7						
8	**Brown-Forsythe test**					
9	F (DFn, DFd)	0.2611 (5, 12)				
10	P value	0.9258				
11	P value summary	ns				
12	Are SDs significantly different (P < 0.05)?	No				
13						
14	**ANOVA table**	**SS**	**DF**	**MS**	**F (DFn, DFd)**	**P value**
15	Treatment (between columns)	3153	5	630.7	F (5, 12) = 80.61	P<0.0001
16	Residual (within columns)	93.89	12	7.824		
18	Total	3247	17			

Ordinary one-way ANOVA<br>Multiple comparisons						
1	**Uncorrected Fisher's LSD**	**Mean Diff.**	**95.00% CI of diff.**	**Significant?**	**Summary**	**Individual P Value**
2	**HS** vs. **IFN-I (IFNα)**	-21.52	-26.49 to -16.54	Yes	****	<0.0001
3	**HS** vs. **IFN-II (IFNγ)**	-39.53	-44.51 to -34.55	Yes	****	<0.0001
4	**HS** vs. **IFN-III (IFNλ)**	-4.181	-9.157 to 0.7953	No	ns	0.0921
5	**FBS** vs. **IL27**	-8.156	-13.13 to -3.180	Yes	**	0.0038

AIM2 TPM DATA

Normality and Lognormality Tests		A	B	C	D	E	F
		HS	IFN-I (IFNα)	IFN-II (IFNγ)	IFN-III (IFNλ)	FBS	IL27
1	Test for normal distribution						
2	Shapiro-Wilk test						
3	W	0.7525	0.9488	0.9989	0.8596	0.9326	0.8701
4	P value	0.0055	0.5641	0.9358	0.2665	0.4985	0.2957
5	Passed normality test (alpha=0.05)?	No	Yes	Yes	Yes	Yes	Yes
6	P value summary	**	ns	ns	ns	ns	ns

Ordinary one-way ANOVA<br>ANOVA results						
1	ANOVA summary					
2	F	204.1				
3	P value	<0.0001				
4	P value summary	****				
5	Significant diff. among means (P < 0.05)?	Yes				
6	R square	0.9884				
7						
8	Brown-Forsythe test					
9	F (DFn, DFd)	1.790 (5, 12)				
10	P value	0.1893				
11	P value summary	ns				
12	Are SDs significantly different (P < 0.05)?	No				
13						
14	ANOVA table	SS	DF	MS	F (DFn, DFd)	P value
15	Treatment (between columns)	11427	5	2285	F (5, 12) = 204.1	P<0.0001
16	Residual (within columns)	134.4	12	11.20		
18	Total	11562	17			

Ordinary one-way ANOVA<br>Multiple comparisons						
1	Uncorrected Fisher's LSD	Mean Diff.	95.00% CI of diff.	Significant?	Summary	Individual P Value
2	**HS** vs. **IFN-I (IFNα)**	-61.38	-67.33 to -55.42	Yes	****	<0.0001
3	**HS** vs. **IFN-II (IFNγ)**	-53.48	-59.43 to -47.52	Yes	****	<0.0001
4	**HS** vs. **IFN-III (IFNλ)**	-13.35	-19.30 to -7.395	Yes	***	0.0004
5	**FBS** vs. **IL27**	-6.594	-12.55 to -0.6398	Yes	*	0.0327

cGAS TPM DATA

Normality and Lognormality Tests		A	B	C	D	E	F
		HS	IFN-I (IFNα)	IFN-II (IFNγ)	IFN-III (IFNλ)	FBS	IL27
1	**Test for normal distribution**						
2	**Shapiro-Wilk test**						
3	W	0.9907	0.7805	0.9957	0.9995	0.9005	0.7631
4	P value	0.8160	0.0687	0.8745	0.9585	0.3872	0.0291
5	Passed normality test (alpha=0.05)?	Yes	Yes	Yes	Yes	Yes	No
6	P value summary	ns	ns	ns	ns	ns	*

Ordinary one-way ANOVA
ANOVA results

1	**ANOVA summary**					
2	F	119.9				
3	P value	<0.0001				
4	P value summary	****				
5	Significant diff. among means (P < 0.05)?	Yes				
6	R square	0.9804				
7						
8	**Brown-Forsythe test**					
9	F (DFn, DFd)	0.1717 (5, 12)				
10	P value	0.9682				
11	P value summary	ns				
12	Are SDs significantly different (P < 0.05)?	No				
13						
14	**ANOVA table**	**SS**	**DF**	**MS**	**F (DFn, DFd)**	**P value**
15	Treatment (between columns)	1924	5	384.8	F (5, 12) = 119.9	P<0.0001
16	Residual (within columns)	38.51	12	3.209		
18	Total	1963	17			

Ordinary one-way ANOVA
Multiple comparisons

1	**Uncorrected Fisher's LSD**	**Mean Diff.**	**95.00% CI of diff.**	**Significant?**	**Summary**	**Individual P Value**
2	**HS** vs. **IFN-I (IFNα)**	-24.95	-28.14 to -21.77	Yes	****	<0.0001
3	**HS** vs. **IFN-II (IFNγ)**	-24.12	-27.30 to -20.93	Yes	****	<0.0001
4	**HS** vs. **IFN-III (IFNλ)**	-6.586	-9.773 to -3.399	Yes	***	0.0007
5	**FBS** vs. **IL27**	-2.231	-5.418 to 0.9555	No	ns	0.1530

IFI16 TPM DATA

Normality and Lognormality Tests	A	B	C	D	E	F
	**HS**	**IFN-I (IFNα)**	**IFN-II (IFNγ)**	**IFN-III (IFNλ)**	**FBS**	**IL27**
1 **Test for normal distribution**						
2 **Shapiro-Wilk test**						
3 W	0.8738	0.8915	0.9977	0.8901	0.8859	0.7662
4 P value	0.3065	0.3590	0.9083	0.3547	0.3420	0.0362
5 Passed normality test (alpha=0.05)?	Yes	Yes	Yes	Yes	Yes	No
6 P value summary	ns	ns	ns	ns	ns	*

	**Ordinary one-way ANOVA**<br>ANOVA results					
1	**ANOVA summary**					
2	F	176.7				
3	P value	<0.0001				
4	P value summary	****				
5	Significant diff. among means (P < 0.05)?	Yes				
6	R square	0.9866				
7						
8	**Brown-Forsythe test**					
9	F (DFn, DFd)	0.6737 (5, 12)				
10	P value	0.6514				
11	P value summary	ns				
12	Are SDs significantly different (P < 0.05)?	No				
13						
14	**ANOVA table**	**SS**	**DF**	**MS**	**F (DFn, DFd)**	**P value**
15	Treatment (between columns)	170911	5	34182	F (5, 12) = 176.7	P<0.0001
16	Residual (within columns)	2321	12	193.5		
18	Total	173232	17			

	**Ordinary one-way ANOVA**<br>Multiple comparisons					
1	**Uncorrected Fisher's LSD**	**Mean Diff.**	**95.00% CI of diff.**	**Significant?**	**Summary**	**Individual P Value**
2	**HS** vs. **IFN-I (IFNα)**	-265.4	-290.1 to -240.6	Yes	****	<0.0001
3	**HS** vs. **IFN-II (IFNγ)**	-156.3	-181.0 to -131.6	Yes	****	<0.0001
4	**HS** vs. **IFN-III (IFNλ)**	-82.03	-106.8 to -57.29	Yes	****	<0.0001
5	**FBS** vs. **IL27**	-17.49	-42.23 to 7.254	No	ns	0.1495

ZBP1 TPM DATA

Normality and Lognormality Tests		A	B	C	D	E	F
		HS	IFN-I (IFNα)	IFN-II (IFNγ)	IFN-III (IFNλ)	FBS	IL27
1	Test for normal distribution						
2	Shapiro-Wilk test						
3	W	0.7899	0.9896	0.9558	0.9847	0.9981	0.7617
4	P value	0.0906	0.8048	0.5956	0.7630	0.9161	0.0261
5	Passed normality test (alpha=0.05)?	Yes	Yes	Yes	Yes	Yes	No
6	P value summary	ns	ns	ns	ns	ns	*

Ordinary one-way ANOVA<br>ANOVA results						
1	**ANOVA summary**					
2	F	185.9				
3	P value	<0.0001				
4	P value summary	****				
5	Significant diff. among means (P < 0.05)?	Yes				
6	R square	0.9873				
7						
8	**Brown-Forsythe test**					
9	F (DFn, DFd)	1.932 (5, 12)				
10	P value	0.1623				
11	P value summary	ns				
12	Are SDs significantly different (P < 0.05)?	No				
13						
14	**ANOVA table**	**SS**	**DF**	**MS**	**F (DFn, DFd)**	**P value**
15	Treatment (between columns)	9552	5	1910	F (5, 12) = 185.9	P<0.0001
16	Residual (within columns)	123.3	12	10.28		
18	Total	9676	17			

Ordinary one-way ANOVA<br>Multiple comparisons						
1	**Uncorrected Fisher's LSD**	**Mean Diff.**	**95.00% CI of diff.**	**Significant?**	**Summary**	**Individual P Value**
2	**HS** vs. **IFN-I (IFNα)**	-65.77	-71.47 to -60.07	Yes	****	<0.0001
3	**HS** vs. **IFN-II (IFNγ)**	-2.802	-8.506 to 2.901	No	ns	0.3054
4	**HS** vs. **IFN-III (IFNλ)**	-16.05	-21.76 to -10.35	Yes	****	<0.0001
5	**FBS** vs. **IL27**	-8.677	-14.38 to -2.973	Yes	**	0.0062

RIG-I TPM DATA							
Normality and Lognormality Tests		A	B	C	D	E	F
		HS	IFN-I (IFNα)	IFN-II (IFNy)	IFN-III (IFNλ)	FBS	IL27
1	**Test for normal distribution**						
2	**Shapiro-Wilk test**						
3	W	0.8131	0.9978	0.9423	0.9365	0.9526	0.9483
4	P value	0.1461	0.9103	0.5367	0.5135	0.5808	0.5620
5	Passed normality test (alpha=0.05)?	Yes	Yes	Yes	Yes	Yes	Yes
6	P value summary	ns	ns	ns	ns	ns	ns

Ordinary one-way ANOVA<br>ANOVA results						
1	**ANOVA summary**					
2	F	515.3				
3	P value	<0.0001				
4	P value summary	****				
5	Significant diff. among means (P < 0.05)?	Yes				
6	R square	0.9954				
7						
8	**Brown-Forsythe test**					
9	F (DFn, DFd)	3.622 (5, 12)				
10	P value	0.0314				
11	P value summary	*				
12	Are SDs significantly different (P < 0.05)?	Yes				
13						
14	**ANOVA table**	**SS**	**DF**	**MS**	**F (DFn, DFd)**	**P value**
15	Treatment (between columns)	305901	5	61180	F (5, 12) = 515.3	P<0.0001
16	Residual (within columns)	1425	12	118.7		
18	Total	307325	17			

Ordinary one-way ANOVA<br>Multiple comparisons						
1	**Uncorrected Fisher's LSD**	**Mean Diff.**	**95.00% CI of diff.**	**Significant?**	**Summary**	**Individual P Value**
2	**HS** vs. **IFN-I (IFNα)**	-363.9	-383.3 to -344.5	Yes	****	<0.0001
3	**HS** vs. **IFN-II (IFNy)**	-25.45	-44.83 to -6.062	Yes	*	0.0143
4	**HS** vs. **IFN-III (IFNλ)**	-124.6	-144.0 to -105.3	Yes	****	<0.0001
5	**FBS** vs. **IL27**	-10.12	-29.51 to -9.261	Yes	*	0.0277

MDA5 TPM DATA							
Normality and Lognormality Tests		A	B	C	D	E	F
		HS	IFN-I (IFNα)	IFN-II (IFNγ)	IFN-III (IFNλ)	FBS	IL27
1	**Test for normal distribution**						
2	**Shapiro-Wilk test**						
3	W	0.8287	0.9977	0.9929	0.9988	0.9628	0.9879
4	P value	0.1850	0.9087	0.8391	0.9329	0.6294	0.7891
5	Passed normality test (alpha=0.05)?	Yes	Yes	Yes	Yes	Yes	Yes
	P value summary	ns	ns	ns	ns	ns	ns

**Ordinary one-way ANOVA** ANOVA results						
1	**ANOVA summary**					
2	F	909.2				
3	P value	<0.0001				
4	P value summary	****				
5	Significant diff. among means (P < 0.05)?	Yes				
6	R square	0.9974				
7						
8	**Brown-Forsythe test**					
9	F (DFn, DFd)	2.812 (5, 12)				
10	P value	0.0660				
11	P value summary	ns				
12	Are SDs significantly different (P < 0.05)?	No				
13						
14	**ANOVA table**	**SS**	**DF**	**MS**	**F (DFn, DFd)**	**P value**
15	Treatment (between columns)	421560	5	84312	F (5, 12) = 909.2	P<0.0001
16	Residual (within columns)	1113	12	92.73		
18	Total	422673	17			

**Ordinary one-way ANOVA** Multiple comparisons						
1	**Uncorrected Fisher's LSD**	**Mean Diff.**	**95.00% CI of diff.**	**Significant?**	**Summary**	**Individual P Value**
2	**HS** vs. **IFN-I (IFNα)**	-435.0	-452.1 to -417.8	Yes	****	<0.0001
3	**HS** vs. **IFN-II (IFNγ)**	-116.3	-133.4 to -99.12	Yes	****	<0.0001
4	**HS** vs. **IFN-III (IFNλ)**	-145.7	-162.8 to -128.5	Yes	****	<0.0001
5	**FBS** vs. **IL27**	-25.16	-42.29 to -8.029	Yes	**	0.0076

IFIT1 TPM DATA							
Normality and Lognormality Tests		A	B	C	D	E	F
		HS	IFN-I (IFNα)	IFN-II (IFNγ)	IFN-III (IFNλ)	FBS	IL27
1	**Test for normal distribution**						
2	**Shapiro-Wilk test**						
3	W	0.9464	0.9511	0.9609	0.8584	0.8823	0.9973
4	P value	0.5538	0.5741	0.6197	0.2631	0.3312	0.9016
5	Passed normality test (alpha=0.05)?	Yes	Yes	Yes	Yes	Yes	Yes
6	P value summary	ns	ns	ns	ns	ns	ns

**Ordinary one-way ANOVA**<br>ANOVA results						
1	**ANOVA summary**					
2	F	69642				
3	P value	<0.0001				
4	P value summary	****				
5	Significant diff. among means (P < 0.05)?	Yes				
6	R square	1.000				
7						
8	**Brown-Forsythe test**					
9	F (DFn, DFd)	1.068 (5, 12)				
10	P value	0.4248				
11	P value summary	ns				
12	Are SDs significantly different (P < 0.05)?	No				
13						
14	**ANOVA table**	**SS**	**DF**	**MS**	**F (DFn, DFd)**	**P value**
15	Treatment (between columns)	5361594	5	1072319	F (5, 12) = 69642	P<0.0001
16	Residual (within columns)	184.8	12	15.40		
18	Total	5361779	17			

**Ordinary one-way ANOVA**<br>Multiple comparisons						
1	**Uncorrected Fisher's LSD**	**Mean Diff.**	**95.00% CI of diff.**	**Significant?**	**Summary**	**Individual P Value**
2	**HS** vs. **IFN-I (IFNα)**	-1491	-1498 to -1484	Yes	****	<0.0001
3	**HS** vs. **IFN-II (IFNγ)**	-19.50	-26.49 to -12.52	Yes	****	<0.0001
4	**HS** vs. **IFN-III (IFNλ)**	-484.5	-491.5 to -477.6	Yes	****	<0.0001
5	**FBS** vs. **IL27**	-8.270	-15.25 to -1.289	Yes	*	0.0240

PKR TPM DATA

Normality and Lognormality Tests		A	B	C	D	E	F
		HS	IFN-I (IFNα)	IFN-II (IFNγ)	IFN-III (IFNλ)	FBS	IL27
1	**Test for normal distribution**						
2	**Shapiro-Wilk test**						
3	W	0.9981	0.9574	0.9235	0.8094	0.9838	0.8074
4	P value	0.9159	0.6032	0.4648	0.1371	0.7560	0.1323
5	Passed normality test (alpha=0.05)?	Yes	Yes	Yes	Yes	Yes	Yes
6	P value summary	ns	ns	ns	ns	ns	ns

**Ordinary one-way ANOVA**<br>ANOVA results						
1	**ANOVA summary**					
2	F	2421				
3	P value	<0.0001				
4	P value summary	****				
5	Significant diff. among means (P < 0.05)?	Yes				
6	R square	0.9990				
7						
8	**Brown-Forsythe test**					
9	F (DFn, DFd)	0.2552 (5, 12)				
10	P value	0.9290				
11	P value summary	ns				
12	Are SDs significantly different (P < 0.05)?	No				
13						
14	**ANOVA table**	**SS**	**DF**	**MS**	**F (DFn, DFd)**	**P value**
15	Treatment (between columns)	90105	5	18021	F (5, 12) = 2421	P<0.0001
16	Residual (within columns)	89.34	12	7.445		
18	Total	90195	17			

**Ordinary one-way ANOVA**<br>Multiple comparisons						
1	**Uncorrected Fisher's LSD**	**Mean Diff.**	**95.00% CI of diff.**	**Significant?**	**Summary**	**Individual P Value**
2	**HS** vs. **IFN-I (IFNα)**	-189.0	-193.8 to -184.1	Yes	****	<0.0001
3	**HS** vs. **IFN-II (IFNγ)**	-27.14	-31.99 to -22.29	Yes	****	<0.0001
4	**HS** vs. **IFN-III (IFNλ)**	-109.0	-113.8 to -104.1	Yes	****	<0.0001
5	**FBS** vs. **IL27**	-14.25	-19.10 to -9.393	Yes	****	<0.0001

CHIKUNGUNYA VIRUS REPLICATION

Normality and Lognormality Tests		A	B	C	D	E	F
		Uninfected	FBS	IFN-I (IFNβ)	IFN-II (IFNγ)	IFN-III (IFNλ)	IL27
1	**Test for normal distribution**						
2	**Shapiro-Wilk test**						
3	W	0.6298	0.8006	0.8035	0.9251	0.6501	0.9091
4	P value	0.0012	0.1032	0.1127	0.5660	0.0026	0.4775
5	Passed normality test (alpha=0.05)?	No	Yes	Yes	Yes	No	Yes
6	P value summary	**	ns	ns	ns	**	ns

Ordinary one-way ANOVA<br>ANOVA results						
1	**ANOVA summary**					
2	F	38.22				
3	P value	<0.0001				
4	P value summary	****				
5	Significant diff. among means (P < 0.05)?	Yes				
6	R square	0.9139				
7						
8	**Brown-Forsythe test**					
9	F (DFn, DFd)	1.308 (5, 18)				
10	P value	0.3046				
11	P value summary	ns				
12	Are SDs significantly different (P < 0.05)?	No				
13						
14	**ANOVA table**	**SS**	**DF**	**MS**	**F (DFn, DFd)**	**P value**
15	Treatment (between columns)	42393319640	5	8478663928	F (5, 18) = 38.22	P<0.0001
16	Residual (within columns)	3993580000	18	221865556		
18	Total	46386899640	23			

Ordinary one-way ANOVA<br>Multiple comparisons						
1	**Uncorrected Fisher's LSD**	**Mean Diff.**	**95.00% CI of diff.**	**Significant?**	**Summary**	**Individual P Value**
2	**FBS** vs. **IFN-I (IFNβ)**	113200	91072 to 135328	Yes	****	<0.0001
3	**FBS** vs. **IFN-II (IFNγ)**	115625	93497 to 137753	Yes	****	<0.0001
4	**FBS** vs. **IFN-III (IFNλ)**	112375	90247 to 134503	Yes	****	<0.0001
5	**FBS** vs. **IL27**	79250	57122 to 101378	Yes	****	<0.0001

DENGUE VIRUS 2 REPLICATION							
Normality and Lognormality Tests		A	B	C	D	E	F
		Uninfected	FBS	IFN-I (IFNβ)	IFN-II (IFNγ)	IFN-III (IFNλ)	IL27
1	**Test for normal distribution**						
2	**Shapiro-Wilk test**						
3	W	0.6298	0.8638	0.8298	0.6298	0.9930	0.9850
4	P value	0.0012	0.2741	0.2012	0.0012	0.9721	0.9308
5	Passed normality test (alpha=0.05)?	No	Yes	Yes	No	Yes	Yes
6	P value summary	**	ns	ns	**	ns	ns

Ordinary one-way ANOVA<br>ANOVA results						
1	**ANOVA summary**					
2	F	18.84				
3	P value	<0.0001				
4	P value summary	****				
5	Significant diff. among means (P < 0.05)?	Yes				
6	R square	0.8396				
7						
8	**Brown-Forsythe test**					
9	F (DFn, DFd)	2.137 (5, 18)				
10	P value	0.0529				
11	P value summary	ns				
12	Are SDs significantly different (P < 0.05)?	No				
13						
14	**ANOVA table**	**SS**	**DF**	**MS**	**F (DFn, DFd)**	**P value**
15	Treatment (between columns)	13568130203227	5	2713626040645	F (5, 18) = 18.84	P<0.0001
16	Residual (within columns)	2592650000002	18	144036111111		
18	Total	16160780203229	23			

Ordinary one-way ANOVA<br>Multiple comparisons						
1	**Uncorrected Fisher's LSD**	**Mean Diff.**	**95.00% CI of diff.**	**Significant?**	**Summary**	**Individual P Value**
2	**FBS** vs. **IFN-I (IFNβ)**	2112400	1548593 to 2676207	Yes	****	<0.0001
3	**FBS** vs. **IFN-II (IFNγ)**	2112400	1548593 to 2676207	Yes	****	<0.0001
4	**FBS** vs. **IFN-III (IFNλ)**	1590000	1026193 to 2153807	Yes	****	<0.0001
5	**FBS** vs. **IL27**	1352500	788693 to 1916307	Yes	****	<0.0001
